# Supplementary material for: Trade-offs between overall survival and side effects in the treatment of metastatic breast cancer: eliciting preferences of patients with primary and metastatic breast cancer using a discrete choice experiment
Source: BMJ Open. 2024 Apr 28;14(4):e076798. doi: 10.1136/bmjopen-2023-076798 (PMC11057309; doi:10.1136/bmjopen-2023-076798)
Supplement: Supplementary data [file bmjopen-2023-076798supp001.pdf]

Trade-offs between overall survival and side effects in the treatment of metastatic breast cancer: eliciting preferences of patients with primary and metastatic breast cancer using a discrete choice experiment'

Supporting Information 1

Qualitative Methods

Qualitative Literature Review

Embase and Medline were searched using the Ovid search engine. We aimed to identify literature which explored the patient perspective of cancer and the associated treatments. Search terms were designed to identify studies that (1) involved interviews/focus groups (2) explored patient attitudes/perspectives (3) focused on advanced or locally advanced cancer. We included all metastatic cancers given the scarcity of metastatic breast cancer-specific literature.

The search identified 434 results. Abstracts were screened and papers were excluded if they didn't reflect the underlying motivation of the search strategy. Studies were also excluded if: they focussed on an intervention which was not clinically supported or was not medicine (e.g., alternative medicine and exercise respectively); the study focus was seldom relevant to breast cancer (e.g., breathing complications brought on lung tumours). After abstract screening 83 studies remained after which 5 additional studies were excluded after reading beyond the abstract. The remaining papers were evaluated and findings which offered insight into determinants of a patient's quality of life or preference for treatment were identified. Findings were compiled and condensed into a report summarising what the available research to date suggested determining patient preferences and well-being.

Pain was among the most prominent topics of discussion. Respondents who had experienced cancer pain identified it as the most disturbing and limiting symptom of their illness (Luoma and Hakamies-Blomqvist, 2004). Patients with pain often reported extreme negative emotions (Lewis et al, 2015), loss of independence (Gibbins et al, 2014), and a desire for assisted death (Koffman et al, 2008). Other frequently explored topics included physical functioning and mobility which, as concepts, are closely linked to pain (Wilson et al, 2005). The symptoms of disease and the side effects of treatment which led to degraded physical functioning levels were identified as substantial barriers to a patient's ability to live a normal life (Gibbins et al, 2014). Extreme degradation of mobility leads to increased dependence on loved ones and carers which can create a strong sense of burden (Mak, and Elwyn, 2005). Cognitive functioning also appears to have been a topic of interest for qualitative researchers. Although cognitive functioning appears to have been a significant area of interest, many metastatic breast cancer patients rarely had symptoms, when they did, they presented as secondary disturbances or anxieties (Luoma and Hakamies-Blomqvist, 2004). Patients were willing to take medications which were associated with drowsiness to alleviate symptoms of pain (Check et al, 2017). This is evidence that patients already accept trade-offs between symptoms when considering treatments. Evidence of similar trade-offs was also found between: hot flushes and mode of administration (Fallowfield et al, 2005), expected survival and physical functioning (Check et al, 2017), and expected survival against the collective side effects of chemotherapy (Etkind et al, 2017). Evidence of trade-offs between symptoms and side effects tells us something about the importance of those toxicities, but more importantly, helps to validate the decisional context we use to frame our DCE survey questions. Other themes which featured heavily in the literature were the topics of survival, fatigue, and mode of administration, all of which are discussed in more detail in section 4 of this paper.

DCE Literature Review

The benefits of reviewing DCEs with similar motivations to our study are twofold. Firstly, they can offer insight into the importance of some of the treatment factors which we would be considering.

Secondly, DCEs often employ rigorous qualitative processes and their choice of attributes is likely to be of interest because their selection implicitly suggests significance. In the context of a cancer treatment DCE an attribute would be a feature of treatment which has the potential to vary between competing hypothetical treatments in a choice task. Embase and Medline were searched for DCE studies relating to patient preference for metastatic cancer treatments[1]. Search terms designed to identify DCEs mirrored those first used by Ryan and Gerard (2003). We also reincorporated the search terms used to identify metastatic cancer studies used in the qualitative literature review. Once again preliminary searches revealed that there was an insufficient body of publications to focus on metastatic breast cancer studies alone. 128 unique studies were identified in total. After screening the abstracts 60 papers met the eligibility criteria. There were 16 instances where two studies reported the results from the same DCE, in these instances the most recent publication was selected. 44 studies were identified as meeting all the criteria. Once the papers were identified work began to analyse the attributes used by the studies. The WP produced 2 key outputs of interest (1) an outline of the types of attributes used in similar past DCEs and (2) their relative importance.

Attributes were grouped into categories with similar motives. The table below outlines the attribute categories which featured in more than one DCE. There were instances where one DCE contained more than one attribute which could fit into the same category, in which instance only one was counted.

Table 1 Frequency of attribute categories included in the DCE literature review

**Attribute Category Frequency**	**n**
Administration	12
Progression Free Survival	12
Cost	8
Overall Survival	8
Pain	7
Fatigue	5
Gastrointestinal Perforation	3
Kidneys	3
Skin	3
Teeth/jaw	3
Adverse Events	2
Bone Metastases	2
Diarrhoea	2
Hospitalisation	2
Immunosuppression	2
Nausea	2
Neuropathy	2
Response rates	2
Self-care	2

Relative preference weights are measures of the importance of attributes relative to competing attributes and are conditional on the range of utility estimates for the remaining attributes (Hauber et al, 2016). A large relative preference weight suggests that an attribute has high importance in the

[1] the number metastatic breast cancer specific studies identified in preliminary searches were insufficient to justify their own review

context of the DCE's design. The selection of competing attributes, the range of levels for the attribute and its competitors, and framing effects (Howard and Salkeld, 2009) all determine the scale of a relative preference weight. Nevertheless, underlying preference is still a key determinant of relative preference weights and, if the considerations are accounted for, valuable inferences are possible. When making comparisons between DCEs differing study designs should be considered including decisional context, the motivations of the studies, statistical methods, and sample compositions. The complexity of these comparisons means they can't be definitively relied upon, nonetheless they are useful when consolidated with additional information from other WPs.

The main finding of the DCE literature review was the prevalence of certain attributes among the DCEs, furthermore, certain attributes tended to be associated with high relative importance between DCEs. The closely related attributes of progression free survival (PFS) and overall survival (OS) were both frequently included and tended to have high relative importance, the significance of survival and the relationship between these variations will be explored in more depth in section 4 of this paper. Pain was another category of attribute which was frequently explored and tended to be associated with high relative importance, this suggests a strong preference amongst patients to minimise suffering. It is also worth noting that, many studies appeared to be interested in patients' preferences for mode of administration, although it appeared respondents often prioritised other attributes. As a final note, the relative importance of many symptoms and side effects such as fatigue, nausea and diarrhoea differed greatly between DCEs, it was here that the limitations of making deductions from the results DCEs with different objectives were most apparent.

PACE Statement Thematic Analysis

We were granted access by the Scottish Medicines Consortium (SMC) to eight PACE statements relating to metastatic breast cancer treatments. The SMC is Scotland's advisory body for medicines, as part of their drug approval process for ultra-orphan and end-of-life medicines they invite patient and clinical representatives to meetings to discuss the benefits. These are known as Patient and Clinical Engagement (PACE) meetings. PACE meetings aim to consider all available and relevant evidence regarding new medicines including factors which traditional economic evaluation tends to overlook. We identified PACE statements as a potentially useful secondary data resource for our research since their focus is on the needs of the patient. Another advantage is that PACE statements are a relatively recent innovation meaning they tend to present up-to-date information. Between Oct 2014 and Oct 2018, eight PACE meetings were convened for medicines seeking reimbursement for the treatment of metastatic breast cancer. We conducted a formal thematic analysis (Braun and Clarke, 2012) of the PACE statements which focussed on the positive and negative impacts of treatment as well the insights into patient priorities.

We were able to identify six core themes which were composed of additional sub-themes (see figure below). Themes were not mutually exclusive, meaning there is some degree of overlap between themes. Two of the themes represent what we came to understand as the core goals of patients according to the data, these were 'Ability to live a normal life' and 'Survival'; treatments were praised repeatedly by committees for their ability to improve these two outcomes. When consulting the evidence from the PACE analysis it should be considered that they are designed to consider externalities and not just the direct effect on patients. Specifically, PACE guidelines request that respondents discuss the effect of disease and treatment on the family and carers. This explains the prominence of the 'effect on close ones' theme which is often featured in the form of considering perspectives outside of the patients. Although the findings were interesting for our research, we decided to focus on the perspective of the patient. So naturally, this theme emerged. A key disadvantage of PACE statements was their tendency to talk broadly and generally about symptoms and side effects. For our research, we were interested in patients' preferences for specific symptoms and side effects, but the lack of detail meant little could be deduced about which common side effects were more troublesome than others. It should also be noted that PACE statements are rarely critical of

emerging drugs. The general feeling from the PACE statements was that participants were keen to highlight the benefits of emerging drugs. There was a positive bias that we had to consider when toxicities and benefits associated with the treatment in question were mentioned

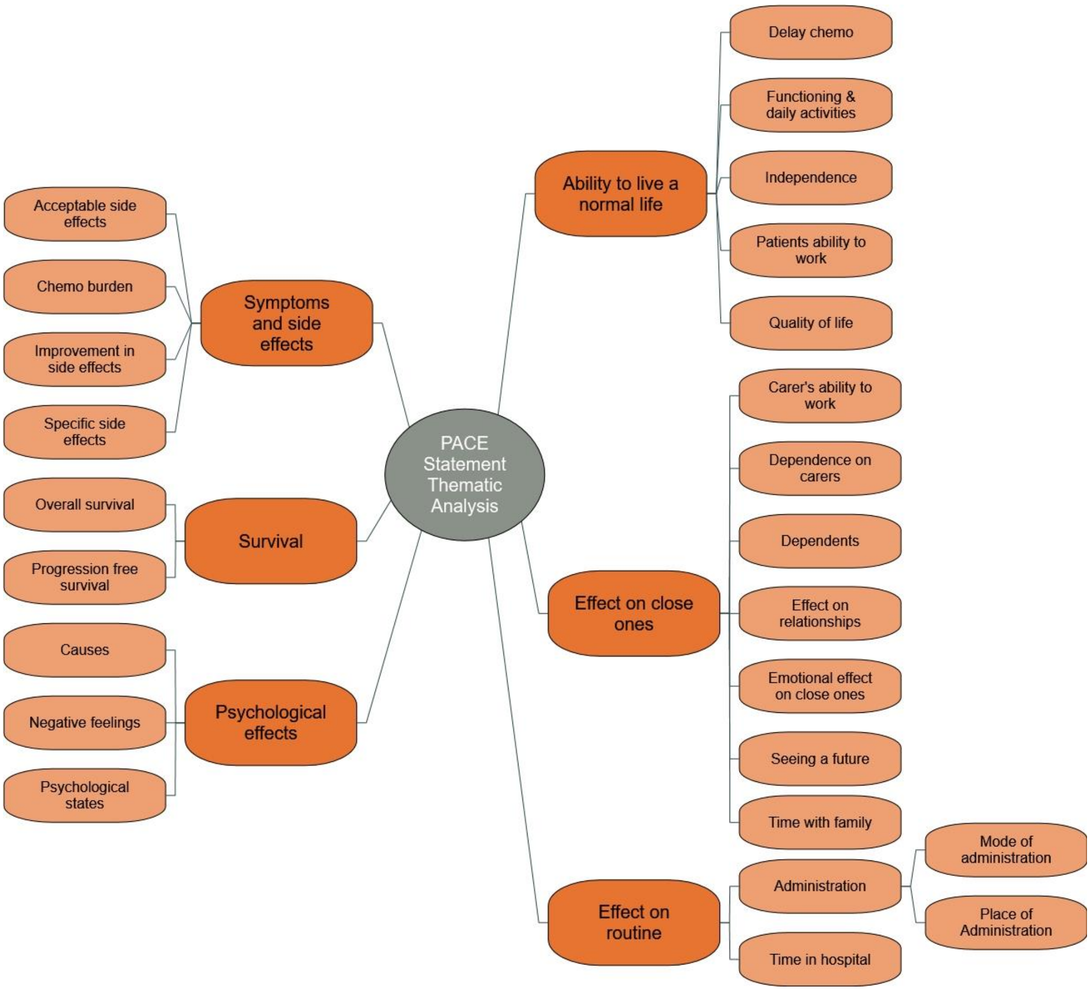

Figure 1 – Results from thematic analysis of breast cancer PACE data

Patient Interviews

The richest data from the early stages of the project emerged from the semi-structured interviews we conducted with 9 patients diagnosed with metastatic breast cancer. Women with secondary breast cases with experience of multiple treatments and who were currently living in the Lothian area were contacted by a research nurse and invited to participate in a face-to-face interview at an agreed location, either a cancer charity premises or the patient's home. We wanted to adopt a flexible strategy where we could adapt individual interviews and our broader strategies as our understanding of patient preferences and experiences developed. Grounded theory (Strauss and Corbin, 1994) is a qualitative methodology that encourages a flexible strategy, however, conventional recommendations state that interviewers should be mostly ignorant about the topic being explored so that bias does not interfere with the formulation of theories. Given that we already had considerable knowledge of the experiences of breast cancer patients, owing to ongoing research and professional experience, we instead opted to conduct interviews according to the informed grounded theory approach (Thornberg,

2012). This adaptation of the grounded theory methodology allowed us to incorporate our prior knowledge in the traditional grounded theory approach whilst being aware of bias and remaining open to new ideas. An interview plan was formulated which provided structure whilst allowing for deviation and elaboration. The three core areas of focus were (1) patient history – patients were invited to discuss the treatments they had received and reflect on their experiences with them (2) treatment decision making – patient were asked how they remember decisions about treatment being and to reflect on the extent of their own involvement (3) experience with treatment and disease – patients were asked to reflect on their lived experience of their disease and their treatment and how it affected them.

To summarise the broader findings: There was a general attitude that more treatment was generally better and that listening to the advice of health professionals is the best thing one can do. There was a large degree of variation in terms of the specific side effects that patients' experiences and to what extent. This is likely a consequence of the wide range of secondary malignancies and the treatments received. Several patients mentioned suffering very little from symptoms and side effects since their secondary diagnosis. There was a prevailing negative attitude towards chemotherapy and its associated toxicities. The two primary goals of treatment appeared to be life extension and minimising disruption to everyday life. The interviews helped us to understand the broader goals of patients as well as their self-reported attitudes and behaviours regarding shared decision making. The richest findings however related to discussions concerning specific symptoms and side effects, evidence from these discussions will feature heavily in section 4 of this paper.

References

Braun, V. and Clarke, V., 2012. Thematic analysis.

Check, D. K., Park, E. M., Reeder-Hayes, K. E., Mayer, D. K., Deal, A. M., Yopp, J. M., … Hanson, L. C. (2017). Concerns underlying treatment preferences of advanced cancer patients with children. Psycho-Oncology, 26(10), 1491–1497.

Etkind, S.N., Bristowe, K., Bailey, K., Selman, L.E. and Murtagh, F.E., 2017. How does uncertainty shape patient experience in advanced illness? A secondary analysis of qualitative data. Palliative medicine, 31(2), pp.171-180.

Gibbins, J., Bhatia, R., Forbes, K., & Reid, C. M. (2014). What do patients with advanced incurable cancer want from the management of their pain? A qualitative study. Palliative Medicine, 28(1), 71–78.

Hauber, A.B., González, J.M., Groothuis-Oudshoorn, C.G., Prior, T., Marshall, D.A., Cunningham, C., IJzerman, M.J. and Bridges, J.F., 2016. Statistical methods for the analysis of discrete choice experiments: a report of the ISPOR Conjoint Analysis Good Research Practices Task Force. Value in health, 19(4), pp.300-315.

Howard, K. and Salkeld, G., 2009. Does attribute framing in discrete choice experiments influence willingness to pay? Results from a discrete choice experiment in screening for colorectal cancer. Value in health, 12(2), pp.354-363.

Koffman, J., Morgan, M., Edmonds, P., Speck, P., & Higginson, I. (2008). Cultural meanings of pain: A qualitative study of Black Caribbean and White British patients with advanced cancer. Palliative Medicine, 22(4), 350–359.

Lewis, S., Yee, J., Kilbreath, S. and Willis, K., 2015. A qualitative study of women's experiences of healthcare, treatment and support for metastatic breast cancer. The Breast, 24(3), pp.242-247.

Luoma, M.L. and Hakamies-Blomqvist, L., 2004. The meaning of quality of life in patients being treated for advanced breast cancer: a qualitative study. Psycho-Oncology: Journal of the Psychological, Social and Behavioral Dimensions of Cancer, 13(10), pp.729-739.

Mak, Y.Y.W. and Elwyn, G., 2005. Voices of the terminally ill: uncovering the meaning of desire for euthanasia. Palliative medicine, 19(4), pp.343-350.

Ryan, M. and Gerard, K., 2003. Using discrete choice experiments to value health care programmes: current practice and. Applied health economics and health policy, 2(1), pp.55-64.Hauber, A.B.,

Strauss, A. and Corbin, J., 1994. Grounded theory methodology. Handbook of qualitative research, 17, pp.273-85.

Thornberg, R., 2012. Informed grounded theory. Scandinavian Journal of Educational Research, 56(3), pp.243-259.

Wilson, K. G., Curran, D., & McPherson, C. J. (2005). A burden to others: A common source of distress for the terminally ill. Cognitive Behaviour Therapy, 34(2), 115–123
