# Supplementary material for: Trade-offs between overall survival and side effects in the treatment of metastatic breast cancer: eliciting preferences of patients with primary and metastatic breast cancer using a discrete choice experiment
Source: BMJ Open. 2024 Apr 28;14(4):e076798. doi: 10.1136/bmjopen-2023-076798 (PMC11057309; doi:10.1136/bmjopen-2023-076798)
Supplement: Supplementary data [file bmjopen-2023-076798supp002.pdf]

Supporting Information 2

Table A1 Presentation of Side Effects Attribute Levels to Respondents

**Tiredness**	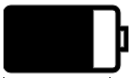<br><u>Even with best supportive treatment and care, there will be weeks that you experience the following…</u><br>No increase in tiredness. Your cancer makes you more tired than before, but this is relieved by rest.	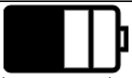<br><u>Even with best supportive treatment and care, there will be weeks that you experience the following…</u><br>You are much more tired than usual, your tiredness is not relieved by rest, and it limits your ability to perform some of your important daily activities.	
**Nausea and Vomiting**	**No nausea and vomiting**	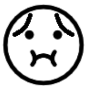<br><u>Even with best supportive treatment and care, there will be weeks that you experience the following…</u><br>You have lost your appetite due to nausea, but not enough to change the amount you eat. Your nausea may cause some vomiting.	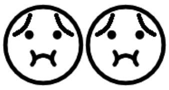<br><u>Even with best supportive treatment and care, there will be weeks that you experience the following…</u><br>The amount you eat and drink is decreased because of nausea but you are not at high risk of major weight loss or dehydration. The nausea is likely to cause vomiting.
**Diarrhoea**	**No diarrhoea**	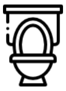<br><u>Even with best supportive treatment and care, there will be weeks that you experience the following…</u><br>You are having 2 more bowel movements a day than you were previously having.	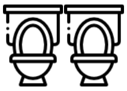<br><u>Even with best supportive treatment and care, there will be weeks that you experience the following…</u><br>You are having 5 more bowel movements a day than you were previously having and this limits your ability to perform some of your important daily activities.
**Additional Side effect**	**Peripheral neuropathy**<br>**No risk of hand foot syndrome or mucositis.**<br><u>Even with best supportive treatment and care, there will be weeks that you experience the following…</u><br>You have numbness and tingling in the feet or hands and occasionally burning, stabbing or shooting pain in affected areas. This limits your ability to perform some of your important daily activities.	**Hand foot syndrome**<br>**No risk of neuropathy or mucositis.**<br><u>Even with best supportive treatment and care, there will be weeks that you experience the following…</u><br>You have painful skin changes on the palms of your hands and the soles of your feet. This may include peeling, blisters, bleeding, dryness, cracking, calluses, and swelling. This limits your ability to perform some of your important daily activities.	**Mucositis**<br>**No risk of neuropathy or hand foot syndrome.**<br><u>Even with best supportive treatment and care, there will be weeks that you experience the following…</u><br>Your mouth becomes sore and inflamed. You have ulcers which are painful and mean you are unable to eat spicy, acidic, and crunchy foods such as crisps.
	**No risk of neuropathy, hand foot syndrome or mucositis**		

Table A2 Respondent characteristics

**Diagnosis**	**n**
Metastatic breast cancer	72
Primary breast cancer	33
**Gender**	
Female	105
Male	0
**Age**	
30-39	8
40-49	19
50-59	47
60-69	25
70-79	6

Table A3 Multinomial Results – Main Specification

		Estimate	p	95% CI Lower bound	95% CI Upper bound	Relative attribute importance	Minimum acceptable survival
Alternative Specific Constant	Treatment	0.9598	0.0006	0.4136	1.5060	-	-
Fatigue	Grade 2 fatigue	-0.2899	0.0089	-0.5073	-0.0726	0.0658	2.8017
Nausea	Grade 1 nausea	-0.3070	0.1021	-0.6750	0.0610	0.0951	2.9665 N.S.
	Grade 2 nausea	-0.4192	0.0232	-0.7811	-0.0573		4.0503
Diarrhoea	Grade 1 diarrhoea	0.0696	0.6425	-0.2242	0.3636	0.1536	-0.6734 N.S.
	Grade 2 diarrhoea	-0.6076	0.0011	-0.9715	-0.2438		5.8714
Additional side effects	Grade 2 peripheral neuropathy	-1.070	0.0000	-1.4654	-0.6748	0.2693	10.3399
	Grade 2 hand foot syndrome	-1.1873	0.0000	-1.5759	-0.7987		11.4723
	Grade 2 mucositis	-1.1264	0.0000	-1.4830	-0.7698		10.8842
Overall survival	Annual probability of survival	0.1035	0.0000	0.0764	0.1305	0.3521	-
Urgent Hospital Admission	Probability of urgent hospital admission in the first year of treatment	0.0097	0.0589	-0.0004	0.0198	0.0640 N.S.	-2.8223 N.S.(for 30% level)
**Model statistics**							
Number of individuals	105						
Observations	601						
Log likelihood	-431.59						
Bayesian info criterion	933.5637						

N.S. not significant

Table A4 Multinomial Results – Excluding Treat variable and paramatarising Overall Survival as Dummy Variables

Attribute	Level	Estimate	p	95% CI Lower bound	95% CI Upper bound
Fatigue	Grade 2 fatigue	-0.2887	0.0136	-0.5179	-0.0595
Nausea	Grade 1 nausea	-0.3084	0.1080	-0.6844	0.0677
	Grade 2 nausea	-0.4194	0.0232	-0.7814	-0.0574
Diarrhoea	Grade 1 diarrhoea	0.0668	0.6959	-0.2682	0.4019
	Grade 2 diarrhoea	-0.6036	0.0066	-1.0391	-0.1680
Additional side effects	Grade 2 peripheral neuropathy	-1.0758	0.0000	-1.5941	-0.5576
	Grade 2 hand foot syndrome	-1.1897	0.0000	-1.6034	-0.7761
	Grade 2 mucositis	-1.1269	0.0000	-1.4844	-0.7694
Overall survival (Annual probability of survival)	60%	2.5175	0.0000	1.9034	3.1316
	65%	3.0212	0.0000	2.2538	3.7887
	75%	4.0641	0.0000	3.2953	4.8329
Urgent Hospital Admission	Probability of urgent hospital admission in the first year of treatment	0.0100	0.2467	-0.0069	0.0268
**Model statistics**					
Number of individuals	105				
Observations	601				
Log likelihood	-431.59				
Bayesian info criterion	939.96				
