# Supplementary material for: Trade-offs between overall survival and side effects in the treatment of metastatic breast cancer: eliciting preferences of patients with primary and metastatic breast cancer using a discrete choice experiment
Source: BMJ Open. 2024 Apr 28;14(4):e076798. doi: 10.1136/bmjopen-2023-076798 (PMC11057309; doi:10.1136/bmjopen-2023-076798)
Supplement: Supplementary data [file bmjopen-2023-076798supp003.pdf]

Trade-offs between overall survival and side effects in the treatment of metastatic breast cancer: eliciting preferences of patients with primary and metastatic breast cancer using a discrete choice experiment'

Supporting Information 3

Figure A1 Comparison of relative importance estimates from multinomial logit models between the entire sample and 'survival non-traders' (sample excluding survival non-traders, N=71)

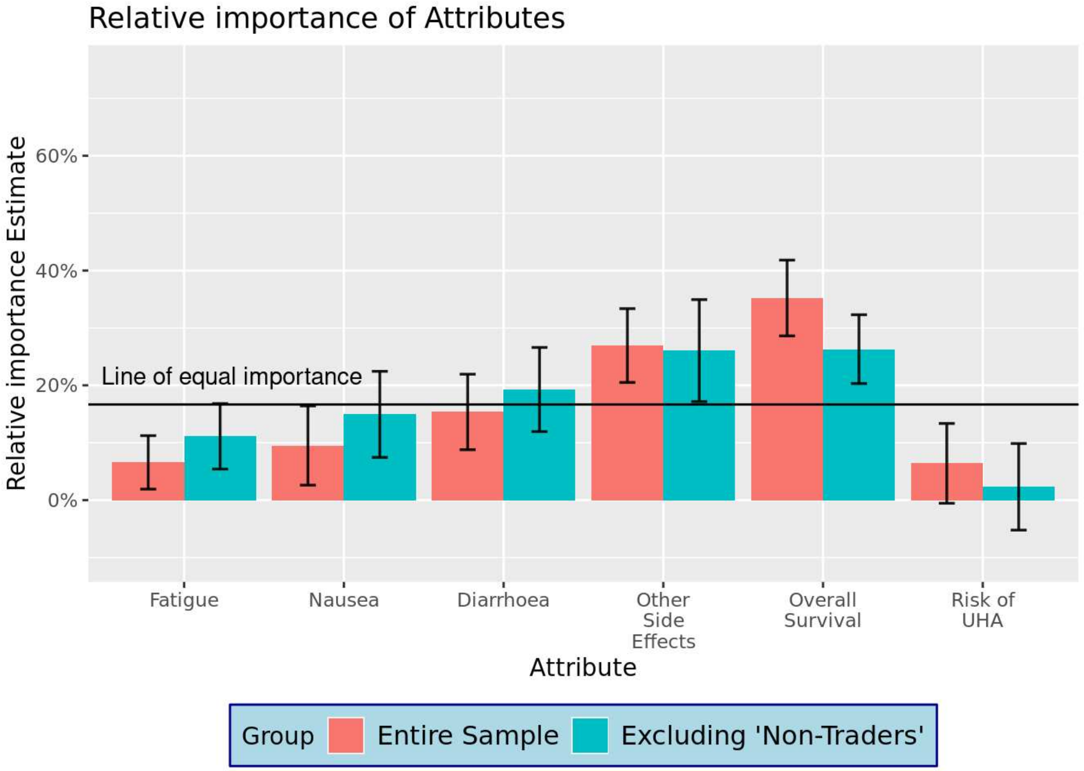

Error bars show 95% confidence interval using delta method standard errors

Figure A2 Comparison of minimum acceptable survival from multinomial logit models between the entire sample and 'survival non-traders' (sample excluding survival non-traders, N=71)

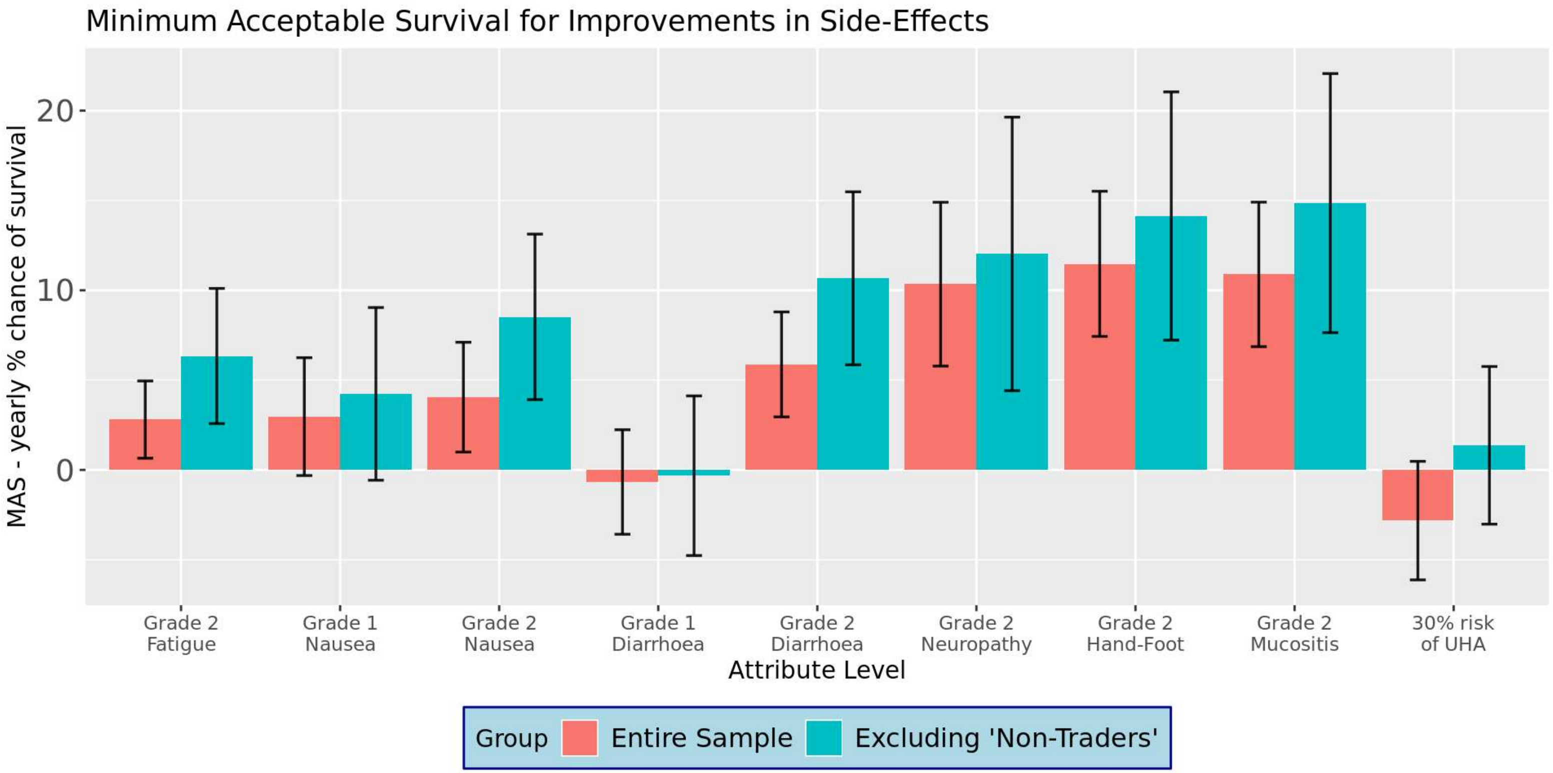

Error bars show 95% confidence interval using delta method standard errors

Figure A3 Comparison of Relative importance estimates from multinomial logit models between the metastatic breast cancer sample (N=72) and the primary breast cancer sample (N=33)

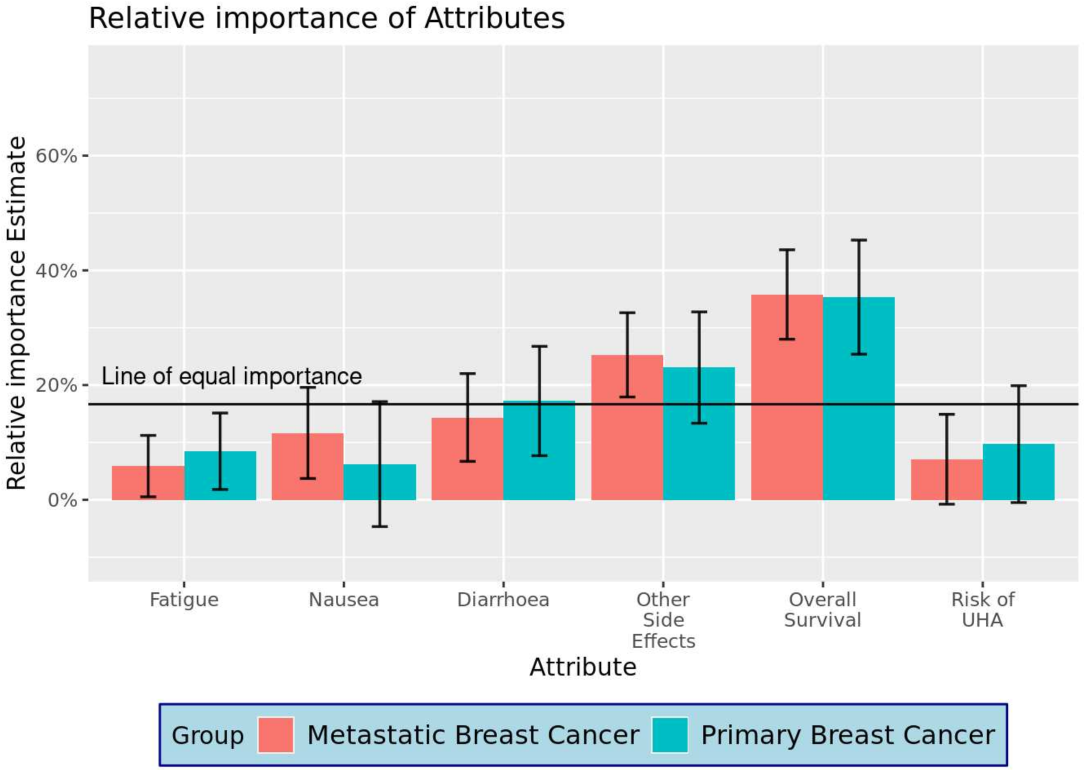

Error bars show 95% confidence interval using delta method standard errors

Figure A4 Comparison of Relative importance Estimates Between the Metastatic Breast Cancer Sample (N=72) and the Primary Breast Cancer Sample (N=33)

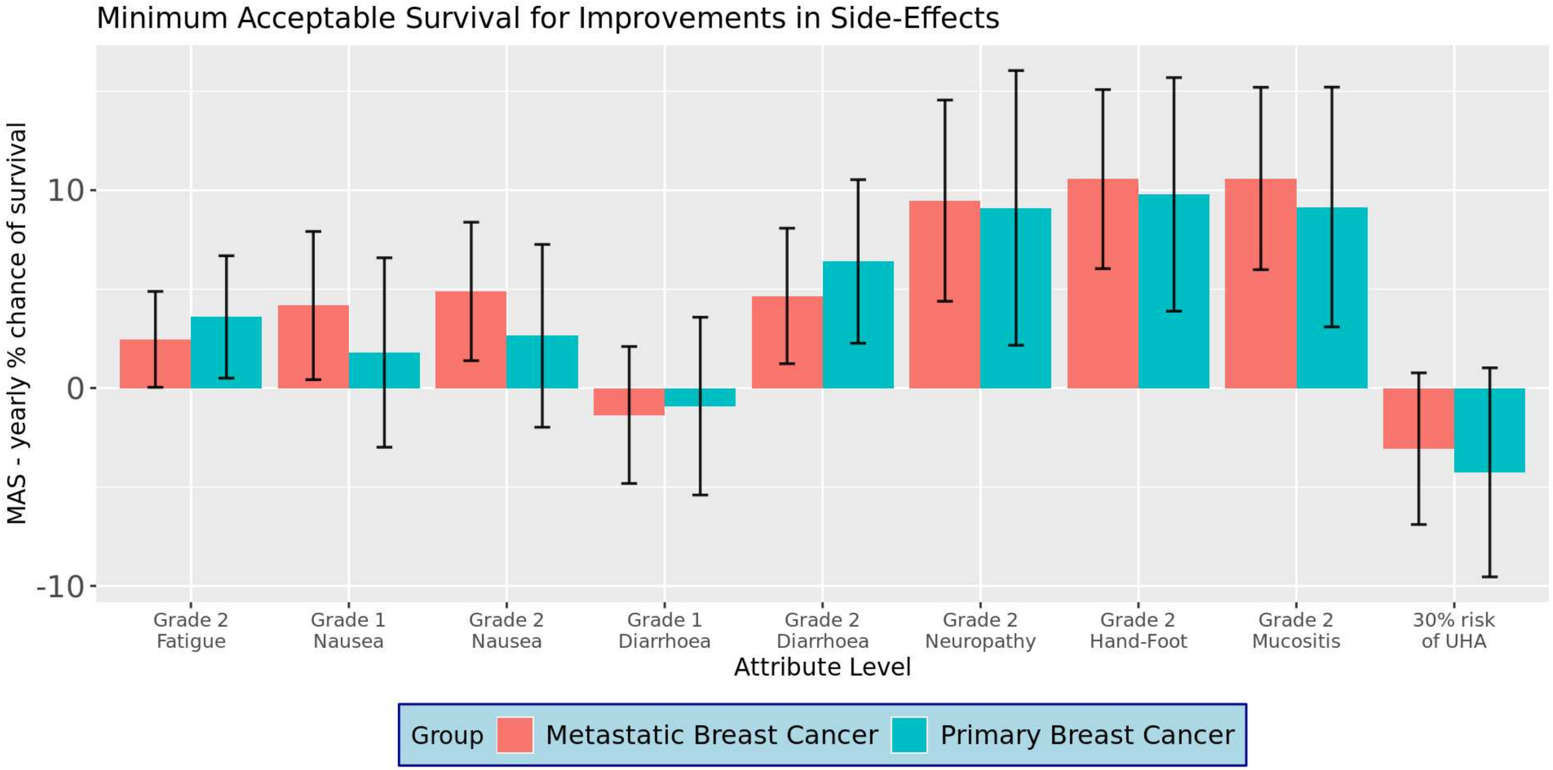

Error bars show 95% confidence interval using delta method standard errors
