# Supplementary material for: Trade-offs between overall survival and side effects in the treatment of metastatic breast cancer: eliciting preferences of patients with primary and metastatic breast cancer using a discrete choice experiment
Source: BMJ Open. 2024 Apr 28;14(4):e076798. doi: 10.1136/bmjopen-2023-076798 (PMC11057309; doi:10.1136/bmjopen-2023-076798)
Supplement: Author's manuscript [file bmjopen-2023-076798.draft_revisions.pdf]

BMJ Open

BMJ Open is committed to open peer review. As part of this commitment we make the peer review history of every article we publish publicly available.

When an article is published we post the peer reviewers' comments and the authors' responses online. We also post the versions of the paper that were used during peer review. These are the versions that the peer review comments apply to.

The versions of the paper that follow are the versions that were submitted during the peer review process. They are not the versions of record or the final published versions. They should not be cited or distributed as the published version of this manuscript.

BMJ Open is an open access journal and the full, final, typeset and author-corrected version of record of the manuscript is available on our site with no access controls, subscription charges or pay-per-view fees (http://bmjopen.bmj.com).

If you have any questions on BMJ Open's open peer review process please email info.bmjopen@bmj.com

BMJ Open

Women's preferences for overall survival versus avoiding side effects in the treatment of metastatic breast cancer: a discrete choice experiment

Journal:	*BMJ Open*
Manuscript ID	bmjopen-2023-076798
Article Type:	Original research
Date Submitted by the Author:	22-Jun-2023
Complete List of Authors:	Bullen, Alistair; The University of Edinburgh Usher Institute of Population Health Sciences and Informatics, Edinburgh Clinical Trials Unit<br>Ryan, Mandy; University of Aberdeen, HERU<br>Ennis, Holly; The University of Edinburgh<br>Gray, Ewan; The University of Edinburgh<br>Loría-Rebolledo, Luis Enrique ; University of Aberdeen, Health Economics Research Unit<br>McIntyre, Morag; Western General Hospital<br>Hall, Peter; The University of Edinburgh; Western General Hospital
Keywords:	Quality of Life, Decision Making, Surveys and Questionnaires, Breast tumours < ONCOLOGY, HEALTH ECONOMICS

SCHOLARONE™
Manuscripts

I, the Submitting Author has the right to grant and does grant on behalf of all authors of the Work (as defined in the below author licence), an exclusive licence and/or a non-exclusive licence for contributions from authors who are: i) UK Crown employees; ii) where BMJ has agreed a CC-BY licence shall apply, and/or iii) in accordance with the terms applicable for US Federal Government officers or employees acting as part of their official duties; on a worldwide, perpetual, irrevocable, royalty-free basis to BMJ Publishing Group Ltd ("BMJ") its licensees and where the relevant Journal is co-owned by BMJ to the co-owners of the Journal, to publish the Work in this journal and any other BMJ products and to exploit all rights, as set out in our licence.

The Submitting Author accepts and understands that any supply made under these terms is made by BMJ to the Submitting Author unless you are acting as an employee on behalf of your employer or a postgraduate student of an affiliated institution which is paying any applicable article publishing charge ("APC") for Open Access articles. Where the Submitting Author wishes to make the Work available on an Open Access basis (and intends to pay the relevant APC), the terms of reuse of such Open Access shall be governed by a Creative Commons licence – details of these licences and which Creative Commons licence will apply to this Work are set out in our licence referred to above.

Other than as permitted in any relevant BMJ Author's Self Archiving Policies, I confirm this Work has not been accepted for publication elsewhere, is not being considered for publication elsewhere and does not duplicate material already published. I confirm all authors consent to publication of this Work and authorise the granting of this licence.

For peer review only

Women's preferences for overall survival versus avoiding side effects in the treatment of metastatic breast cancer: a discrete choice experiment

Authors Alistair J Bullen[1*]

Mandy Ryan[2]

Holly Ennis[1]

Ewan Gray[1]

Luis E Loría-Rebolledo[2]

Morag McIntyre[3]

Peter S Hall[1,3]

Affiliations

1. Edinburgh Health Economics, University of Edinburgh, Edinburgh Clinical Trials Unit, 9 Little France Rd, Edinburgh, EH16 4UX, United Kingdom
2. Health Economics Research Unit, University of Aberdeen, Polwarth Building, Foresterhill, Aberdeen, AB25 2ZD, United Kingdom
3. Edinburgh Cancer Centre, NHS Lothian, Western General Hospital, Crewe Road South, Edinburgh, EH4 2XU, United Kingdom

* Corresponding author: alistair.bullen@ed.ac.uk

Conflicts of interest: All authors declare no conflicts of interest.

Keywords

Breast neoplasms; quality of life; decision making, shared; patient preference; surveys and questionnaires

3954 words

For peer review only

Abstract

Background: There has been a recent proliferation in treatment options for patients with metastatic breast cancer. Such treatments often involve trade-offs between overall survival and side effects. **Methods:** We designed a discrete choice experiment (DCE) to look at preferences for avoiding severity levels of side effects when choosing treatment for metastatic breast cancer. Treatment attributes were: fatigue; nausea; diarrhoea; other side effects (peripheral neuropathy, hand foot syndrome, and mucositis**);** urgent hospital admission and overall survival. Responses were analysed using a multinomial logit model. We estimated the relative importance of attributes and minimum acceptable survival for improvements in side effects. **Results:** 105respondents participated, comprising of 72 metastatic breast cancer patients and 33 primary breast cancer patients. Overall survival had the largest relative importance, followed by other side effects, diarrhoea, nausea, and fatigue. Risk of urgent hospital admission was not significant. Whilst overall survival was the most important attribute, respondents were willing to forgo some absolute probability of overall survival for reductions in all Grade 2 side effects (11.47% for hand foot syndrome; 10.88% for mucositis; 10.34% for peripheral neuropathy and 5.87% for diarrhoea). Grade 1 side effects were not significant, suggesting respondents were willing to tolerate them. **Conclusion:** Women are willing to forgo overall survival to avoid particular severity levels of side effects. Our results have implications for data collected in research studies and can help inform person-centred care and shared decision making.

For peer review only

Strengths and limitations of this study

- Our study is also the first to elicit preferences for the treatment of metastatic breast cancer in the United Kingdom.
- The attributes chosen for the discrete choice experiment are highly general and refer to side effects shared by a variety of treatments. They are useful for making general comparisons between a wide array of treatments but less applicable for more nuanced choices that might offer small differences and are associated with side effects we did not investigate.
- Due to difficulties recruiting participants we were required to use a joint sample of metastatic and primary breast cancer patients when ideally the primary sample would consist only of metastatic breast cancer patients

For peer review only

Introduction

There are 35,000 people in the UK living with metastatic breast cancer (mBC) (1). mBC occurs if the cancer spreads to another part of the body at which point the cancer is usually considered incurable. The focus of treatment then shifts from curing the disease to managing it, slowing further progression and palliating symptoms. There is a dichotomy at the core of discussions surrounding treatment in this context, namely the trade-off between overall survival (OS) and the side effects patients must tolerate (2). Different treatments offer variable prospects for survival versus side effects. Treatment decisions are made more complex by the proliferation of new medicines for the treatment of mBC, ranging from cytotoxic chemotherapy to hormone therapies. Recent new additional options include immunotherapy and targeted small molecules (3).

Such developments mean that breast cancer patients must navigate difficult decisions between complex and unfamiliar treatments (4). Greater patient involvement in decision-making is needed to allocate the treatment which best addresses their needs. Recent guidelines have emphasised the requirement for shared decision-making across the NHS (5, 6). Although shared decision-making is widely practised its implementation needs improvement, specifically regarding doctor-patient communication (7). Evidence from patient preference studies reveal trends to be considered by healthcare providers during consultations. Patient preferences are also important for the authors of healthcare guidelines that inform policy around which drugs should be provided. As a final example, they are important for developers of new cancer drugs when they provide guidance on what patients will tolerate concerning side effects for improvements in survival.

Discrete Choice Experiments (DCEs), sometimes referred to as conjoint analysis, are increasingly used to estimate patient preferences, looking at the relative importance of attributes as well as the trade-offs individuals are willing to make (8). A recent systematic review of the application of DCEs to oncology treatment identified 79 studies, with patient preferences for breast cancer (n =10, 13%) as the most common area of application (9). The review found the most common outputs were relative

For peer review only

importance of attributes and marginal rates of substitution (MRS, trade-offs) in terms of (in order of frequency): willingness to pay (WTP); minimum acceptable benefit; minimum acceptable risk; willingness to accept non-risk for benefit and willingness to travel. Whilst clinical efficacy attributes were commonly ranked as most important, with OS and PFS ranked most important by 90% and 30% respectively by patient samples across all cancer types, respondents were often willing to trade clinical efficacy for improvements in side effects. A similar result was found in a systematic review of patient preference studies relating to breast cancer treatment (10). These two systematic reviews identified six DCEs that assessed preferences for mBC drug treatments (11-16). These studies also show that whilst treatment efficacy (OS or PFS) is important, and often the most important factor, patients also value avoiding the side effects of different treatments (11, 14-16). Two of these mBC studies estimated the value of avoiding side effects in monetary terms (willingness to pay, a monetary measure of benefit) (13, 14). We use the DCE methodology to investigate how much absolute probability of OS women are willing to give up to avoid a particular severity level of side effects in the treatment of mBC. We refer to this as Minimum Acceptable Survival (MAS). Our study is also the first to elicit preferences for the treatment of mBC in the UK; preferences across countries may differ due to cultural factors and different healthcare systems. For example, South-East Asian attitudes to cancer management and death are known to be different to Western ones (17).

Methods

The DCE is a choice-based survey that quantifies preferences for alternatives (e.g. treatment options for mBC) where alternatives are described by their attributes and associated levels (18). In our DCE alternatives are treatments, attributes are treatment characteristics (e.g. survival and side effects), and levels are values associated with treatment characteristics (e.g. % chance of survival, possible levels of severity for nausea).

For peer review only

Defining attributes and levels

Four work packages (WPs) informed the attributes and levels: (i) a targeted literature review of qualitative literature concerning the patient experience of metastatic cancer; (ii) a targeted literature review of DCEs centred on treatments for metastatic cancer; (iii) a thematic analysis (19) of Scottish Medicine's Consortium (SMC) Patient and Clinical Engagement (PACE) statements for mBC treatments; and (iv) face-to-face interviews with mBC patients. For more information on all WPs see Supporting Information 1. The research group, consisting of breast cancer and DCE experts, considered these attributes, reducing them to a manageable number for use in the DCE framework. Attribute selection and layperson definitions were developed using think-aloud interviews with patients (20).

The final attributes and levels are shown in Table 1, with patient definitions of attributes defined in Table A1 in Supporting Information 2. Levels are intended to represent possibilities for first-line treatment following a diagnosis at Stage IV. Side effects were: fatigue; nausea; diarrhoea; and additional side effects (peripheral neuropathy, hand foot syndrome, and mucositis as mutually exclusive levels). Levels of side effects attributes were described using plain-language translations of the Common Terminology Criteria for Adverse Events (CTCAE) (21) criteria (Table A1). These were developed with health professionals and tested in the developmental piloting work. Following piloting with patients, and to ease understanding, fatigue was referred to as tiredness. The nausea attribute combined the corresponding CTCAE grades nausea and vomiting (since they tend to accompany one another). Attribute levels ranged from a zero level of toxicity up to Grade 2. We discussed choice options with health professionals to ensure plausibility. During these discussions it was suggested that some background fatigue is expected for most patients; therefore Grade 1 fatigue was the minimum level of the attribute. It was also advised that in the presence of Grade 3 adverse events, treatment would be discontinued; thus, the maximum level for all adverse event attributes was Grade 2. For additional side effects attribute, all levels were Grade 2. The additional side effects attribute was

For peer review only

included to capture a broader range of side effects while limiting the number of attributes and therefore the cognitive burden of completing the choice tasks (22).

Patient and Public Involvement

Patients with mBC were invited to, and participated in, interviews and in person questionnaire piloting sessions, both of which informed the final design of the survey.

For peer review only

Table 1 Attributes and Levels for the Discrete Choice Experiment

Attributes	Levels	Definition	Regression Equation Label	Regression Equation Preference parameter
Fatigue*	Grade 1 Fatigue (reference level)<br>Grade 2 Fatigue	Tiredness - In this scenario your cancer will always make you more tired than you once were. But treatments can make this worse	G2_FAT	β_1
Nausea	No nausea (reference level)<br>Grade 1 Nausea<br>Grade 2 Nausea	Treatments may cause nausea and nausea may cause you to vomit.	G1_NAU<br>G2_NAU	β_2<br>β_3
Diarrhoea	No diarrhoea (reference level)<br>Grade 1 Diarrhoea<br>Grade 2 Diarrhoea	Treatments may cause diarrhoea.	G1_DIA<br>G2_DIA	β_4<br>β_5
Additional side effects	No other side effects (reference level)<br>Grade 2 Peripheral Neuropathy<br>Grade 2 Hand foot syndrome<br>Grade 2 Mucositis	A treatment may be associated with an additional side effect. These side effects include peripheral neuropathy (nerve damage), hand foot syndrome (severe skin problems), and mucositis (mouth ulcers). You can experience a maximum of one of these side effects on a given treatment.	G2_NEU<br>G2_HAN<br>G2_MUC	β_6<br>β_7<br>β_8
Overall survival	60 alive at 1 year, 8 alive at 5 years<br>65 alive at 1 year, 12 alive at 5 years<br>75 alive at 1 year, 24 alive at 5 years	How long someone lives is always uncertain but in this scenario the care team is able to tell you how many patients are expected to be alive after 1 and 5 years. They are also able to tell you how many of those who survived the first year also experienced an urgent hospital admission. A patient may, for example, have an urgent hospital admission because of a severe infection (sepsis) or because of extreme symptoms. Hospital admission and survival statistics will both be presented in a single graphic. Please imagine that the figure for urgent hospital admissions includes hospital stays which range from days to weeks.	OS	β_9
Risk of urgent hospital admission	1/100 people<br>10/100 people<br>30/100 people		UHA	β_{10}

* Following piloting, and to ease understanding, fatigue was referred to as tiredness.

For peer review only

A risk of urgent hospital admission (UHA) was included, defined as the number of people from 100 treated who would be admitted to the hospital for a UHA. The decision to make UHA a probabilistic attribute was motivated by discussions with health professionals. It was suggested that, unlike Grade 1 and Grade 2 toxicities, a treatment which guaranteed an UHA would not be offered to patients. OS was defined as the annual probability of survival, which was time constant and represented the probability of surviving in the present and future years. To account for short- and long-term preferences (23) annual probability of survival was presented as frequencies at 1 and 5 years e.g. 65% translated to 65 people alive a 1 year and 12 alive at 5 years (the rounded result of 100×0.65^5). The average 1-year survival rate after diagnosis for an mBC patient is approximately 65% (24); we chose this as our central value for our annual survival rate. We used an exponential calculation for 5-year survival, rather than real-world data, to simplify the choice task to include only one risk attribute. The levels for UHA were defined following discussions with health professionals.

It was observed during piloting that some of the expected negative preference for UHA would occur due to a risk of death. Respondents often struggled to disentangle and interpret the related attributes. To isolate the effect independently from the risk of death a graphic was devised, which showed levels of both attributes. The combination of frequencies and tree diagrams has been shown to improve understanding of risks (25, 26). The first row reports the number of patients admitted to the hospital for an UHA, and the second and third show 1- and 5-year survival respectively. Frequencies for positive outcomes (no hospitalisation and survival) and negative outcomes (hospitalisation and death) were both communicated in an attempt to address framing bias (27).

Choices presented to individuals

Ngene (Choice Metrics) was used to create a set of choices from which preferences could be estimated for all possible scenarios; the design was D-efficient, ensuring minimal variation around parameter estimates (28). This resulted in a set of 12 choice tasks. All choices included a no-treatment option, with side effects defined as the least severe level and risk of UHA 0%. To define the

For peer review only

opt-out level of survival respondents were asked what they perceived their chances of survival at 1 and 5 years, resulting in a 45% average level. This was consistently lower than all levels of OS with treatment and judged reasonable given survival at one year among stage 4 breast cancer patients diagnosed in England in 2013 was between 16-43% depending on age, with a mode of 43% (29). The choice context is shown below.

The scenario You are being asked to consider the decision you would make if presented with different metastatic breast cancer treatments. For each question there are only 2 treatment options. If you choose a treatment, the other treatment will not be an option to you in the future. We ask you to imagine that no other treatment options will become available to you in the future. You also have the option to choose to have no treatment. With no treatment you would experience the symptoms of your cancer; your cancer will be left to progress and you will have shorter life expectancy as a result.

The treatments Both treatments are in the form daily pills. Both treatments can treat you for the rest of your life. You would be allowed to stop treatment whenever you wished. Both treatments have different benefits and side effects.

Side effects Side effects are guaranteed. Side effects are already being managed with the best available medicines and care. You will still experience a side effect for weeks at a time.

The choice scenario

Following developmental work, the twelve choices were divided into two blocks of six choice tasks to mitigate mental fatigue effects (30). Respondents were randomly allocated to one of the design blocks and choice tasks were presented in a randomised order. Respondents were given a warm-up choice task (Fig 1) to complete.

ew only

Figure 1: Example of DCE choice task (Warm-up task)

[Fig 1]

Data Analysis

The following utility/benefit function was estimated using multinomial logit (MNL) regression (31):

$$U_{in} = \beta_0 Treat_i + \beta_1 G2\_FAT_{i1} + \beta_2 G1\_NAU_{i2} + \beta_3 G2\_NAU_{i3} + \beta_4 G1\_DIA_{i4} + \beta_5 G2\_DIA_{i5} + \beta_6 G2\_NEU_{i6} + \beta_7 G2\_HAN_{i7} + \beta_8 G2\_MUC_{i8} + \beta_9 OS_{i9} + \beta_{10} UHA_{i10} + \varepsilon_{in}$$

U_{in} represents the utility for individual n for alternative i. β_0 shows the general preference for treatment over no treatment (everything else equal) with a positive sign indicating a general preference to receive treatment (everything else equal). All other variables are defined in Table 1. β_1 to β_8 are modelled as dummy variables, showing the value of that attribute level relative to the reference (best) level. β_9 and β_{10} are modelled as continuous variables, showing the value of a % change in OS and UHA. The signs of the β parameters indicate whether the effect of the attribute level on preference is positive or negative. All side effects preference parameters are expected to have a negative sign relative to the reference level. Respondents are expected to prefer higher OS, resulting in a positive β_9. The preference for chance of UHA, β_{10}, is expected to have a negative sign, with lower values preferred. εin represents the unobserved error component.

We used the parameter values to estimate the relative importance of attributes (32); this is calculated as the difference in the range of attribute's variable values. We calculate percentages from these relative ranges, obtaining a set of attribute importance values that add to 100%. We also estimate MRS in the form of MAS for improvements in side effects using the rate for 1-year OS in the calculation, estimated as $\frac{\beta_x}{-\beta_9}$. For example, $\frac{\beta_1}{-\beta_9}$ shows MAS for a reduction in side effects from Grade 2 fatigue to Grade 1 fatigue and $\frac{\beta_4}{-\beta_9}$ shows MAS for a reduction in side effects from Grade 1 diarrhoea to no diarrhoea.

For peer review only

Sample and Recruitment

Calculating an optimal sample size for newly designed DCEs is problematic as it depends on the true values of the unknown parameters for which the analysis intends to estimate (33). Previous DCEs in the area of metastatic cancer of a similar design have demonstrated that reliable analysis can be performed with samples of 100 or fewer participants (34-36). We therefore aimed to recruit 100 patients as a minimum threshold.

Our target sample was initially women who had experienced metastatic breast cancer. Given the anticipated challenges of recruiting a sufficient number of women who had an mBC diagnosis, we also collected preferences from women who had experienced primary breast cancer. Respondents who responded that they had only a primary breast cancer were asked to imagine that they had received a secondary breast cancer diagnosis in the introductory text. The preferences of metastatic breast cancer patients were compared to primary breast cancer patients.

The DCE was administered using an online link between January and March 2020. Recruitment methods included: (i) distribution of leaflets at cancer centres and conferences; (ii) an online panel provided by Dynata; (iii) social media engagement with help from breast cancer charities; and (iv) a research nurse approaching patients directly during clinic visits and inviting them to complete the survey on a tablet device. Interviewed respondents provided informed written consent before interviews proceeded. Survey respondents self-reported as UK residents over 18 years of age and provided informed consent online at the start of the survey. The protocol was approved by the National Health Service (NHS) North of Scotland Research Ethics Committee (REC ref: 19/NS/0066).

Results

The sample size was 105 (Table A2 in Supporting Information 2). All identified as female. The largest group were mBC patients, 72, followed by primary breast cancer, 33.

10 respondents did not complete all 6 choice tasks, resulting in 29 missing choice tasks. Completed choice tasks were included in the analysis. Of 601 responses to choice tasks across all participants, 38 (6.32%) were for no treatment. These were selected by 16 women, with three women always choosing the opt-out option. 32.38% (N=34) of respondents always chose the option with the highest OS. We focus our analysis on the complete sample as those always choosing the option with the highest survival may have been trading. (Figures A1 and A2 in Supporting Information 3 compares analysis when excluding the 34 potential non-traders; as expected the relative importance of OS is lower and participants have a higher MAS. However, samples are too small to demonstrate statistically significant differences.)

Table 2 shows the MNL regression results for all respondents and Fig 2 shows the relative importance of attributes.

Figure 2: Relative Importance of Attributes

[Figure 2]

Error bars show 95% confidence interval using delta method standard errors

MAS estimates (Table 2, column 8 and Fig 3) show respondents' willingness to forgo OS to avoid all Grade 2 toxicities.

Figure 3: Minimum acceptable survival to Avoid Side effects

[Figure 3]

Error bars show 95% confidence interval using delta method standard errors

Results comparing metastatic breast cancer patients and primary breast cancer patients are shown in Figures A3 and A4 Supporting Information 3. The most notable difference is the estimated

For peer review only

importance of the nausea attribute, nonetheless, there are no statistically significant differences between any of the estimates.

For peer review only

271 **Table 2 Multinomial Logit Results**

		Estimate	p	95% CI Lower bound	95% CI Upper bound	Relative attribute importance	Minimum acceptable survival
Alternative Specific Constant	Treatment	0.9598	0.0006	0.4136	1.5060	-	-
Fatigue	Grade 2 fatigue	-0.2899	0.0089	-0.5073	-0.0726	0.0658	2.8017
Nausea	Grade 1 nausea	-0.3070	0.1021	-0.6750	0.0610	0.0951	2.9665 N.S.
	Grade 2 nausea	-0.4192	0.0232	-0.7811	-0.0573		4.0503
Diarrhoea	Grade 1 diarrhoea	0.0696	0.6425	-0.2242	0.3636	0.1536	-0.6734 N.S.
	Grade 2 diarrhoea	-0.6076	0.0011	-0.9715	-0.2438		5.8714
Additional side effects	Grade 2 peripheral neuropathy	-1.070	0.0000	-1.4654	-0.6748	0.2693	10.3399
	Grade 2 hand foot syndrome	-1.1873	0.0000	-1.5759	-0.7987		11.4723
	Grade 2 mucositis	-1.1264	0.0000	-1.4830	-0.7698		10.8842
Overall survival	Annual probability of survival	0.1035	0.0000	0.0764	0.1305	0.3521	-
Urgent Hospital Admission	Probability of urgent hospital admission in the first year of treatment	0.0097	0.0589	-0.0004	0.0198	0.0640 N.S.	-2.8223 N.S.(for 30% level)
**Model statistics**							
Number of individuals	105						
Observations	601						
Log likelihood	-431.59						
Bayesian info criterion	933.5637						

272 *N.S. not significant*

Discussion

We provide new evidence on UK women's preferences for the treatment of mBC. Respondents had a general preference for treatment, indicated by the low opt-out rates which result in a positive constant term (Treat). As expected, they preferred treatments with higher OS, in fact almost a third of the sample (32.38%) always chose the treatment option with a higher OS. All Grade 2 toxicities were significant and negative, suggesting negative preferences for these attribute levels. However, Grade 1 nausea and diarrhoea were not significant, suggesting patients are indifferent when compared to having none of these side effects. There was no significant effect of UHA on respondents' choices.

The relative importance of OS exceeded all other attributes, with an overall importance score of 35.21%. The remaining relative importance was distributed accordingly: additional side effects (26.93%), diarrhoea (15.26%), nausea (9.51%), fatigue (6.58%), and risk of urgent hospital admission (6.40%). Respondents would accept a reduction in the probability of survival of 2.80%to avoid Grade 2 fatigue (and have Grade 1 fatigue). The MAS associated with levels of the additional side effects were particularly high: respondents were willing to give up 10.34%, 11.47%, and 10.88%chance of OS for total avoidance of grade 2 peripheral neuropathy, grade 2 hand foot syndrome, and grade 2 mucositis respectively. Notably, Grade 1 nausea and diarrhoea were acceptable to patients and did not significantly impact patients' choices. Thus, they were not willing to give up survival for improvements in such Grade 1 side effects. However, Grade 2 side effects were disliked and respondents were willing to forgo up to 11.47% OS to avoid such side effects.

Our results add to a growing literature showing that breast cancer patients value avoiding the side effects of treatments, and are willing to forgo some level of treatment efficacy to achieve this (9,10). Exploring the preferences of women with mBC in the USA, DiBonaventura *et al.* (11) found that whilst OS was the most important attribute, side effects (alopecia, fatigue, neutropenia, motor neuropathy, and nausea/vomiting) and dosing regimen were also important. Omori *et al.* (15) explored the preferences of Japanese postmenopausal patients with HR+ breast cancer for the treatment of

For peer review only

mBC. They conclude that women preferred treatments that extend PFS despite potential grade 2 diarrhoea. However, when diarrhoea severity increased to grade 3, patients were more willing to sacrifice PFS to avoid more frequent diarrhoea. In contrast, exploring preferences of women diagnosed with mBC in Germany, Spaich *et al* (16) concluded that severe neutropenia was the most important attribute, followed by alopecia, neuropathy and PFS. Two studies have explored the preferences of women diagnosed with mBC in the USA, estimating value in monetary terms. Lalla et al (12) found that women were willing to pay the most to avoid severe diarrhoea (US$3,894 a year), followed by avoidance of hospitalization due to infection (US$3,279), severe nausea (US$3,211) and severe peripheral neuropathy (US$2,764). MacEwan et al (13) found that women were willing to pay US$1930 per month for treatment, with US$63 per month for each 1% reduction in the risk of moderate to severe side effects. In a similar study in Thailand, Ngorsuraches and Thongkeaw (14) found respondents were willing to pay US$151.6 per month for every 1 month increase in PFS compared to US$69.8 and US$278.3 per month for every 1% decreased risk of anaemia and pneumonitis respectively.

Our results imply that treatment efficacy and OS are not the only endpoints of value to women with mBC (and indeed oncology more broadly). Furthermore, there is evidence that the CTCAE grading criteria do not scale in parallel with patients' preferences; for example, Grade 2 nausea is preferred to Grade 2 hand foot syndrome (indicated by a lower negative preference parameter). Grade 1 toxicities were not significant, suggesting they are relatively tolerable to patients (compared to having no side effects). These findings suggest that clinician-reported and objectively graded toxicities may not correspond to patients' values and support the further incorporation of Patient Reported Outcomes (PROs) and preference studies in the study of new medicines for mBC. PROs are increasingly accepted by the US Food and Drug Administration (FDA) and European Medicines Agency (EMA) (37) and the National Institute for Health and Care Excellence (NICE) has begun to accept patient preference studies alongside traditional evidence such as cost per QALY (38).

For peer review only

Our study has focused on the preferences of patients. Given that health professionals often make treatment decisions/recommendations for patients, a fruitful area for future research is to compare the preferences of patients and doctors. Current research suggests that it is common for there to be a mismatch in the preferences of patients and healthcare providers (39). Given health professionals possess greater information on treatments and patients possess private information on their values and priorities, Decision Aid Tools (DAT) can help understand and bridge this mismatch as part of shared decision making. The focus of such DATs within breast cancer has been on the detection and prevention of early breast cancer (40). The work presented in this paper contributes to the groundwork for the use of a DCE as a DAT to promote shared decision making and person-centred care. A limited number of studies have adapted DCEs into DATs: Dowsey *et al.* (41) used a DCE as part of a decision aid for patients undergoing total knee arthroplasty; Hazlewood *et al*. (42) evaluated a proof-of-concept DAT for patients with early rheumatoid arthritis, which included a DCE to assist respondents in choosing initial treatment and Loria-Rebolledo *et al*. (43) are exploring the use of DCEs to estimate preferences at the individual level for use in a shared decision making setting.

There are limitations to this study. Firstly, the sample size was small, and we were required to supplement the metastatic breast cancer patient sample with primary breast cancer who were asked to imagine a secondary diagnosis. Although analysis did not present large enough differences in preferences to suggest this meaningfully affected results, a larger sample would allow the possibility of preference heterogeneity to be extensively explored. Preferences, trade-offs and willingness to avoid particular side effects may be influenced by many factors. One potential area for future research is understanding the dynamics of treatment preferences and response shift. This may be particularly important for end-of-life care, which mBC patients may face (44). Other factors that may influence preferences include cancer diagnosis, multiple diagnoses and treatment experience. Future research should explore preference heterogeneity. Secondly, national data indicates that the highest incidence of new breast cancer cases (any stage) for women between 2015 and 2017 was aged 60-69 (45), suggesting our sample is younger with the largest group aged 50-59. Thirdly, our DCE focused on side effects shared by many treatments. They are thus likely to be less applicable for more nuanced

For peer review only

choices that might offer small differences and are associated with side effects we did not investigate e.g., hair loss for chemotherapies or osteonecrosis for bisphosphonate therapies. Fourthly, we investigated willingness to give up OS to avoid particular severity levels. Although this provides insight into patient preferences for side effect severity, our results cannot be used within a benefit-risk trade-off framework, where levels for side effects should be defined as probabilistic. Given the increased interest benefit-risk by policymakers such as the FDA (46), future work could extend this survey to incorporate the probability of side effects. Given the known difficulties of understanding the risk of side effects, attention should be given to respondents' understanding of the survey. Fifthly, to simplify the choice task to include only one risk attribute, we used an exponential function to estimate the five-year survival rate. Future research could include two attributes, one and five-year survival, with the latter based on real data. Preferences for short and long-term survival could then be estimated. Next, in defining the no treatment option, the level for OS was defined as the mean value from women's perceived OS without treatment. Results may have differed if we told women their chance of survival without treatment. Finally, whilst the insignificance of the risk of UHA may be a genuine preference, the result may also reflect a difficulty in understanding this attribute. Despite low relative importance, similar attributes are significant in other metastatic cancer DCEs, however, the attribute levels are more severe (47, 48). Future work should explore explaining this attribute.

In conclusion, our results provide evidence that patients are willing to give up some survival benefits to avoid severe levels of side effects. Future therapeutic studies should ensure such data is collected to ensure that the patient can make an informed decision when making treatment decisions. Future research should explore using such information within a shared decision-making framework.

For peer review only

Data Sharing

The dataset used for this analysis is available from the University of Edinburgh Datashare, https://datashare.ed.ac.uk/handle/10283/4436.

Funding

This research was supported by a charitable grant from the Edinburgh and Lothians Health Foundation (Scottish Registered Charity No: SC007342). Mandy Ryan and Luis E Loría-Rebolledo are funded by the University of Aberdeen and the Chief Scientist Office of the Scottish Government Health and Social Care Directorates. The funders had no role in considering the study design or in the collection, analysis, or interpretation of data, the writing of the paper, or the decision to submit the article for publication.

Author Contributions

PH, EG, HE and MR contributed to the conceptualisation of the project and funding application. All authors contributed to the design of the study. AB developed the DCE, with experimental design support from MR. AB and MM collected the data. AB conducted the statistical analysis with support from LL. All authors contributed to the interpretation of the data as well as the drafting and revision of the manuscript. All authors gave final approval and agreed to be accountable for all aspects of the work.

Acknowledgements

We thank all the patients and volunteers who agreed to participate in this study and the study contributors.

For peer review only

References

1) Feinmann J. Number of UK patients with metastatic breast cancer isn't known. 2017

2) Brom L, Pasman HR, Widdershoven GA, van der Vorst MJ, Reijneveld JC, Postma TJ, Onwuteaka-Philipsen BD. Patients' preferences for participation in treatment decision-making at the end of life: qualitative interviews with advanced cancer patients. PloS one. 2014 Jun 25;9(6):e100435.

3) Waks AG, Winer EP. Breast cancer treatment: a review. Jama. 2019 Jan 22;321(3):288-300.

4) Mosher CE, Johnson C, Dickler M, Norton L, Massie MJ, DuHamel K. Living with metastatic breast cancer: a qualitative analysis of physical, psychological, and social sequelae. The breast journal. 2013 May;19(3):285-92.

5) Scottish Government. What Works to Support and Promote Shared Decision Making: A synthesis of recent evidence. 2019. Available from: https://www.gov.scot/publications/works-support-promote-shared-decision-making-synthesis-recent-evidence/pages/3/. Last accessed 7th March 2022.

6) NICE. Shared Decision Making Collaborative – An Action Plan. 2016. Available from: https://www.nice.org.uk/Media/Default/About/what-we-do/shared-decision-making-collaborative-action-plan.pdf. Last accessed 7th March 2022.

7) Covvey JR, Kamal KM, Gorse EE, Mehta Z, Dhumal T, Heidari E, Rao D, Zacker C. Barriers and facilitators to shared decision-making in oncology: a systematic review of the literature. Supportive Care in Cancer. 2019 May;27(5):1613-37.

8) Soekhai V, de Bekker-Grob EW, Ellis AR, Vass CM. Discrete choice experiments in health economics: past, present and future. Pharmacoeconomics. 2019 Feb;37(2):201-26.

For peer review only

422 9) Collacott H, Soekhai V, Thomas C, Brooks A, Brookes E, Lo R, Mulnick S, Heidenreich S. A systematic review of discrete choice experiments in oncology treatments. The Patient-Patient-Centered Outcomes Research. 2021 Nov;14(6):775-90.

425 10) Guerra RL, Castaneda L, de Albuquerque RD, Ferreira CB, Correa FD, Fernandes RR, de Almeida LM. Patient preferences for breast cancer treatment interventions: a systematic review of discrete choice experiments. The Patient-Patient-Centered Outcomes Research. 2019 Dec;12(6):559-69.

429 11) DiBonaventura M, Copher R, Basurto E, Faria C, Lorenzo R. Patient preferences and treatment adherence among women diagnosed with metastatic breast cancer. American Health & Drug Benefits. 2014 Oct;7(7):386.

432 12) Lalla D, Carlton R, Santos E, Bramley T, D'Souza A. Willingness to pay to avoid metastatic breast cancer treatment side effects: results from a conjoint analysis. Springerplus. 2014 Dec;3(1):1-7.

435 13) MacEwan JP, Doctor J, Mulligan K, May SG, Batt K, Zacker C, Lakdawalla D, Goldman D. The value of progression-free survival in metastatic breast cancer: results from a survey of patients and providers. MDM policy & practice. 2019 Jun;4(1):2381468319855386.

438 14) Ngorsuraches S, Thongkeaw K. Patients' preferences and willingness-to-pay for postmenopausal hormone receptor-positive, HER2-negative advanced breast cancer treatments after failure of standard treatments. Springerplus. 2015 Dec;4(1):1-1.

441 15) Omori Y, Enatsu S, Cai Z, Ishiguro H. Patients' preferences for postmenopausal hormone receptor-positive, human epidermal growth factor receptor 2-negative advanced breast cancer treatments in Japan. Breast Cancer. 2019 Sep;26(5):652-62.

For peer review only

16) Spaich S, Kinder J, Hetjens S, Fuxius S, Gerhardt A, Sütterlin M. Patient preferences regarding chemotherapy in metastatic breast cancer—a conjoint analysis for common taxanes. Frontiers in oncology. 2018 Nov 21;8:535.

17) Ong KJ, Back MF, Lu JJ, Shakespeare TS, Wynne CJ. Cultural attitudes to cancer management in traditional South-East Asian patients. Australasian radiology. 2002 Dec;46(4):370-4.

18) Lancaster KJ. A new approach to consumer theory. Journal of political economy. 1966 Apr 1;74(2):132-57.

19) Braun V, Clarke V. Thematic analysis. 2012

20) Cheraghi-Sohi S, Bower P, Mead N, McDonald R, Whalley D, Roland M. Making sense of patient priorities: applying discrete choice methods in primary care using 'think aloud' technique. Family practice. 2007 Jun 1;24(3):276-82.

21) National Cancer Institute (U.S.). Adverse events/CTCAE. 2021. Available: https://ctep.cancer.gov/protocoldevelopment/electronic_applications/ctc.htm. Last accessed 7th March 2022.

22) Bridges JF, Hauber AB, Marshall D, Lloyd A, Prosser LA, Regier DA, Johnson FR, Mauskopf J. Conjoint analysis applications in health—a checklist: a report of the ISPOR Good Research Practices for Conjoint Analysis Task Force. Value in health. 2011 Jun 1;14(4):403-13.

23) Chao LW, Szrek H, Pereira NS, Pauly MV. Time preference and its relationship with age, health, and survival probability. Judgment and decision making. 2009 Feb 1;4(1):1.

24) Cancer Research UK. Breast survival by stage at diagnosis. Breast cancer survival statistics. 2019. Available from: https://www.cancerresearchuk.org/health-professional/cancer-

For peer review only

statistics/statistics-by-cancer-type/breast-cancer/survival#heading-Three. Last accessed 11th November 2022.

25) Kurz-Milcke E, Gigerenzer G, Martignon L. Transparency in risk communication: graphical and analog tools. Annals of the New York Academy of Sciences. 2008 Apr;1128(1):18-28.

26) Spiegelhalter D, Pearson M, Short I. Visualizing uncertainty about the future. science. 2011 Sep 9;333(6048):1393-400.

27) Howard K, Salkeld G. Does attribute framing in discrete choice experiments influence willingness to pay? Results from a discrete choice experiment in screening for colorectal cancer. Value in health. 2009 Mar;12(2):354-63.

28) ChoiceMetrics.NGENE (1.2). 2018. [NGENE]. [Accessed 20 January 2020].

29) Office for National Statistics. Cancer survival in England: adult, stage at diagnosis and childhood - patients followed up to 2018. 2019. Available from: https://www.ons.gov.uk/peoplepopulationandcommunity/healthandsocialcare/conditionsanddiseases/bulletins/cancersurvivalinengland/stageatdiagnosisandchildhoodpatientsfollowedupto2018. Last accessed 21st January 2021.

30) Bech M, Kjaer T, Lauridsen J. Does the number of choice sets matter? Results from a web survey applying a discrete choice experiment. Health economics. 2011 Mar;20(3):273-86.

31) McFadden D. The measurement of urban travel demand. Journal of public economics. 1974 Nov 1;3(4):303-28.

32) Hauber AB, González JM, Groothuis-Oudshoorn CG, Prior T, Marshall DA, Cunningham C, IJzerman MJ, Bridges JF. Statistical methods for the analysis of discrete choice experiments: a report of the ISPOR conjoint analysis good research practices task force. Value in health. 2016 Jun 1;19(4):300-15.

For peer review only

33) Lancsar E, Louviere J. Conducting discrete choice experiments to inform healthcare decision making: a user's guide. Pharmacoeconomics. 2008 Aug;26:661-77.

34) Bridges, J.F., Mohamed, A.F., Finnern, H.W., Woehl, A. and Hauber, A.B., 2012. Patients' preferences for treatment outcomes for advanced non-small cell lung cancer: a conjoint analysis. Lung Cancer, 77(1), pp.224-231.

35) Hofheinz, R., Clouth, J., Borchardt-Wagner, J., Wagner, U., Weidling, E., Jen, M.H. and Brück, P., 2016. Patient preferences for palliative treatment of locally advanced or metastatic gastric cancer and adenocarcinoma of the gastroesophageal junction: a choice-based conjoint analysis study from Germany. *BMC cancer*, *16*, pp.1-9.

36) Ivanova, J., Hess, L.M., Garcia-Horton, V., Graham, S., Liu, X., Zhu, Y. and Nicol, S., 2019. Patient and oncologist preferences for the treatment of adults with advanced soft tissue sarcoma: a discrete choice experiment. *The Patient-Patient-Centered Outcomes Research*, *12*, pp.393-404.

37) Bottomley A, Jones D, Claassens L. Patient-reported outcomes: assessment and current perspectives of the guidelines of the Food and Drug Administration and the reflection paper of the European Medicines Agency. European Journal of Cancer. 2009 Feb 1;45(3):347-53.

38) Bouvy JC, Cowie L, Lovett R, Morrison D, Livingstone H, Crabb N. Use of patient preference studies in HTA decision making: a NICE perspective. The Patient-Patient-Centered Outcomes Research. 2020 Apr;13(2):145-9.

39) Harrison M, Milbers K, Hudson M, Bansback N. Do patients and health care providers have discordant preferences about which aspects of treatments matter most? Evidence from a systematic review of discrete choice experiments. BMJ open. 2017 May 1;7(5):e014719.

40) Gao JP, Jin YH, Yu SF, Wu WF, Han SF. Evaluate the effectiveness of breast cancer decision aids: A systematic review and meta-analysis of randomize clinical trails. Nursing Open. 2021 Sep;8(5):2091-104.

For peer review only

515 41) Dowsey MM, Scott A, Nelson EA, Li J, Sundararajan V, Nikpour M, Choong PF. Using discrete choice experiments as a decision aid in total knee arthroplasty: study protocol for a randomised controlled trial. Trials. 2016 Dec;17(1):1-0.

518 42) Hazlewood GS, Marshall DA, Barber CE, Li LC, Barnabe C, Bykerk V, Tugwell P, Hull PM, Bansback N. Using a discrete-choice experiment in a decision aid to nudge patients towards value-concordant treatment choices in rheumatoid arthritis: a proof-of-concept study. Patient preference and adherence. 2020;14:829.

522 43) Loría-Rebolledo LE, Ryan M, Bond C, Porteous T, Murchie P, Adam R. Using a discrete choice experiment to develop a decision aid tool to inform the management of persistent pain in pharmacy: a protocol for a randomised feasibility study. BMJ open. 2022 Sep 1;12(9):e066379.

526 44) Schwartz CE, Merriman MP, Reed GW, Hammes BJ. Measuring patient treatment preferences in end-of-life care research: applications for advance care planning interventions and response shift research. Journal of Palliative Medicine. 2004 Apr 1;7(2):233-45.

529 45) Cancer Research UK. Breast cancer incidence by age. Breast cancer incidence (invasive) statistics. 2021. Available from: https://www.cancerresearchuk.org/health-professional/cancer-statistics/statistics-by-cancer-type/breast-cancer/incidence-invasive#heading-One. Last accessed 11th November 2022.

533 46) Vass CM, Payne K. Using discrete choice experiments to inform the benefit-risk assessment of medicines: are we ready yet?. Pharmacoeconomics. 2017 Sep;35(9):859-66.

535 47) Bröckelmann PJ, McMullen S, Wilson JB, Mueller K, Goring S, Stamatoullas A, Zagadailov E, Gautam A, Huebner D, Dalal M, Illidge T. Patient and physician preferences for first-line treatment of classical Hodgkin lymphoma in Germany, France and the United Kingdom. British Journal of Haematology. 2019 Jan;184(2):202-14.

For peer review only

48) Ivanova J, Hess LM, Garcia-Horton V, Graham S, Liu X, Zhu Y, Nicol S. Patient and oncologist preferences for the treatment of adults with advanced soft tissue sarcoma: a discrete choice experiment. The Patient-Patient-Centered Outcomes Research. 2019 Aug;12(4):393-404.

For peer review only

For peer review only - http://bmjopen.bmj.com/site/about/guidelines.xhtml

Supporting Information

S1 File. Qualitative Methods

S2 File. Additional Tables

S3 File. Additional Figures

For peer review only

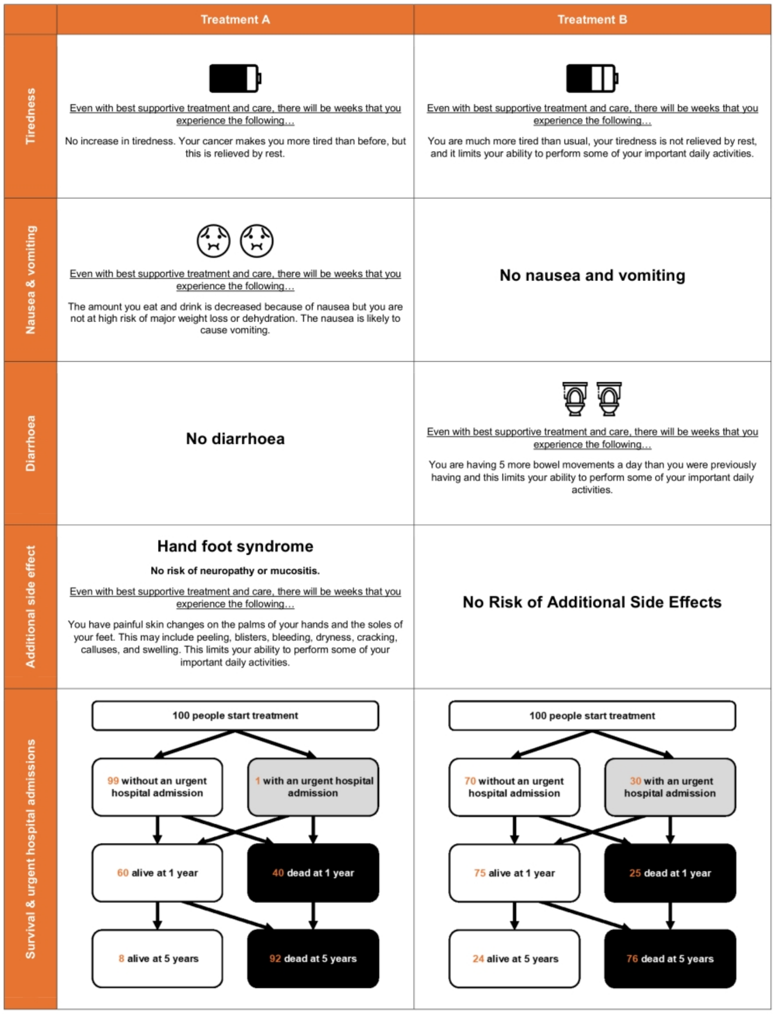

Responses for Patients:

Which option would you choose?

- ○ I would choose to take treatment A
- ○ I would choose to take treatment B
- ○ I would choose neither. I understand that I would have worse expected survival as a result.

Figure 1: Example of DCE choice task (Warm-up task)

296x419mm (200 x 200 DPI)

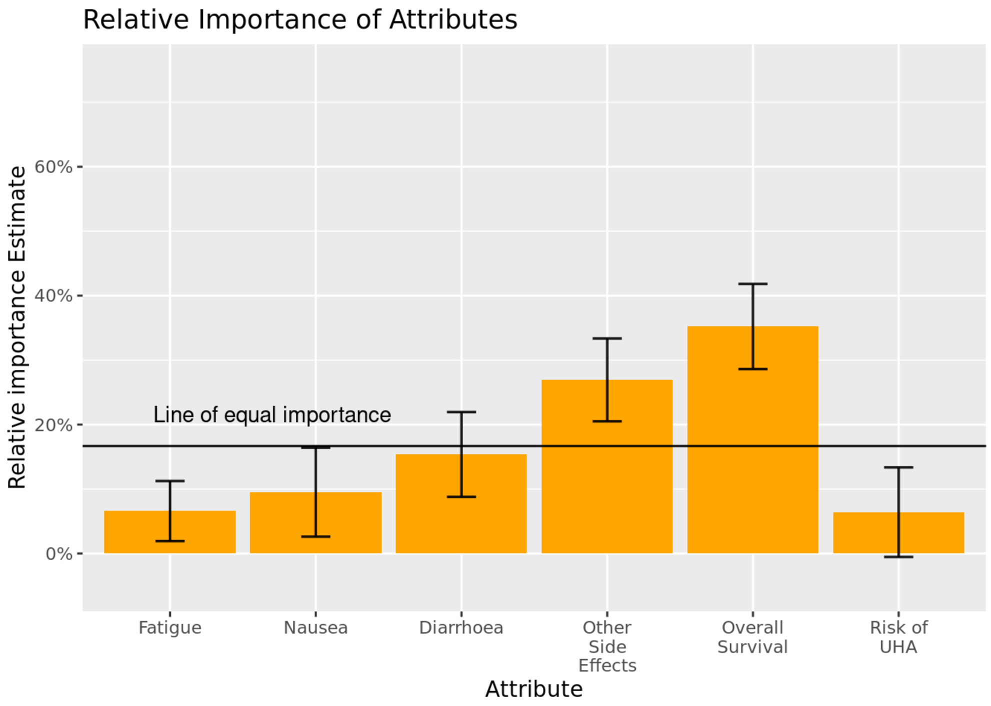

Figure 2: Relative Importance of Attributes

449x320mm (76 x 76 DPI)

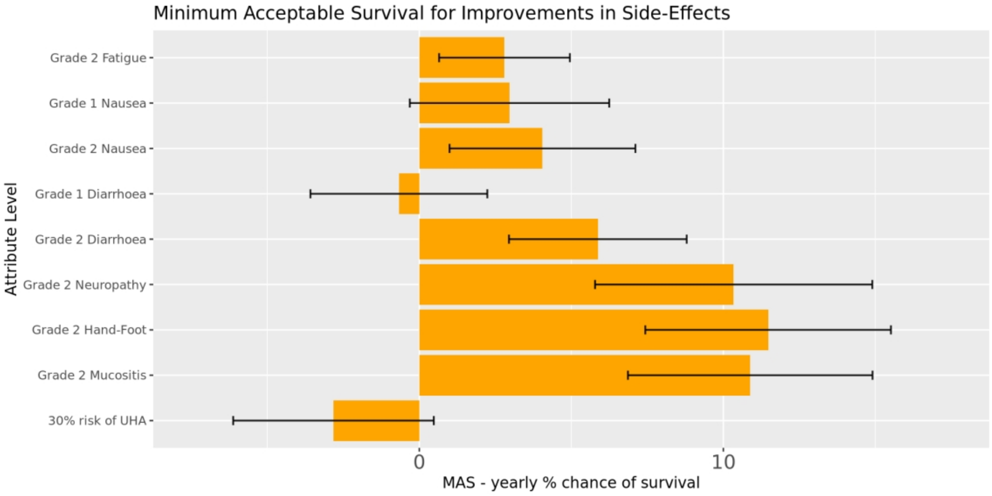

Figure 3: Minimum acceptable survival to Avoid Side effects

641x320mm (76 x 76 DPI)

Women's preferences for overall survival versus avoiding side effects in the treatment of metastatic breast cancer: a discrete choice experiment

Supporting Information 1

Qualitative Methods

Qualitative Literature Review

Embase and Medline were searched using the Ovid search engine. We aimed to identify literature which explored the patient perspective of cancer and the associated treatments. Search terms were designed to identify studies that (1) involved interviews/focus groups (2) explored patient attitudes/perspectives (3) focused on advanced or locally advanced cancer. We included all metastatic cancers given the scarcity of metastatic breast cancer-specific literature.

The search identified 434 results. Abstracts were screened and papers were excluded if they didn't reflect the underlying motivation of the search strategy. Studies were also excluded if: they focussed on an intervention which was not clinically supported or was not medicine (e.g., alternative medicine and exercise respectively); the study focus was seldom relevant to breast cancer (e.g., breathing complications brought on lung tumours). After abstract screening 83 studies remained after which 5 additional studies were excluded after reading beyond the abstract. The remaining papers were evaluated and findings which offered insight into determinants of a patient's quality of life or preference for treatment were identified. Findings were compiled and condensed into a report summarising what the available research to date suggested determining patient preferences and well-being.

Pain was among the most prominent topics of discussion. Respondents who had experienced cancer pain identified it as the most disturbing and limiting symptom of their illness (Luoma and Hakamies-Blomqvist, 2004). Patients with pain often reported extreme negative emotions (Lewis et al, 2015), loss of independence (Gibbins et al, 2014), and a desire for assisted death (Koffman et al, 2008). Other frequently explored topics included physical functioning and mobility which, as concepts, are closely linked to pain (Wilson et al, 2005). The symptoms of disease and the side effects of treatment which led to degraded physical functioning levels were identified as substantial barriers to a patient's ability to live a normal life (Gibbins et al, 2014). Extreme degradation of mobility leads to increased dependence on loved ones and carers which can create a strong sense of burden (Mak, and Elwyn, 2005). Cognitive functioning also appears to have been a topic of interest for qualitative researchers. Although cognitive functioning appears to have been a significant area of interest, many metastatic breast cancer patients rarely had symptoms, when they did, they presented as secondary disturbances or anxieties (Luoma and Hakamies-Blomqvist, 2004). Patients were willing to take medications which were associated with drowsiness to alleviate symptoms of pain (Check et al, 2017). This is evidence that patients already accept trade-offs between symptoms when considering treatments. Evidence of similar trade-offs was also found between: hot flushes and mode of administration (Fallowfield et al, 2005), expected survival and physical functioning (Check et al, 2017), and expected survival against the collective side effects of chemotherapy (Etkind et al, 2017). Evidence of trade-offs between symptoms and side effects tells us something about the importance of those toxicities, but more importantly, helps to validate the decisional context we use to frame our DCE survey questions. Other themes which featured heavily in the literature were the topics of survival, fatigue, and mode of administration, all of which are discussed in more detail in section 4 of this paper.

For peer review only

DCE Literature Review

The benefits of reviewing DCEs with similar motivations to our study are twofold. Firstly, they can offer insight into the importance of some of the treatment factors which we would be considering. Secondly, DCEs often employ rigorous qualitative processes and their choice of attributes is likely to be of interest because their selection implicitly suggests significance. In the context of a cancer treatment DCE an attribute would be a feature of treatment which has the potential to vary between competing hypothetical treatments in a choice task. Embase and Medline were searched for DCE studies relating to patient preference for metastatic cancer treatments[1]. Search terms designed to identify DCEs mirrored those first used by Ryan and Gerard (2003). We also reincorporated the search terms used to identify metastatic cancer studies used in the qualitative literature review. Once again preliminary searches revealed that there was an insufficient body of publications to focus on metastatic breast cancer studies alone. 128 unique studies were identified in total. After screening the abstracts 60 papers met the eligibility criteria. There were 16 instances where two studies reported the results from the same DCE, in these instances the most recent publication was selected. 44 studies were identified as meeting all the criteria. Once the papers were identified work began to analyse the attributes used by the studies. The WP produced 2 key outputs of interest (1) an outline of the types of attributes used in similar past DCEs and (2) their relative importance.

Attributes were grouped into categories with similar motives. The table below outlines the attribute categories which featured in more than one DCE. There were instances where one DCE contained more than one attribute which could fit into the same category, in which instance only one was counted.

Table 1 Frequency of attribute categories included in the DCE literature review

**Attribute Category Frequency**	**n**
Administration	12
Progression Free Survival	12
Cost	8
Overall Survival	8
Pain	7
Fatigue	5
Gastrointestinal Perforation	3
Kidneys	3
Skin	3
Teeth/jaw	3
Adverse Events	2
Bone Metastases	2
Diarrhoea	2
Hospitalisation	2
Immunosuppression	2
Nausea	2
Neuropathy	2
Response rates	2
Self-care	2

[1] the number metastatic breast cancer specific studies identified in preliminary searches were insufficient to justify their own review

For peer review only

Relative preference weights are measures of the importance of attributes relative to competing attributes and are conditional on the range of utility estimates for the remaining attributes (Hauber et al, 2016). A large relative preference weight suggests that an attribute has high importance in the context of the DCE's design. The selection of competing attributes, the range of levels for the attribute and its competitors, and framing effects (Howard and Salkeld, 2009) all determine the scale of a relative preference weight. Nevertheless, underlying preference is still a key determinant of relative preference weights and, if the considerations are accounted for, valuable inferences are possible. When making comparisons between DCEs differing study designs should be considered including decisional context, the motivations of the studies, statistical methods, and sample compositions. The complexity of these comparisons means they can't be definitively relied upon, nonetheless they are useful when consolidated with additional information from other WPs.

The main finding of the DCE literature review was the prevalence of certain attributes among the DCEs, furthermore, certain attributes tended to be associated with high relative importance between DCEs. The closely related attributes of progression free survival (PFS) and overall survival (OS) were both frequently included and tended to have high relative importance, the significance of survival and the relationship between these variations will be explored in more depth in section 4 of this paper. Pain was another category of attribute which was frequently explored and tended to be associated with high relative importance, this suggests a strong preference amongst patients to minimise suffering. It is also worth noting that, many studies appeared to be interested in patients' preferences for mode of administration, although it appeared respondents often prioritised other attributes. As a final note, the relative importance of many symptoms and side effects such as fatigue, nausea and diarrhoea differed greatly between DCEs, it was here that the limitations of making deductions from the results DCEs with different objectives were most apparent.

PACE Statement Thematic Analysis

We were granted access by the Scottish Medicines Consortium (SMC) to eight PACE statements relating to metastatic breast cancer treatments. The SMC is Scotland's advisory body for medicines, as part of their drug approval process for ultra-orphan and end-of-life medicines they invite patient and clinical representatives to meetings to discuss the benefits. These are known as Patient and Clinical Engagement (PACE) meetings. PACE meetings aim to consider all available and relevant evidence regarding new medicines including factors which traditional economic evaluation tends to overlook. We identified PACE statements as a potentially useful secondary data resource for our research since their focus is on the needs of the patient. Another advantage is that PACE statements are a relatively recent innovation meaning they tend to present up-to-date information. Between Oct 2014 and Oct 2018, eight PACE meetings were convened for medicines seeking reimbursement for the treatment of metastatic breast cancer. We conducted a formal thematic analysis (Braun and Clarke, 2012) of the PACE statements which focussed on the positive and negative impacts of treatment as well the insights into patient priorities.

We were able to identify six core themes which were composed of additional sub-themes (see figure below). Themes were not mutually exclusive, meaning there is some degree of overlap between themes. Two of the themes represent what we came to understand as the core goals of patients according to the data, these were 'Ability to live a normal life' and 'Survival'; treatments were praised repeatedly by committees for their ability to improve these two outcomes. When consulting the evidence from the PACE analysis it should be considered that they are designed to consider externalities and not just the direct effect on patients. Specifically, PACE guidelines request that respondents discuss the effect of disease and treatment on the family and carers. This explains the prominence of the 'effect on close ones' theme which is often featured in the form of considering perspectives outside of the patients. Although the findings were interesting for our research, we

For peer review only

decided to focus on the perspective of the patient. So naturally, this theme emerged. A key disadvantage of PACE statements was their tendency to talk broadly and generally about symptoms and side effects. For our research, we were interested in patients' preferences for specific symptoms and side effects, but the lack of detail meant little could be deduced about which common side effects were more troublesome than others. It should also be noted that PACE statements are rarely critical of emerging drugs. The general feeling from the PACE statements was that participants were keen to highlight the benefits of emerging drugs. There was a positive bias that we had to consider when toxicities and benefits associated with the treatment in question were mentioned

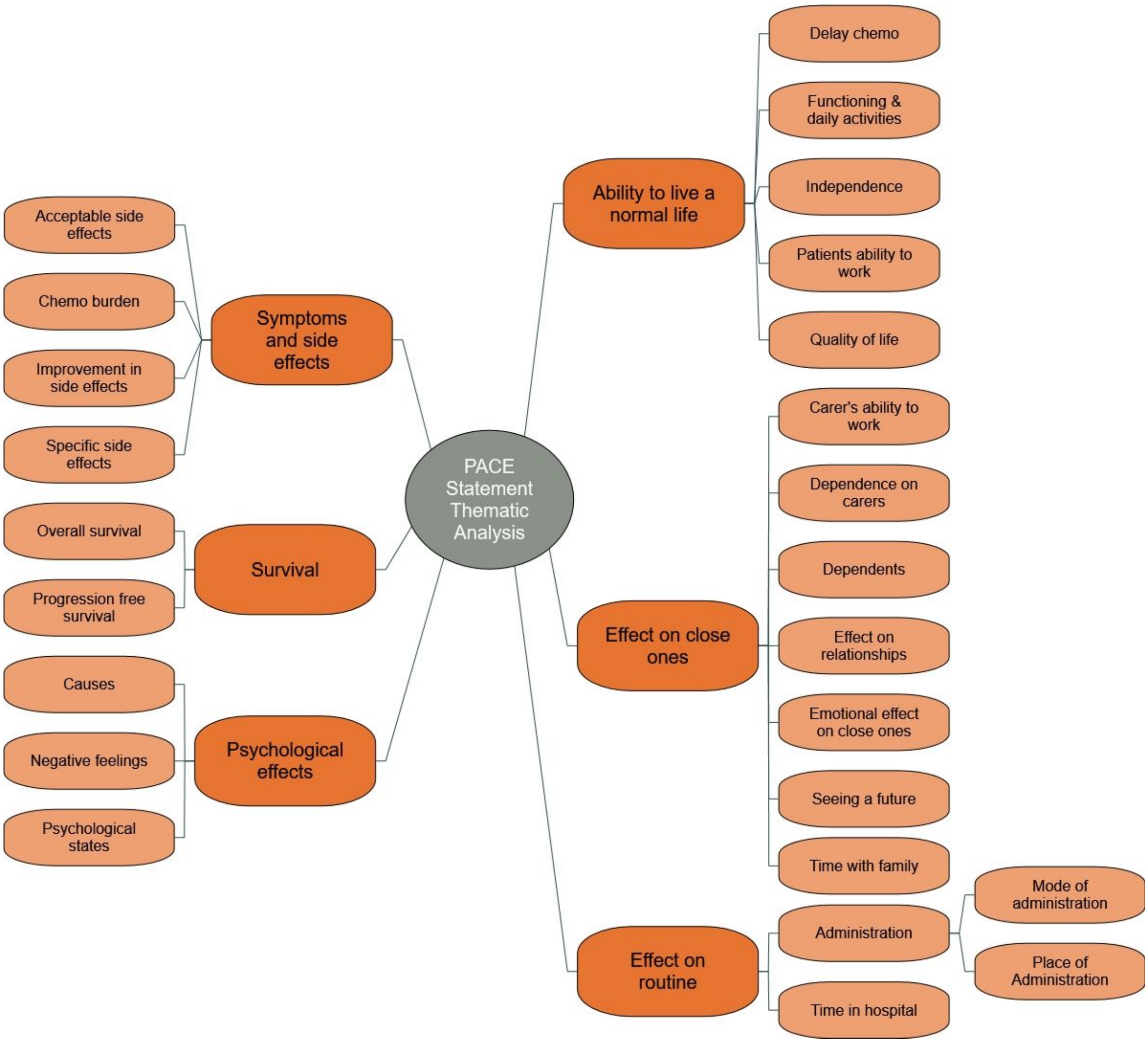

Figure 1 – Results from thematic analysis of breast cancer PACE data

Patient Interviews

The richest data from the early stages of the project emerged from the semi-structured interviews we conducted with 9 patients diagnosed with metastatic breast cancer. Women with secondary breast cases with experience of multiple treatments and who were currently living in the Lothian area were contacted by a research nurse and invited to participate in a face-to-face interview at an agreed location, either a cancer charity premises or the patient's home. We wanted to adopt a flexible strategy where we could adapt individual interviews and our broader strategies as our understanding of patient preferences and experiences developed. Grounded theory (Strauss and Corbin, 1994) is a

qualitative methodology that encourages a flexible strategy, however, conventional recommendations state that interviewers should be mostly ignorant about the topic being explored so that bias does not interfere with the formulation of theories. Given that we already had considerable knowledge of the experiences of breast cancer patients, owing to ongoing research and professional experience, we instead opted to conduct interviews according to the informed grounded theory approach (Thornberg, 2012). This adaptation of the grounded theory methodology allowed us to incorporate our prior knowledge in the traditional grounded theory approach whilst being aware of bias and remaining open to new ideas. An interview plan was formulated which provided structure whilst allowing for deviation and elaboration. The three core areas of focus were patient history, treatment decision making, and experience with treatment and disease.

To summarise the broader findings: There was a general attitude that more treatment was generally better and that listening to the advice of health professionals is the best thing one can do. There was a large degree of variation in terms of the specific side effects that patients' experiences and to what extent. This is likely a consequence of the wide range of secondary malignancies and the treatments received. Several patients mentioned suffering very little from symptoms and side effects since their secondary diagnosis. There was a prevailing negative attitude towards chemotherapy and its associated toxicities. The two primary goals of treatment appeared to be life extension and minimising disruption to everyday life. The interviews helped us to understand the broader goals of patients as well as their self-reported attitudes and behaviours regarding shared decision making. The richest findings however related to discussions concerning specific symptoms and side effects, evidence from these discussions will feature heavily in section 4 of this paper.

For peer review only

References

Braun, V. and Clarke, V., 2012. Thematic analysis.

Check, D. K., Park, E. M., Reeder-Hayes, K. E., Mayer, D. K., Deal, A. M., Yopp, J. M., … Hanson, L. C. (2017). Concerns underlying treatment preferences of advanced cancer patients with children. Psycho-Oncology, 26(10), 1491–1497.

Etkind, S.N., Bristowe, K., Bailey, K., Selman, L.E. and Murtagh, F.E., 2017. How does uncertainty shape patient experience in advanced illness? A secondary analysis of qualitative data. Palliative medicine, 31(2), pp.171-180.

Gibbins, J., Bhatia, R., Forbes, K., & Reid, C. M. (2014). What do patients with advanced incurable cancer want from the management of their pain? A qualitative study. Palliative Medicine, 28(1), 71–78.

Hauber, A.B., González, J.M., Groothuis-Oudshoorn, C.G., Prior, T., Marshall, D.A., Cunningham, C., IJzerman, M.J. and Bridges, J.F., 2016. Statistical methods for the analysis of discrete choice experiments: a report of the ISPOR Conjoint Analysis Good Research Practices Task Force. Value in health, 19(4), pp.300-315.

Howard, K. and Salkeld, G., 2009. Does attribute framing in discrete choice experiments influence willingness to pay? Results from a discrete choice experiment in screening for colorectal cancer. Value in health, 12(2), pp.354-363.

Koffman, J., Morgan, M., Edmonds, P., Speck, P., & Higginson, I. (2008). Cultural meanings of pain: A qualitative study of Black Caribbean and White British patients with advanced cancer. Palliative Medicine, 22(4), 350–359.

Lewis, S., Yee, J., Kilbreath, S. and Willis, K., 2015. A qualitative study of women's experiences of healthcare, treatment and support for metastatic breast cancer. The Breast, 24(3), pp.242-247.

Luoma, M.L. and Hakamies-Blomqvist, L., 2004. The meaning of quality of life in patients being treated for advanced breast cancer: a qualitative study. Psycho-Oncology: Journal of the Psychological, Social and Behavioral Dimensions of Cancer, 13(10), pp.729-739.

Mak, Y.Y.W. and Elwyn, G., 2005. Voices of the terminally ill: uncovering the meaning of desire for euthanasia. Palliative medicine, 19(4), pp.343-350.

Ryan, M. and Gerard, K., 2003. Using discrete choice experiments to value health care programmes: current practice and. Applied health economics and health policy, 2(1), pp.55-64.Hauber, A.B.,

Strauss, A. and Corbin, J., 1994. Grounded theory methodology. Handbook of qualitative research, 17, pp.273-85.

Thornberg, R., 2012. Informed grounded theory. Scandinavian Journal of Educational Research, 56(3), pp.243-259.

Wilson, K. G., Curran, D., & McPherson, C. J. (2005). A burden to others: A common source of distress for the terminally ill. Cognitive Behaviour Therapy, 34(2), 115–123

For peer review only

Supporting Information 2

Table A1 Presentation of Side Effects Attribute Levels to Respondents

**Tiredness**	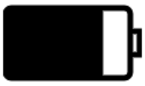<br>Even with best supportive treatment and care, there will be weeks that you experience the following…<br>No increase in tiredness. Your cancer makes you more tired than before, but this is relieved by rest.	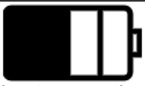<br>Even with best supportive treatment and care, there will be weeks that you experience the following…<br>You are much more tired than usual, your tiredness is not relieved by rest, and it limits your ability to perform some of your important daily activities.	
**Nausea and Vomiting**	**No nausea and vomiting**	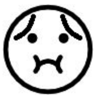<br>Even with best supportive treatment and care, there will be weeks that you experience the following…<br>You have lost your appetite due to nausea, but not enough to change the amount you eat. Your nausea may cause some vomiting.	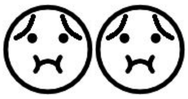<br>Even with best supportive treatment and care, there will be weeks that you experience the following…<br>The amount you eat and drink is decreased because of nausea but you are not at high risk of major weight loss or dehydration. The nausea is likely to cause vomiting.
**Diarrhoea**	**No diarrhoea**	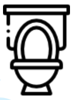<br>Even with best supportive treatment and care, there will be weeks that you experience the following…<br>You are having 2 more bowel movements a day than you were previously having.	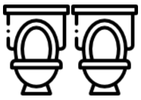<br>Even with best supportive treatment and care, there will be weeks that you experience the following…<br>You are having 5 more bowel movements a day than you were previously having and this limits your ability to perform some of your important daily activities.
**Additional Side effect**	**Peripheral neuropathy**<br>**No risk of hand foot syndrome or mucositis.**<br>Even with best supportive treatment and care, there will be weeks that you experience the following…<br>You have numbness and tingling in the feet or hands and occasionally burning, stabbing or shooting pain in affected areas. This limits your ability to perform some of your important daily activities.	**Hand foot syndrome**<br>**No risk of neuropathy or mucositis.**<br>Even with best supportive treatment and care, there will be weeks that you experience the following…<br>You have painful skin changes on the palms of your hands and the soles of your feet. This may include peeling, blisters, bleeding, dryness, cracking, calluses, and swelling. This limits your ability to perform some of your important daily activities.	**Mucositis**<br>**No risk of neuropathy or hand foot syndrome.**<br>Even with best supportive treatment and care, there will be weeks that you experience the following…<br>Your mouth becomes sore and inflamed. You have ulcers which are painful and mean you are unable to eat spicy, acidic, and crunchy foods such as crisps.
	**No risk of neuropathy, hand foot syndrome or mucositis**		

Table A2 Respondent characteristics

**Diagnosis**	**n**
Metastatic breast cancer	72
Primary breast cancer	33
**Gender**	
Female	105
Male	0
**Age**	
30-39	8
40-49	19
50-59	47
60-69	25
70-79	6

For peer review only

Women's preferences for overall survival versus avoiding side effects in the treatment of metastatic breast cancer: a discrete choice experiment

Supporting Information 3

Figure A1 Comparison of Relative importance Estimates between the entire sample and 'survival non-traders' (Sample excluding survival non-traders, N=71)

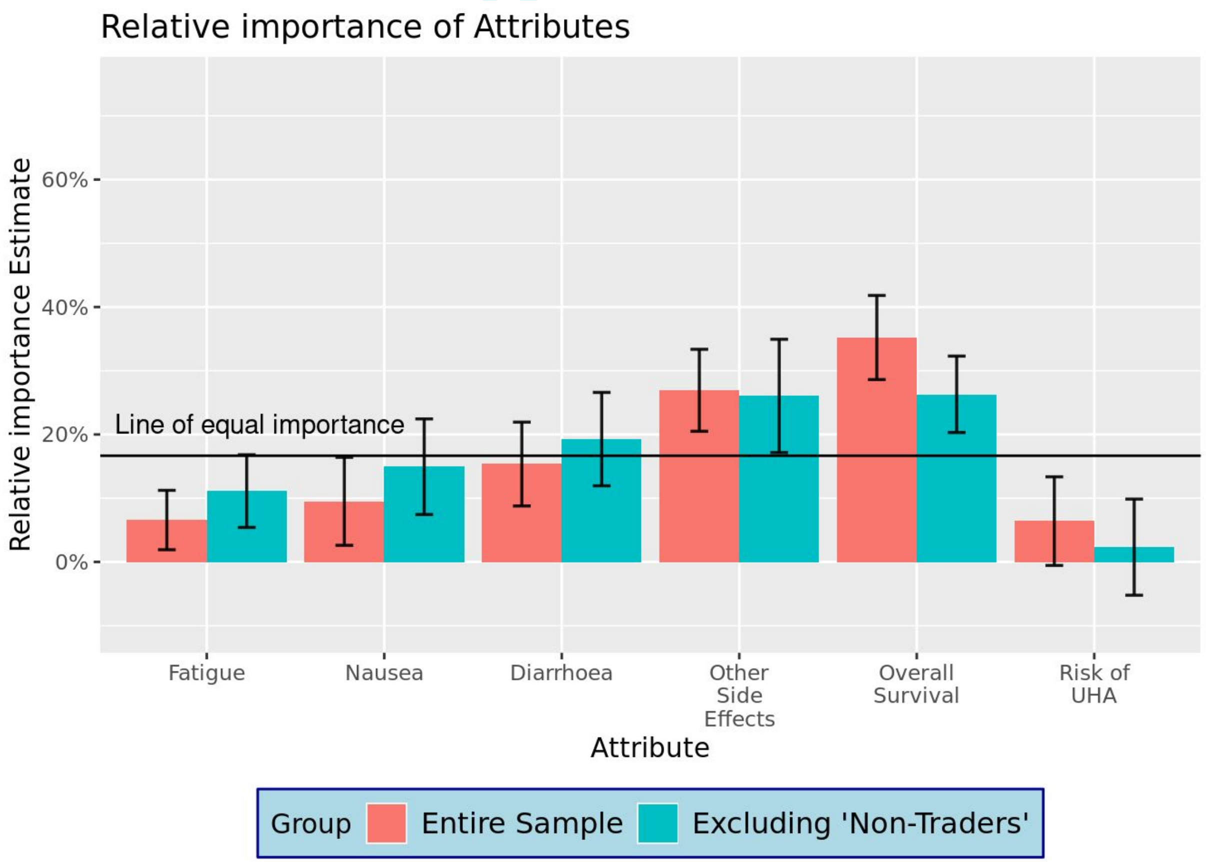

Error bars show 95% confidence interval using delta method standard errors

Figure A2 Comparison of Minimum Acceptable Survival between the entire sample and 'survival non-traders' (Sample excluding survival non-traders, N=71)

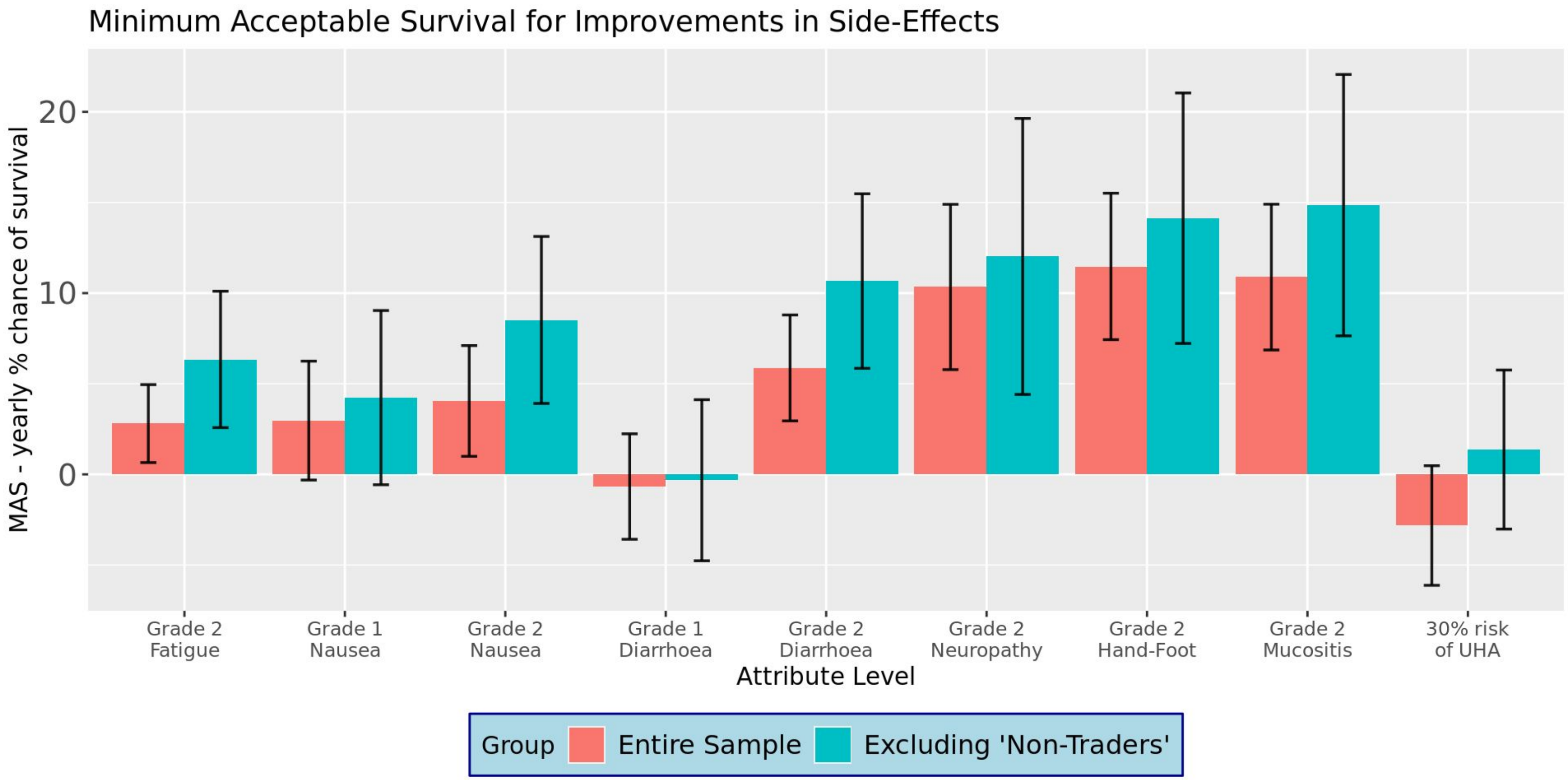

Error bars show 95% confidence interval using delta method standard errors

Figure A3 Comparison of Relative importance Estimates Between the Metastatic Breast Cancer Sample (N=72) and the Primary Breast Cancer Sample (N=33)

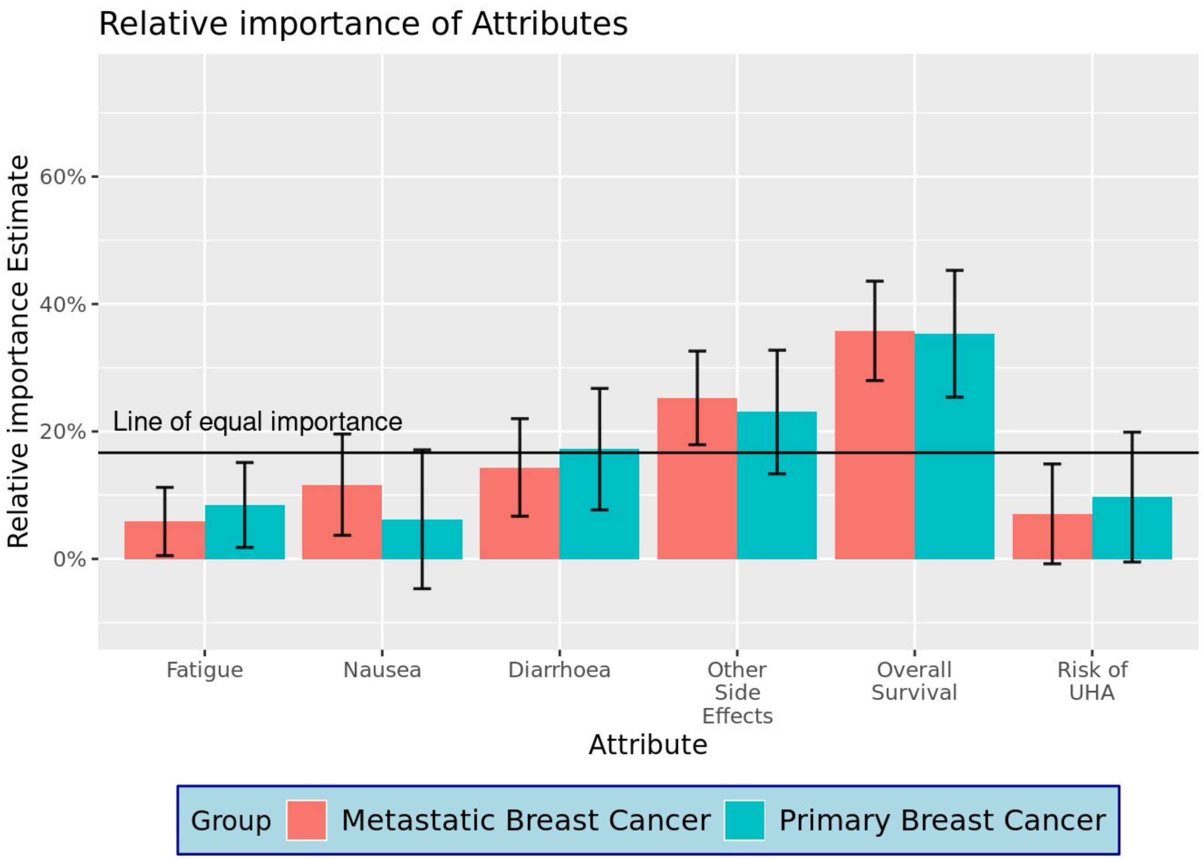

Error bars show 95% confidence interval using delta method standard errors

Figure A4 Comparison of Relative importance Estimates Between the Metastatic Breast Cancer Sample (N=72) and the Primary Breast Cancer Sample (N=33)

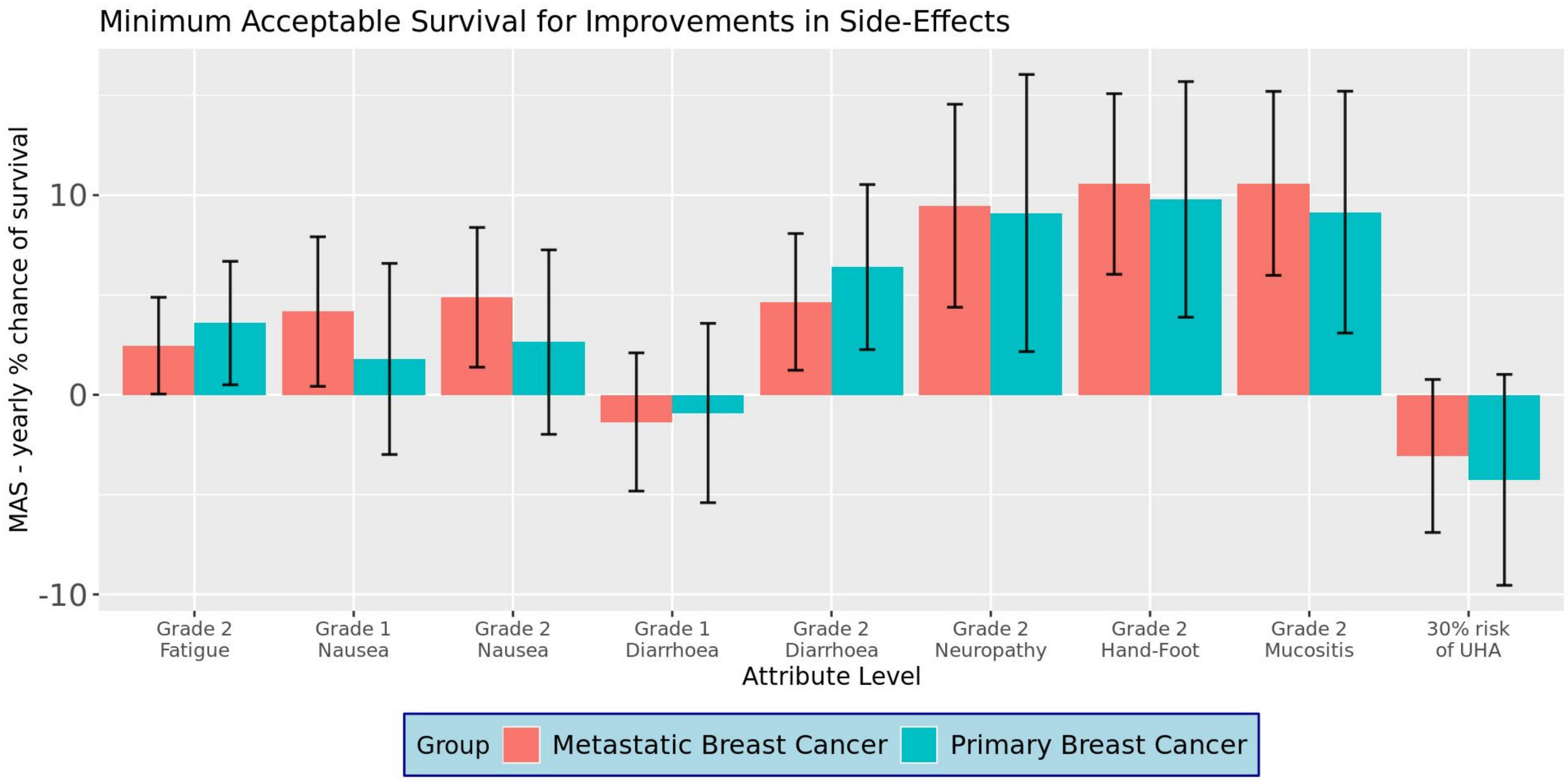

Error bars show 95% confidence interval using delta method standard errors

BMJ Open

Women's preferences for overall survival versus avoiding side effects in the treatment of metastatic breast cancer: a discrete choice experiment

Journal:	*BMJ Open*
Manuscript ID	bmjopen-2023-076798.R1
Article Type:	Original research
Date Submitted by the Author:	29-Nov-2023

Complete List of Authors:	Bullen, Alistair; The University of Edinburgh Usher Institute of Population Health Sciences and Informatics, Edinburgh Clinical Trials Unit<br>Ryan, Mandy; University of Aberdeen, HERU<br>Ennis, Holly; The University of Edinburgh<br>Gray, Ewan; The University of Edinburgh<br>Loría-Rebolledo, Luis Enrique ; University of Aberdeen, Health Economics Research Unit<br>McIntyre, Morag; Western General Hospital<br>Hall, Peter; The University of Edinburgh; Western General Hospital

**Primary Subject Heading**:	Health economics
Secondary Subject Heading:	Communication, Oncology, Patient-centred medicine
Keywords:	Quality of Life, Decision Making, Surveys and Questionnaires, Breast tumours < ONCOLOGY, HEALTH ECONOMICS

SCHOLARONE™ Manuscripts

I, the Submitting Author has the right to grant and does grant on behalf of all authors of the Work (as defined in the below author licence), an exclusive licence and/or a non-exclusive licence for contributions from authors who are: i) UK Crown employees; ii) where BMJ has agreed a CC-BY licence shall apply, and/or iii) in accordance with the terms applicable for US Federal Government officers or employees acting as part of their official duties; on a worldwide, perpetual, irrevocable, royalty-free basis to BMJ Publishing Group Ltd ("BMJ") its licensees and where the relevant Journal is co-owned by BMJ to the co-owners of the Journal, to publish the Work in this journal and any other BMJ products and to exploit all rights, as set out in our licence.

The Submitting Author accepts and understands that any supply made under these terms is made by BMJ to the Submitting Author unless you are acting as an employee on behalf of your employer or a postgraduate student of an affiliated institution which is paying any applicable article publishing charge ("APC") for Open Access articles. Where the Submitting Author wishes to make the Work available on an Open Access basis (and intends to pay the relevant APC), the terms of reuse of such Open Access shall be governed by a Creative Commons licence – details of these licences and which Creative Commons licence will apply to this Work are set out in our licence referred to above.

Other than as permitted in any relevant BMJ Author's Self Archiving Policies, I confirm this Work has not been accepted for publication elsewhere, is not being considered for publication elsewhere and does not duplicate material already published. I confirm all authors consent to publication of this Work and authorise the granting of this licence.

For peer review only

Women's preferences for overall survival versus avoiding side effects in the treatment of metastatic breast cancer: a discrete choice experiment

Authors Alistair J Bullen[1*]

Mandy Ryan[2]

Holly Ennis[1]

Ewan Gray[1]

Luis E Loría-Rebolledo[2]

Morag McIntyre[3]

Peter S Hall[1,3]

Affiliations

1. Edinburgh Health Economics, University of Edinburgh, Edinburgh Clinical Trials Unit, 9 Little France Rd, Edinburgh, EH16 4UX, United Kingdom
2. Health Economics Research Unit, University of Aberdeen, Polwarth Building, Foresterhill, Aberdeen, AB25 2ZD, United Kingdom
3. Edinburgh Cancer Centre, NHS Lothian, Western General Hospital, Crewe Road South, Edinburgh, EH4 2XU, United Kingdom

* Corresponding author: alistair.bullen@gcu.ac.uk

Conflicts of interest: All authors declare no conflicts of interest.

Keywords

Breast neoplasms; quality of life; decision making, shared; patient preference; surveys and questionnaires

3954 words

For peer review only

Abstract

Background: There has been a recent proliferation in treatment options for patients with metastatic breast cancer. Such treatments often involve trade-offs between overall survival and side effects. **Methods:** We designed a discrete choice experiment (DCE) to look at preferences for avoiding severity levels of side effects when choosing treatment for metastatic breast cancer. Treatment attributes were: fatigue; nausea; diarrhoea; other side effects (peripheral neuropathy, hand foot syndrome, and mucositis**);** urgent hospital admission and overall survival. Responses were analysed using an error component logit model. We estimated the relative importance of attributes and minimum acceptable survival for improvements in side effects. **Results:** 105 respondents participated, comprising of 72 metastatic breast cancer patients and 33 primary breast cancer patients. Overall survival had the largest relative importance, followed by other side effects, diarrhoea, nausea, and fatigue. Risk of urgent hospital admission was not significant. Whilst overall survival was the most important attribute, respondents were willing to forgo some absolute probability of overall survival for reductions in all Grade 2 side effects (12.02% for hand foot syndrome; 11.01% for mucositis; 10.42% for peripheral neuropathy, 6.33% for diarrhoea, and 3.62% for nausea). Grade 1 side effects were not significant, suggesting respondents have a general tolerance for them. **Conclusion:** Women are willing to forgo overall survival to avoid particular severity levels of side effects. Our results have implications for data collected in research studies and can help inform person-centred care and shared decision making.

For peer review only

Strengths and limitations of this study

- Our study is the first to elicit preferences for the treatment of metastatic breast cancer in the United Kingdom.
- The attributes chosen for the discrete choice experiment are highly general and refer to side effects shared by a variety of treatments. They are useful for making general comparisons between a wide array of treatments but less applicable for more nuanced choices that might offer small differences and are associated with side effects we did not investigate.
- Due to difficulties recruiting participants we were required to use a joint sample of metastatic and primary breast cancer patients when ideally the primary sample would consist only of metastatic breast cancer patients

For peer review only

Introduction

There are 35,000 people in the UK living with metastatic breast cancer (mBC) (1). mBC occurs if the cancer spreads to another part of the body at which point the cancer is usually considered incurable. The focus of treatment then shifts from curing the disease to managing it, slowing further progression and palliating symptoms. There is a dichotomy at the core of discussions surrounding treatment in this context, namely the trade-off between overall survival (OS) and the side effects patients must tolerate (2). Different treatments offer variable prospects for survival versus side effects. Treatment decisions are made more complex by the proliferation of new medicines for the treatment of mBC, ranging from cytotoxic chemotherapy to hormone therapies. Recent new additional options include immunotherapy and targeted small molecules (3).

Such developments mean that breast cancer patients must navigate difficult decisions between complex and unfamiliar treatments (4). Greater patient involvement in decision-making is needed to allocate the treatment which best addresses their needs. Recent guidelines have emphasised the requirement for shared decision-making across the NHS (5, 6). Although shared decision-making is widely practised its implementation needs improvement, specifically regarding doctor-patient communication (7). Evidence from patient preference studies reveal trends to be considered by healthcare providers during consultations. Patient preferences are also important for the authors of healthcare guidelines that inform policy around which drugs should be provided. As a final example, they are important for developers of new cancer drugs when they provide guidance on what patients will tolerate concerning side effects for improvements in survival.

Discrete Choice Experiments (DCEs), sometimes referred to as conjoint analysis, are increasingly used to estimate patient preferences, looking at the relative importance of attributes as well as the trade-offs individuals are willing to make (8). A recent systematic review of the application of DCEs to oncology treatment identified 79 studies, with patient preferences for breast cancer (n =10, 13%) as the most common area of application (9). The review found the most common outputs were relative

For peer review only

importance of attributes and marginal rates of substitution (MRS, trade-offs) in terms of (in order of frequency): willingness to pay (WTP); minimum acceptable benefit; minimum acceptable risk; and willingness to accept non-risk for benefit and willingness to travel. Whilst clinical efficacy attributes were commonly ranked as most important, with OS and Progression Free Survival ranked most important by 90% and 30% respectively by patient samples across all cancer types, respondents were often willing to trade clinical efficacy for improvements in side effects. A similar result was found in a systematic review of patient preference studies relating to breast cancer treatment (10). These two systematic reviews identified six DCEs that assessed preferences for mBC drug treatments (11-16). These studies also show that whilst treatment efficacy (OS or PFS) is important, and often the most important factor, patients also value avoiding the side effects of different treatments (11, 14-16). Two of these mBC studies estimated the value of avoiding side effects in monetary terms (willingness to pay, a monetary measure of benefit) (13, 14). We use the DCE methodology to investigate how much absolute probability of OS women are willing to give up to avoid a particular severity level of side effects in the treatment of mBC. We refer to this as Minimum Acceptable Survival (MAS). We also focus on the severity of side effects, whereas the existing DCEs have focussed mainly on the risk of side effects, and the preferences for long-term survival. Our study is also the first to elicit preferences for the treatment of mBC in the UK; preferences across countries may differ due to cultural factors and different healthcare systems. For example, Southeast Asian attitudes to cancer management and death are known to be different to Western ones (17).

Methods

The DCE is a choice-based survey that quantifies preferences for alternatives (e.g. treatment options for mBC) where alternatives are described by their attributes and associated levels (18). In our DCE alternatives are treatments, attributes are treatment characteristics (e.g. survival and side effects), and levels are values associated with treatment characteristics (e.g. % chance of survival, possible levels of severity for nausea).

For peer review only

Defining attributes and levels

Four work packages (WPs) informed the attributes and levels: (i) a targeted literature review of qualitative literature concerning the patient experience of metastatic cancer; (ii) a targeted literature review of DCEs centred on treatments for metastatic cancer; (iii) a thematic analysis (19) of Scottish Medicine's Consortium (SMC) Patient and Clinical Engagement (PACE) statements for mBC treatments; and (iv) face-to-face interviews with mBC patients. All work involving face-to-face patient contact was completed by a research nurse and research assistant both of whom had been trained in qualitative methods. For more information on all WPs see Supporting Information 1. The research group, consisting of breast cancer and DCE experts, considered these attributes, reducing them to a manageable number for use in the DCE framework. Attribute selection and layperson definitions were developed using think-aloud interviews with patients (20).

The final attributes and levels are shown in Table 1, with patient definitions of attributes defined in Table A1 in Supporting Information 2. Levels are intended to represent possibilities for first-line treatment following a diagnosis at Stage IV (metastatic breast cancer). Side effects were: fatigue; nausea; diarrhoea; and additional side effects (peripheral neuropathy, hand foot syndrome, and mucositis as mutually exclusive levels). Levels of side effects attributes were described using plain-language translations of the Common Terminology Criteria for Adverse Events (CTCAE) (21) criteria (Table A1). These were developed with health professionals and tested in the developmental piloting work. Following piloting with patients, and to ease understanding, fatigue was referred to as tiredness. The nausea attribute combined the corresponding CTCAE grades nausea and vomiting (since they tend to accompany one another). Attribute levels ranged from a zero level of toxicity up to Grade 2. Choice options were discussed with health professionals to ensure plausibility. During these discussions it was suggested that some background fatigue is expected for most patients; therefore Grade 1 fatigue was the minimum level of the attribute. It was also advised that in the presence of Grade 3 adverse events, treatment would be discontinued; thus, the maximum level for all adverse

For peer review only

141 event attributes was Grade 2. The additional side effects attribute was included to capture a broader range of side effects while limiting the number of attributes and therefore the cognitive burden of completing the choice tasks (22). It differed from competing attributes due to each level corresponding to a unique side effect, Grade 2 descriptions were used so that we could compare preferences for the equivalent highest level of the diarrhoea and nausea attributes.

Patient and Public Involvement

Patients with mBC were invited to, and participated in, interviews and in-person questionnaire piloting sessions, both of which informed the final design of the survey.

For peer review only

Table 1 Attributes and Levels for the Discrete Choice Experiment

Attributes	Levels	Definition	Regression Equation Label	Regression Equation Preference parameter
Fatigue*	Grade 1 Fatigue (reference level)<br>Grade 2 Fatigue	Tiredness - In this scenario your cancer will always make you more tired than you once were. But treatments can make this worse	G2_FAT	β_1
Nausea	No nausea (reference level)<br>Grade 1 Nausea<br>Grade 2 Nausea	Treatments may cause nausea and nausea may cause you to vomit.	G1_NAU<br>G2_NAU	β_2<br>β_3
Diarrhoea	No diarrhoea (reference level)<br>Grade 1 Diarrhoea<br>Grade 2 Diarrhoea	Treatments may cause diarrhoea.	G1_DIA<br>G2_DIA	β_4<br>β_5
Additional side effects	No other side effects (reference level)<br>Grade 2 Peripheral Neuropathy<br>Grade 2 Hand foot syndrome<br>Grade 2 Mucositis	A treatment may be associated with an additional side effect. These side effects include peripheral neuropathy (nerve damage), hand foot syndrome (severe skin problems), and mucositis (mouth ulcers). You can experience a maximum of one of these side effects on a given treatment.	G2_NEU<br>G2_HAN<br>G2_MUC	β_6<br>β_7<br>β_8
Overall survival	60 alive at 1 year, 8 alive at 5 years<br>65 alive at 1 year, 12 alive at 5 years<br>75 alive at 1 year, 24 alive at 5 years	How long someone lives is always uncertain but in this scenario the care team is able to tell you how many patients are expected to be alive after 1 and 5 years. They are also able to tell you how many of those who survived the first year also experienced an urgent hospital admission. A patient may, for example, have an urgent hospital admission because of a severe infection (sepsis) or because of extreme symptoms. Hospital admission and survival statistics will both be presented in a single graphic. Please imagine that the figure for urgent hospital admissions includes hospital stays which range from days to weeks.	OS	β_9
Risk of urgent hospital admission	1/100 people<br>10/100 people<br>30/100 people		UHA	β_{10}

* Following piloting, and to ease understanding, fatigue was referred to as tiredness.

A risk of urgent hospital admission (UHA) was included, defined as the number of people from 100 treated who would be admitted to the hospital for a UHA. The decision to make UHA a probabilistic attribute was motivated by discussions with health professionals. It was suggested that, unlike Grade 1 and Grade 2 toxicities, a treatment which guaranteed a UHA would not be offered to patients. OS was defined as the annual probability of survival, which was time constant and represented the probability of surviving in the present and future years. To account for short and long-term preferences (23) annual probability of survival was presented as frequencies at 1 and 5 years e.g. 65% translated to 65 people alive a 1 year and 12 alive at 5 years (the rounded result of 100×0.65^5). Risk is generally not well understood by the general public (**24**), therefore 1 and 5-year survival were presented alongside one another to illustrate the effects of cumulative probability to respondents. The average 1-year survival rate after diagnosis for an mBC patient is approximately 65% (25); we chose this as our central value for our annual survival rate. We used an exponential calculation for 5-year survival, rather than real-world data, to simplify the choice task to include only one risk attribute. The levels for UHA were defined following discussions with health professionals.

It was observed during piloting that some of the expected negative preference for UHA would occur due to a risk of death. Respondents often struggled to disentangle and interpret the related attributes. To isolate the effect independently from the risk of death a graphic was devised, which showed levels of both attributes. The combination of frequencies and tree diagrams has been shown to improve understanding of risks (26, 27). The first row reports the number of patients admitted to the hospital for a UHA, and the second and third show 1- and 5-year survival respectively. Frequencies for positive outcomes (no hospitalisation and survival) and negative outcomes (hospitalisation and death) were both communicated in an attempt to address framing bias (28).

<u>Choices presented to individuals</u>

Ngene (Choice Metrics) was used to create a set of choices from which preferences could be estimated for all possible scenarios; the design was D-efficient, which minimized the variance-

For peer review only

covariance of the measures of average preference (29). This resulted in a set of 12 choice tasks. All choices included a no-treatment option, with side effects defined as the least severe level and risk of UHA 0%. To define the opt-out level of survival respondents were asked what they perceived their chances of survival at 1 and 5 years, resulting in a 45% average level. This was consistently lower than all levels of OS with treatment and judged reasonable given survival at one year among stage 4 breast cancer patients diagnosed in England in 2013 was between 16-43% depending on age, with a mode of 43% (30). The choice context is shown below.

The choice scenario

The scenario You are being asked to consider the decision you would make if presented with different metastatic breast cancer treatments. For each question there are only 2 treatment options. If you choose a treatment, the other treatment will not be an option to you in the future. We ask you to imagine that no other treatment options will become available to you in the future. You also have the option to choose to have no treatment. With no treatment you would experience the symptoms of your cancer; your cancer will be left to progress and you will have shorter life expectancy as a result.

The treatments Both treatments are in the form daily pills. Both treatments can treat you for the rest of your life. You would be allowed to stop treatment whenever you wished. Both treatments have different benefits and side effects.

Side effects Side effects are guaranteed. Side effects are already being managed with the best available medicines and care. You will still experience a side effect for weeks at a time.

Following developmental work, the twelve choices were divided into two blocks of six choice tasks to mitigate mental fatigue effects (31). Respondents were randomly allocated to one of the design blocks and choice tasks were presented in a randomised order. Respondents were given a warm-up choice task (Fig 1) to complete.

Figure 1: Example of DCE choice task (Warm-up task)

[Fig 1]

For peer review only

Data Analysis

The following utility/benefit function was estimated using Error Component Mixed Logit regression:

$$U_{in} = \beta_0 Treat_i + \beta_1 G2\_FAT_{i1} + \beta_2 G1\_NAU_{i2} + \beta_3 G2\_NAU_{i3} + \beta_4 G1\_DIA_{i4} + \beta_5 G2\_DIA_{i5} + \beta_6 G2\_NEU_{i6} + \beta_7 G2\_HAN_{i7} + \beta_8 G2\_MUC_{i8} + \beta_9 OS_{i9} + \beta_{10} UHA_{i10} + \varepsilon_{in}$$

U_{in} represents the utility for individual n for alternative i. The attribute variables are defined in Table 1. β_1 to β_8 are modelled as dummy variables, showing the value of that attribute level relative to the reference (best) level. β_9 and β_{10} are modelled as continuous variables, showing the value of a % change in OS and UHA. The signs of the β parameters indicate whether the effect of the attribute level on preference is positive or negative. All side effects preference parameters are expected to have a negative sign relative to the reference level. Respondents are expected to prefer higher OS, resulting in a positive β_9. The preference for chance of UHA, β_{10}, is expected to have a negative sign, with lower values preferred. εin represents the unobserved error component. β_0 shows the general preference for treatment over no treatment (everything else equal) with a positive sign indicating a general preference to receive treatment (everything else equal). An error component is assumed by specifying β_0 as random normally distributed, thus allowing for flexible substitution between alternatives and dropping the irrelevant alternatives assumption (**32**), we run 100 draws using the Halton sequence.

We used the parameter values to estimate the relative importance of attributes (33); this is calculated as the difference in the range of attribute's variable values. We calculate percentages from these relative ranges, obtaining a set of attribute importance values that add to 100%. We also estimate MRS in the form of MAS for improvements in side effects using the rate for 1-year OS in the calculation, estimated as $\frac{\beta_x}{-\beta_9}$. For example, $\frac{\beta_1}{-\beta_9}$ shows MAS for a reduction in side effects from

For peer review only

Grade 2 fatigue to Grade 1 fatigue and $\frac{\beta_4}{-\beta_9}$ shows MAS for a reduction in side effects from Grade 1 diarrhoea to no diarrhoea.

Sample and Recruitment

Calculating an optimal sample size for newly designed DCEs is problematic as it depends on the true values of the unknown parameters for which the analysis intends to estimate (34). Previous DCEs in the area of metastatic cancer of a similar design have demonstrated that reliable analysis can be performed with samples of 100 or fewer participants (35-37). We therefore aimed to recruit 100 patients as a minimum threshold.

Our target sample was initially women who had experienced metastatic breast cancer. Given the anticipated challenges of recruiting a sufficient number of women who had an mBC diagnosis, we also collected preferences from women who had experienced primary breast cancer. Respondents who responded that they had only a primary breast cancer were asked to imagine that they had received a secondary breast cancer diagnosis in the introductory text. The preferences of metastatic breast cancer patients were compared to primary breast cancer patients.

The DCE was administered using an online link between January and March 2020. Recruitment methods included: (i) distribution of leaflets at cancer centres and conferences; (ii) an online panel provided by Dynata; (iii) social media engagement with help from breast cancer charities; and (iv) a research nurse approaching patients directly during clinic visits and inviting them to complete the survey on a tablet device. Interviewed respondents provided informed written consent before interviews proceeded. Survey respondents self-reported as UK residents over 18 years of age and provided informed consent online at the start of the survey.

Results

The sample size was 105 (Table A2 in Supporting Information 2). All identified as female. Seventy-two respondents were mBC patients and 33 were primary breast cancer patients.

10 respondents did not complete all 6 choice tasks, resulting in 29 missing choice tasks. Completed choice tasks were included in the analysis. Of 601 responses to choice tasks across all participants, 38 (6.32%) were for no treatment. These were selected by 16 women, with three women always choosing the opt-out option. 32.38% (N=34) of respondents always chose the option with the highest OS. Some of these respondents may have been using a simplifying heuristic, nonetheless, we focus our analysis on the complete sample as it is not possible to distinguish respondents who are demonstrating a genuine preference and those using a simplifying heuristic. (Figures A1 and A2 in Supporting Information 3 compare analyses when excluding the 34 potential non-traders; as expected the relative importance of OS is lower and participants have a higher MAS. However, samples are too small to demonstrate statistically significant differences.)

Table 2 shows the error-component logit regression results for all respondents (Table A3 in supporting information 2 shows the results of the equivalent multinomial logit) and Fig 2 shows the relative importance of attributes. We also ran an alternative specification as multinomial logit where the overall survival attribute was dummy coded and it demonstrated a near linear relationship between effect and survival gain between the 60 and 75 levels which suggests the specification of overall survival as a constant variable is appropriate (Supporting Information 3, Table A4).

Figure 2: Relative Importance of Attributes

[Figure 2]

Error bars show 95% confidence interval using delta method standard errors

For peer review only

MAS estimates (Table 2, column 8 and Fig 3) show respondents' willingness to forgo OS to avoid all Grade 2 toxicities.

Figure 3: Minimum acceptable survival to Avoid Side effects

[Figure 3]

Error bars show 95% confidence interval using delta method standard errors

Results comparing mBC patients and primary breast cancer patients are shown in Figures A3 and A4 Supporting Information 3. The most notable difference is the estimated importance of the nausea attribute, nonetheless, there are no statistically significant differences between any of the estimates.

For peer review only

Table 2 Error Component Logit

		Estimate	p	95% CI Lower bound	95% CI Upper bound	Relative attribute importance	Minimum acceptable survival
Alternative Specific Constant	Treatment	5.1339	0.0002	2.4566	7.8112	-	-
	Standard Deviation of Treatment	4.0982	0.0001	2.0411	6.1553		
Fatigue	Grade 2 fatigue	-0.2948	0.0101	-0.5194	-0.0702	0.0590	2.5412
Nausea	Grade 1 nausea	-0.4196	0.0519	-0.8426	0.0034	0.1091	3.6178
	Grade 2 nausea	-0.5446	0.0093	-0.9550	-0.1342		4.6960
Diarrhoea	Grade 1 diarrhoea	0.0241	0.8806	-0.2898	0.3379	0.1519	-0.2074 N.S.
	Grade 2 diarrhoea	-0.7343	0.0004	-1.1384	-0.3302		6.3314
Additional side effects	Grade 2 peripheral neuropathy	-1.2087	0.0000	-1.6458	-0.7715	0.2793	10.4211
	Grade 2 hand foot syndrome	-1.3946	0.0000	-1.8404	-0.9489		12.0247
	Grade 2 mucositis	-1.2764	0.0000	-1.6668	-0.8861		11.0055
Overall survival	Annual probability of survival	0.1160	0.0000	0.0847	0.1473	0.3485	-
Urgent Hospital Admission	Probability of urgent hospital admission in the first year of treatment	0.0090	0.1068	-0.0019	0.0199	0.0522 N.S.	-2.3236 N.S.(for 30% level)
**Model statistics**							
Number of individuals	105						
Observations	601						
Log likelihood	-379.9434						
Bayesian info criterion	836.6699						

N.S. not significant

Discussion

We provide new evidence on UK women's preferences for the treatment of mBC. Respondents had a general preference for treatment, indicated by the low opt-out rates which result in a positive constant term (Treat). As expected, they preferred treatments with higher OS, in fact almost a third of the sample (32.38%) always chose the treatment option with a higher OS. All Grade 2 toxicities were significant and negative, suggesting negative preferences for these attribute levels. However, Grade 1 nausea and diarrhoea were not significant, suggesting patients are indifferent when compared to having none of these side effects There was no significant effect of UHA on respondents' choices.

The relative importance of OS exceeded all other attributes, with an overall importance score of 34.85%. The remaining relative importance was distributed accordingly: additional side effects (27.93%), diarrhoea (15.19%), nausea (10.90%), fatigue (5.90%), and risk of urgent hospital admission (5.22%). Respondents would accept a reduction in the probability of survival of 2.54% to avoid Grade 2 fatigue (and have Grade 1 fatigue). The MAS associated with levels of the additional side effects were particularly high: respondents were willing to give up 10.42%, 12.02%, and 11.01% chance of OS for total avoidance of grade 2 peripheral neuropathy, grade 2 hand foot syndrome, and grade 2 mucositis respectively. Notably, Grade 1 nausea and diarrhoea were acceptable to patients and did not significantly impact patients' choices. Thus, they were not willing to give up survival for improvements in such Grade 1 side effects. However, Grade 2 side effects were disliked and respondents were willing to forgo up to 12.02% OS to avoid such severe side effects.

Our results add to a growing literature showing that breast cancer patients value avoiding the side effects of treatments, and are willing to forgo some level of treatment efficacy to achieve this (9,10). Directly comparing preference estimates between studies is often inappropriate as estimates only apply to the attributes and levels within the choice framework of DCE from which they are derived. Nonetheless, it is important to highlight the findings of other studies and draw comparisons where appropriate. Our results appear to align somewhat with DiBonaventura *et al's.* (11) exploration of the

For peer review only

preferences of women with mBC in the USA who also found that OS was the most important attribute. Additionally, side effects (alopecia, fatigue, neutropenia, motor neuropathy, and nausea/vomiting) and dosing regimen were also important. The remaining studies did not include attributes for overall survival but did identify statistically significant preferences for side-effect avoidance. For example, Omori *et al.* (15) explored the preferences of Japanese postmenopausal patients with HR+ breast cancer for the treatment of mBC. They conclude that women preferred treatments that extend PFS despite potential grade 2 diarrhoea. However, when diarrhoea severity increased to grade 3, patients were more willing to sacrifice PFS to avoid more frequent diarrhoea. In contrast, exploring preferences of women diagnosed with mBC in Germany, Spaich *et al* (16) concluded that severe neutropenia was the most important attribute, followed by alopecia, neuropathy and PFS. Two studies have explored the preferences of women diagnosed with mBC in the USA, estimating value in monetary terms. Lalla et al (12) found that women were willing to pay the most to avoid severe diarrhoea (US$3,894 a year), followed by avoidance of hospitalization due to infection (US$3,279), severe nausea (US$3,211) and severe peripheral neuropathy (US$2,764). MacEwan et al (13) found that women were willing to pay US$1930 per month for treatment, with US$63 per month for each 1% reduction in the risk of moderate to severe side effects. In a similar study in Thailand, Ngorsuraches and Thongkeaw (14) found respondents were willing to pay US$151.6 per month for every 1 month increase in PFS compared to US$69.8 and US$278.3 per month for every 1% decreased risk of anaemia and pneumonitis respectively.

Our results imply that treatment efficacy and OS are not the only endpoints of value to women with mBC (and indeed oncology more broadly). Furthermore, there is evidence that the CTCAE grading criteria do not scale in parallel with patients' preferences; for example, Grade 2 nausea is preferred to Grade 2 hand foot syndrome (indicated by a lower negative preference parameter). Grade 1 toxicities were not significant, suggesting they are relatively tolerable to patients (compared to having no side effects). These findings suggest that clinician-reported and objectively graded toxicities may not correspond to patients' values and support the further incorporation of Patient Reported Outcomes (PROs) and preference studies in the study of new medicines for mBC. PROs are increasingly

For peer review only

accepted by the US Food and Drug Administration (FDA) and European Medicines Agency (EMA) (38) and the National Institute for Health and Care Excellence (NICE) has begun to accept patient preference studies alongside traditional evidence such as cost per QALY (39).

Our study has focused on the preferences of patients. Given that health professionals often make treatment decisions/recommendations for patients, a fruitful area for future research is to compare the preferences of patients and doctors. Current research suggests that it is common for there to be a mismatch in the preferences of patients and healthcare providers (40). Given health professionals possess greater information on treatments and patients possess private information on their values and priorities, Decision Aid Tools (DAT) can help understand and bridge this mismatch as part of shared decision making. The focus of such DATs within breast cancer has been on the detection and prevention of early breast cancer (41). The work presented in this paper contributes to the groundwork for the use of a DCE as a DAT to promote shared decision making and person-centred care. A limited number of studies have adapted DCEs into DATs: Dowsey *et al.* (42) used a DCE as part of a decision aid for patients undergoing total knee arthroplasty; Hazlewood *et al*. (43) evaluated a proof-of-concept DAT for patients with early rheumatoid arthritis, which included a DCE to assist respondents in choosing initial treatment and Loria-Rebolledo *et al*. (44) are exploring the use of DCEs to estimate preferences at the individual level for use in a shared decision making setting.

There are limitations to this study. Firstly, the sample size was small, and we were required to supplement the mBC patient sample with primary breast cancer who were asked to imagine a secondary diagnosis. Although the analysis did not present large enough differences in preferences to suggest this meaningfully affected results, a larger sample would allow the possibility of preference heterogeneity to be extensively explored. Preferences, trade-offs and willingness to avoid particular side effects may be influenced by many factors. One potential area for future research is understanding the dynamics of treatment preferences and response shift. This may be particularly important for end-of-life care, which mBC patients may face (45). Other factors that may influence preferences include specific cancer diagnosis, location of metastases, multiple diagnoses, and

For peer review only

treatment experience. Future research should collect data on the characteristics of respondents which could be used to explore preference heterogeneity. Secondly, national data indicates that the highest incidence of new breast cancer cases (any stage) for women between 2015 and 2017 was aged 60-69 (46), suggesting our sample is younger with the largest group aged 50-59. A 2008 survey in the United States found a stronger preference for quality of life than quantity of life among cancer patients (47), if this effect exists in our population the preference weights may be positively skewed. Thirdly, the argument could be made that the description of how side effects are experienced in the choice scenario may be difficult for patients to understand. The decision to focus on symptom severity and to avoid clear definitions of symptom frequency relating to side effects was made to alleviate the cognitive burden of the task by simplifying the information presented. We opted to represent uncertainty by suggesting that treatments were indefinite and side effects would therefore be indefinitely experienced "for weeks at a time". Some would argue that in doing so we forgo a degree of clarity of interpretation for respondents and consequently the results of the study. Fourthly, we simplified the choice task to include only one risk attribute, we used an exponential function to estimate the five-year survival rate. Future research could include two attributes, one and five-year survival, with the latter based on real data. Preferences for short and long-term survival could then be estimated. Fifthly, in defining the no treatment option, the level for OS was defined as the mean value from women's perceived OS without treatment. Results may have differed if we informed respondents of their chance of survival without treatment. Furthermore, the baseline levels for side-effect attributes were assumed to be the minimum possible realistic levels, however, respondents may have implicitly considered unique individual baselines based on lived experience. The interpretation of the no treatment option may have differed between respondents and may have caused some attribute levels to appear acceptable for respondents who considered them to be the same as baseline, potentially dampening their overall effect within the sample. Results may be more precise if we estimated preferences within a more sophisticated design which adjusted for respondents' baseline levels. Finally, whilst the insignificance of the risk of UHA may be a genuine preference, the result may also reflect a difficulty in understanding this attribute. Despite low relative importance, similar

For peer review only

attributes are significant in other metastatic cancer DCEs, however, the attribute levels are more severe (37, 48). Future work should explore explaining this attribute.

In conclusion, our results provide evidence that patients are willing to give up some survival benefits to avoid severe levels of side effects. Future therapeutic studies should ensure such data is collected to ensure that the patient can make an informed decision when making treatment decisions. Future research should explore using such information within a shared decision-making framework.

For peer review only

Data Sharing

The dataset used for this analysis is available from the University of Edinburgh Datashare, https://datashare.ed.ac.uk/handle/10283/4436.

Funding

This research was supported by a charitable grant from the Edinburgh and Lothians Health Foundation (Scottish Registered Charity No: SC007342). Mandy Ryan and Luis E Loría-Rebolledo are funded by the University of Aberdeen and the Chief Scientist Office of the Scottish Government Health and Social Care Directorates. The funders had no role in considering the study design or in the collection, analysis, or interpretation of data, the writing of the paper, or the decision to submit the article for publication.

Ethics

The protocol was approved by the National Health Service (NHS) North of Scotland Research Ethics Committee (REC ref: 19/NS/0066). All participants provided informed consent for every stage of the research.

Author Contributions

PH, EG, HE and MR contributed to the conceptualisation of the project and funding application. All authors contributed to the design of the study. AB developed the DCE, with experimental design support from MR. AB and MM collected the data. AB conducted the statistical analysis with support from LL. All authors contributed to the interpretation of the data as well as the drafting and revision of the manuscript. All authors gave final approval and agreed to be accountable for all aspects of the work.

For peer review only

Acknowledgements

We thank all the patients and volunteers who agreed to participate in this study and the study contributors.

For peer review only

442 # References

443 1) Feinmann J. Number of UK patients with metastatic breast cancer isn't known. BMJ
444 2017;359:j5205

445 2) Brom L, Pasman HR, Widdershoven GA, van der Vorst MJ, Reijneveld JC, Postma TJ,
446 Onwuteaka-Philipsen BD. Patients' preferences for participation in treatment decision-making
447 at the end of life: qualitative interviews with advanced cancer patients. PloS one. 2014 Jun
448 25;9(6):e100435.

449 3) Waks AG, Winer EP. Breast cancer treatment: a review. Jama. 2019 Jan 22;321(3):288-300.

450 4) Mosher CE, Johnson C, Dickler M, Norton L, Massie MJ, DuHamel K. Living with
451 metastatic breast cancer: a qualitative analysis of physical, psychological, and social sequelae.
452 The breast journal. 2013 May;19(3):285-92.

453 5) Scottish Government. What Works to Support and Promote Shared Decision Making: A
454 synthesis of recent evidence. 2019. Available from: https://www.gov.scot/publications/works-
455 support-promote-shared-decision-making-synthesis-recent-evidence/pages/3/. Last accessed
456 7th March 2022.

457 6) NICE. Shared Decision Making Collaborative – An Action Plan. 2016. Available from:
458 https://www.nice.org.uk/Media/Default/About/what-we-do/shared-decision-making-
459 collaborative-action-plan.pdf. Last accessed 7th March 2022.

460 7) Covvey JR, Kamal KM, Gorse EE, Mehta Z, Dhumal T, Heidari E, Rao D, Zacker C. Barriers
461 and facilitators to shared decision-making in oncology: a systematic review of the literature.
462 Supportive Care in Cancer. 2019 May;27(5):1613-37.

463 8) Soekhai V, de Bekker-Grob EW, Ellis AR, Vass CM. Discrete choice experiments in health
464 economics: past, present and future. Pharmacoeconomics. 2019 Feb;37(2):201-26.

For peer review only

9) Collacott H, Soekhai V, Thomas C, Brooks A, Brookes E, Lo R, Mulnick S, Heidenreich S. A systematic review of discrete choice experiments in oncology treatments. The Patient-Patient-Centered Outcomes Research. 2021 Nov;14(6):775-90.

10) Guerra RL, Castaneda L, de Albuquerque RD, Ferreira CB, Correa FD, Fernandes RR, de Almeida LM. Patient preferences for breast cancer treatment interventions: a systematic review of discrete choice experiments. The Patient-Patient-Centered Outcomes Research. 2019 Dec;12(6):559-69.

11) DiBonaventura M, Copher R, Basurto E, Faria C, Lorenzo R. Patient preferences and treatment adherence among women diagnosed with metastatic breast cancer. American Health & Drug Benefits. 2014 Oct;7(7):386.

12) Lalla D, Carlton R, Santos E, Bramley T, D'Souza A. Willingness to pay to avoid metastatic breast cancer treatment side effects: results from a conjoint analysis. Springerplus. 2014 Dec;3(1):1-7.

13) MacEwan JP, Doctor J, Mulligan K, May SG, Batt K, Zacker C, Lakdawalla D, Goldman D. The value of progression-free survival in metastatic breast cancer: results from a survey of patients and providers. MDM policy & practice. 2019 Jun;4(1):2381468319855386.

14) Ngorsuraches S, Thongkeaw K. Patients' preferences and willingness-to-pay for postmenopausal hormone receptor-positive, HER2-negative advanced breast cancer treatments after failure of standard treatments. Springerplus. 2015 Dec;4(1):1-1.

15) Omori Y, Enatsu S, Cai Z, Ishiguro H. Patients' preferences for postmenopausal hormone receptor-positive, human epidermal growth factor receptor 2-negative advanced breast cancer treatments in Japan. Breast Cancer. 2019 Sep;26(5):652-62.

For peer review only

16) Spaich S, Kinder J, Hetjens S, Fuxius S, Gerhardt A, Sütterlin M. Patient preferences regarding chemotherapy in metastatic breast cancer—a conjoint analysis for common taxanes. Frontiers in oncology. 2018 Nov 21;8:535.

17) Ong KJ, Back MF, Lu JJ, Shakespeare TS, Wynne CJ. Cultural attitudes to cancer management in traditional South-East Asian patients. Australasian radiology. 2002 Dec;46(4):370-4.

18) Lancaster KJ. A new approach to consumer theory. Journal of political economy. 1966 Apr 1;74(2):132-57.

19) Braun V, Clarke V. Thematic analysis. American Psychological Association; 2012.

20) Cheraghi-Sohi S, Bower P, Mead N, McDonald R, Whalley D, Roland M. Making sense of patient priorities: applying discrete choice methods in primary care using 'think aloud' technique. Family practice. 2007 Jun 1;24(3):276-82.

21) National Cancer Institute (U.S.). Adverse events/CTCAE. 2021. Available: https://ctep.cancer.gov/protocoldevelopment/electronic_applications/ctc.htm. Last accessed 7th March 2022.

22) Bridges JF, Hauber AB, Marshall D, Lloyd A, Prosser LA, Regier DA, Johnson FR, Mauskopf J. Conjoint analysis applications in health—a checklist: a report of the ISPOR Good Research Practices for Conjoint Analysis Task Force. Value in health. 2011 Jun 1;14(4):403-13.

23) Chao LW, Szrek H, Pereira NS, Pauly MV. Time preference and its relationship with age, health, and survival probability. Judgment and decision making. 2009 Feb 1;4(1):1.

24) Peters E, Hibbard J, Slovic P, Dieckmann N. Numeracy skill and the communication, comprehension, and use of risk-benefit information. Health affairs. 2007 May;26(3):741-8.

For peer review only

25) Cancer Research UK. Breast survival by stage at diagnosis. Breast cancer survival statistics. 2019. Available from: https://www.cancerresearchuk.org/health-professional/cancer-statistics/statistics-by-cancer-type/breast-cancer/survival#heading-Three. Last accessed 11th November 2022.

26) Kurz-Milcke E, Gigerenzer G, Martignon L. Transparency in risk communication: graphical and analog tools. Annals of the New York Academy of Sciences. 2008 Apr;1128(1):18-28.

27) Spiegelhalter D, Pearson M, Short I. Visualizing uncertainty about the future. science. 2011 Sep 9;333(6048):1393-400.

28) Howard K, Salkeld G. Does attribute framing in discrete choice experiments influence willingness to pay? Results from a discrete choice experiment in screening for colorectal cancer. Value in health. 2009 Mar;12(2):354-63.

29) ChoiceMetrics.NGENE (1.2). 2018. [NGENE]. [Accessed 20 January 2020].

30) Office for National Statistics. Cancer survival in England: adult, stage at diagnosis and childhood - patients followed up to 2018. 2019. Available from: https://www.ons.gov.uk/peoplepopulationandcommunity/healthandsocialcare/conditionsanddiseases/bulletins/cancersurvivalinengland/stageatdiagnosisandchildhoodpatientsfollowedupto2018. Last accessed 21st January 2021.

31) Bech M, Kjaer T, Lauridsen J. Does the number of choice sets matter? Results from a web survey applying a discrete choice experiment. Health economics. 2011 Mar;20(3):273-8

32) Lancsar E, Fiebig DG, Hole AR. Discrete choice experiments: a guide to model specification, estimation and software. Pharmacoeconomics. 2017 Jul;35:697-716.

33) Hauber AB, González JM, Groothuis-Oudshoorn CG, Prior T, Marshall DA, Cunningham C, IJzerman MJ, Bridges JF. Statistical methods for the analysis of discrete choice experiments:

For peer review only

a report of the ISPOR conjoint analysis good research practices task force. Value in health. 2016 Jun 1;19(4):300-15.

34) Lancsar E, Louviere J. Conducting discrete choice experiments to inform healthcare decision making: a user's guide. Pharmacoeconomics. 2008 Aug;26:661-77.

35) Bridges, J.F., Mohamed, A.F., Finnern, H.W., Woehl, A. and Hauber, A.B., 2012. Patients' preferences for treatment outcomes for advanced non-small cell lung cancer: a conjoint analysis. Lung Cancer, 77(1), pp.224-231.

36) Hofheinz, R., Clouth, J., Borchardt-Wagner, J., Wagner, U., Weidling, E., Jen, M.H. and Brück, P., 2016. Patient preferences for palliative treatment of locally advanced or metastatic gastric cancer and adenocarcinoma of the gastroesophageal junction: a choice-based conjoint analysis study from Germany. *BMC cancer*, *16*, pp.1-9.

37) Ivanova, J., Hess, L.M., Garcia-Horton, V., Graham, S., Liu, X., Zhu, Y. and Nicol, S., 2019. Patient and oncologist preferences for the treatment of adults with advanced soft tissue sarcoma: a discrete choice experiment. *The Patient-Patient-Centered Outcomes Research*, *12*, pp.393-404.

38) Bottomley A, Jones D, Claassens L. Patient-reported outcomes: assessment and current perspectives of the guidelines of the Food and Drug Administration and the reflection paper of the European Medicines Agency. European Journal of Cancer. 2009 Feb 1;45(3):347-53.

39) Bouvy JC, Cowie L, Lovett R, Morrison D, Livingstone H, Crabb N. Use of patient preference studies in HTA decision making: a NICE perspective. The Patient-Patient-Centered Outcomes Research. 2020 Apr;13(2):145-9.

40) Harrison M, Milbers K, Hudson M, Bansback N. Do patients and health care providers have discordant preferences about which aspects of treatments matter most? Evidence from a systematic review of discrete choice experiments. BMJ open. 2017 May 1;7(5):e014719.

For peer review only

41) Gao JP, Jin YH, Yu SF, Wu WF, Han SF. Evaluate the effectiveness of breast cancer decision aids: A systematic review and meta-analysis of randomize clinical trails. Nursing Open. 2021 Sep;8(5):2091-104.

42) Dowsey MM, Scott A, Nelson EA, Li J, Sundararajan V, Nikpour M, Choong PF. Using discrete choice experiments as a decision aid in total knee arthroplasty: study protocol for a randomised controlled trial. Trials. 2016 Dec;17(1):1-0.

43) Hazlewood GS, Marshall DA, Barber CE, Li LC, Barnabe C, Bykerk V, Tugwell P, Hull PM, Bansback N. Using a discrete-choice experiment in a decision aid to nudge patients towards value-concordant treatment choices in rheumatoid arthritis: a proof-of-concept study. Patient preference and adherence. 2020;14:829.

44) Loría-Rebolledo LE, Ryan M, Bond C, Porteous T, Murchie P, Adam R. Using a discrete choice experiment to develop a decision aid tool to inform the management of persistent pain in pharmacy: a protocol for a randomised feasibility study. BMJ open. 2022 Sep 1;12(9):e066379.

45) Schwartz CE, Merriman MP, Reed GW, Hammes BJ. Measuring patient treatment preferences in end-of-life care research: applications for advance care planning interventions and response shift research. Journal of Palliative Medicine. 2004 Apr 1;7(2):233-45.

46) Cancer Research UK. Breast cancer incidence by age. Breast cancer incidence (invasive) statistics. 2021. Available from: https://www.cancerresearchuk.org/health-professional/cancer-statistics/statistics-by-cancer-type/breast-cancer/incidence-invasive#heading-One. Last accessed 11th November 2022.

47) Meropol, N.J., Egleston, B.L., Buzaglo, J.S., Benson III, A.B., Cegala, D.J., Diefenbach, M.A., Fleisher, L., Miller, S.M., Sulmasy, D.P., Weinfurt, K.P. and CONNECT Study

For peer review only

Research Group, 2008. Cancer patient preferences for quality and length of life. Cancer, 113(12), pp.3459-3466.

48) Bröckelmann PJ, McMullen S, Wilson JB, Mueller K, Goring S, Stamatoullas A, Zagadailov E, Gautam A, Huebner D, Dalal M, Illidge T. Patient and physician preferences for first-line treatment of classical Hodgkin lymphoma in Germany, France and the United Kingdom. British Journal of Haematology. 2019 Jan;184(2):202-14.

For peer review only

Supporting Information

S1 File. Qualitative Methods

S2 File. Additional Tables

S3 File. Additional Figures

For peer review only

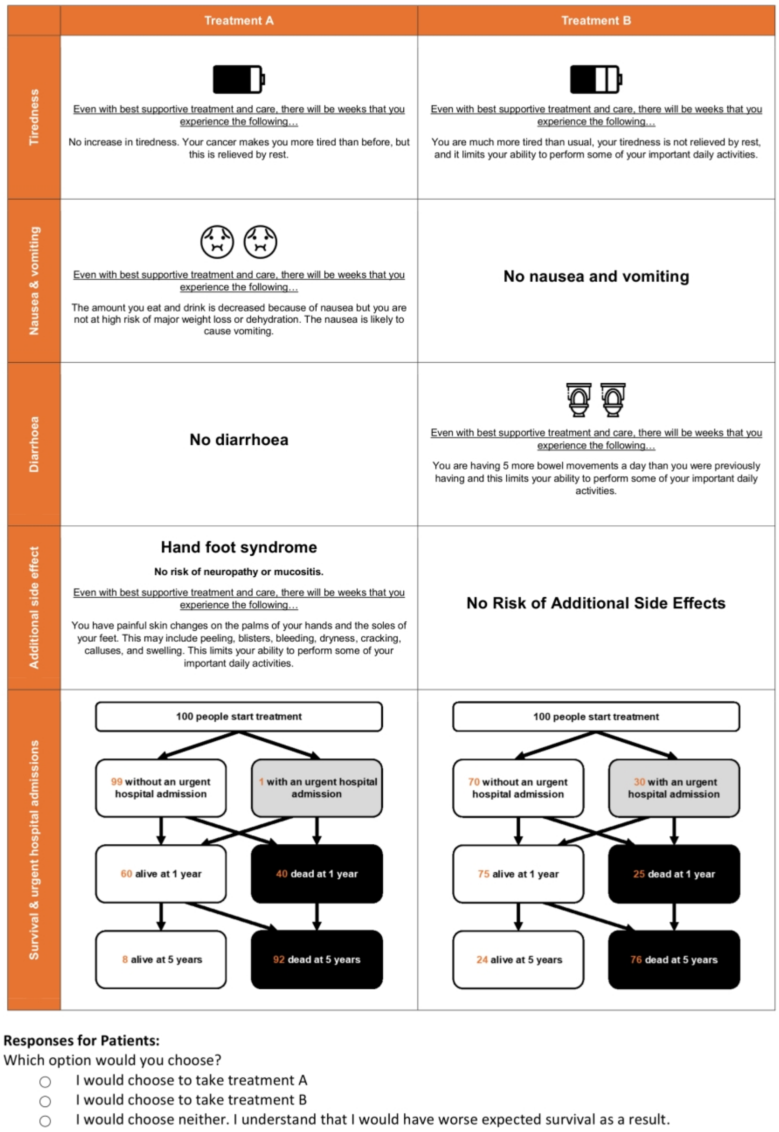

Figure 1: Example of DCE choice task (Warm-up task)

296x419mm (200 x 200 DPI)

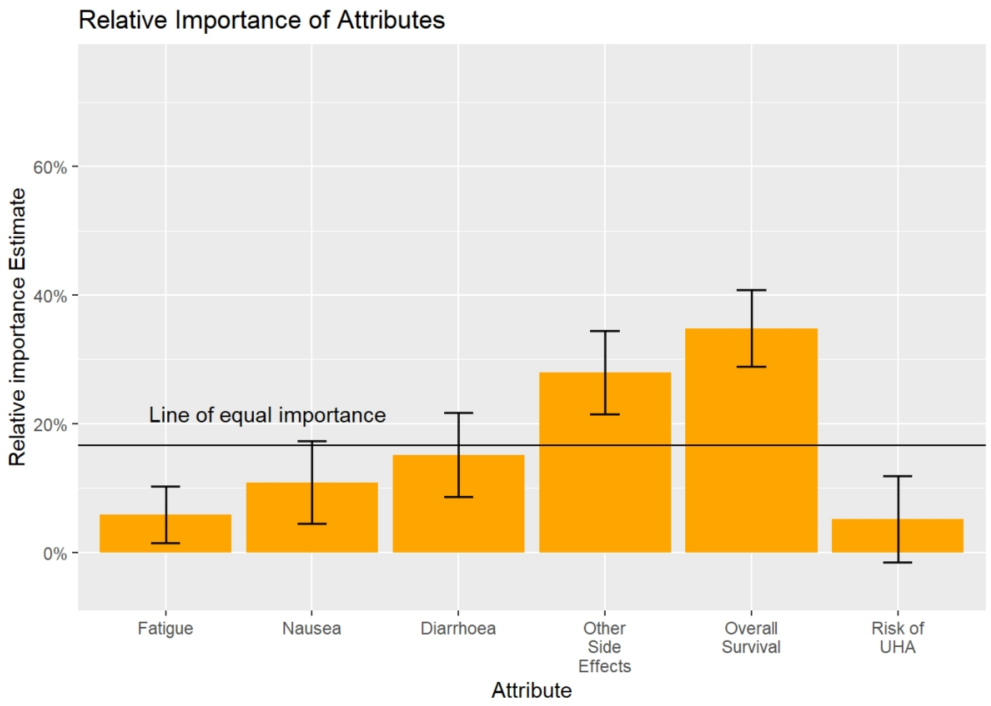

Figure 2: Relative Importance of Attributes

449x320mm (76 x 76 DPI)

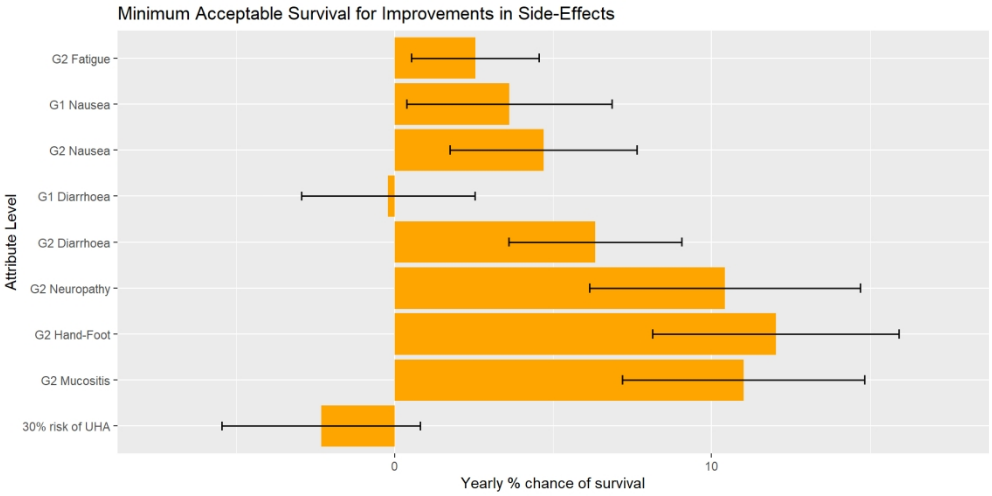

Figure 3: Minimum acceptable survival to Avoid Side effects

641x320mm (76 x 76 DPI)

Women's preferences for overall survival versus avoiding side effects in the treatment of metastatic breast cancer: a discrete choice experiment

Supporting Information 1

Qualitative Methods

Qualitative Literature Review

Embase and Medline were searched using the Ovid search engine. We aimed to identify literature which explored the patient perspective of cancer and the associated treatments. Search terms were designed to identify studies that (1) involved interviews/focus groups (2) explored patient attitudes/perspectives (3) focused on advanced or locally advanced cancer. We included all metastatic cancers given the scarcity of metastatic breast cancer-specific literature.

The search identified 434 results. Abstracts were screened and papers were excluded if they didn't reflect the underlying motivation of the search strategy. Studies were also excluded if: they focussed on an intervention which was not clinically supported or was not medicine (e.g., alternative medicine and exercise respectively); the study focus was seldom relevant to breast cancer (e.g., breathing complications brought on lung tumours). After abstract screening 83 studies remained after which 5 additional studies were excluded after reading beyond the abstract. The remaining papers were evaluated and findings which offered insight into determinants of a patient's quality of life or preference for treatment were identified. Findings were compiled and condensed into a report summarising what the available research to date suggested determining patient preferences and well-being.

Pain was among the most prominent topics of discussion. Respondents who had experienced cancer pain identified it as the most disturbing and limiting symptom of their illness (Luoma and Hakamies-Blomqvist, 2004). Patients with pain often reported extreme negative emotions (Lewis et al, 2015), loss of independence (Gibbins et al, 2014), and a desire for assisted death (Koffman et al, 2008). Other frequently explored topics included physical functioning and mobility which, as concepts, are closely linked to pain (Wilson et al, 2005). The symptoms of disease and the side effects of treatment which led to degraded physical functioning levels were identified as substantial barriers to a patient's ability to live a normal life (Gibbins et al, 2014). Extreme degradation of mobility leads to increased dependence on loved ones and carers which can create a strong sense of burden (Mak, and Elwyn, 2005). Cognitive functioning also appears to have been a topic of interest for qualitative researchers. Although cognitive functioning appears to have been a significant area of interest, many metastatic breast cancer patients rarely had symptoms, when they did, they presented as secondary disturbances or anxieties (Luoma and Hakamies-Blomqvist, 2004). Patients were willing to take medications which were associated with drowsiness to alleviate symptoms of pain (Check et al, 2017). This is evidence that patients already accept trade-offs between symptoms when considering treatments. Evidence of similar trade-offs was also found between: hot flushes and mode of administration (Fallowfield et al, 2005), expected survival and physical functioning (Check et al, 2017), and expected survival against the collective side effects of chemotherapy (Etkind et al, 2017). Evidence of trade-offs between symptoms and side effects tells us something about the importance of those toxicities, but more importantly, helps to validate the decisional context we use to frame our DCE survey questions. Other themes which featured heavily in the literature were the topics of survival, fatigue, and mode of administration, all of which are discussed in more detail in section 4 of this paper.

DCE Literature Review

The benefits of reviewing DCEs with similar motivations to our study are twofold. Firstly, they can offer insight into the importance of some of the treatment factors which we would be considering. Secondly, DCEs often employ rigorous qualitative processes and their choice of attributes is likely to

For peer review only

be of interest because their selection implicitly suggests significance. In the context of a cancer treatment DCE an attribute would be a feature of treatment which has the potential to vary between competing hypothetical treatments in a choice task. Embase and Medline were searched for DCE studies relating to patient preference for metastatic cancer treatments[1]. Search terms designed to identify DCEs mirrored those first used by Ryan and Gerard (2003). We also reincorporated the search terms used to identify metastatic cancer studies used in the qualitative literature review. Once again preliminary searches revealed that there was an insufficient body of publications to focus on metastatic breast cancer studies alone. 128 unique studies were identified in total. After screening the abstracts 60 papers met the eligibility criteria. There were 16 instances where two studies reported the results from the same DCE, in these instances the most recent publication was selected. 44 studies were identified as meeting all the criteria. Once the papers were identified work began to analyse the attributes used by the studies. The WP produced 2 key outputs of interest (1) an outline of the types of attributes used in similar past DCEs and (2) their relative importance.

Attributes were grouped into categories with similar motives. The table below outlines the attribute categories which featured in more than one DCE. There were instances where one DCE contained more than one attribute which could fit into the same category, in which instance only one was counted.

Table 1 Frequency of attribute categories included in the DCE literature review

**Attribute Category Frequency**	**n**
Administration	12
Progression Free Survival	12
Cost	8
Overall Survival	8
Pain	7
Fatigue	5
Gastrointestinal Perforation	3
Kidneys	3
Skin	3
Teeth/jaw	3
Adverse Events	2
Bone Metastases	2
Diarrhoea	2
Hospitalisation	2
Immunosuppression	2
Nausea	2
Neuropathy	2
Response rates	2
Self-care	2

Relative preference weights are measures of the importance of attributes relative to competing attributes and are conditional on the range of utility estimates for the remaining attributes (Hauber et al, 2016). A large relative preference weight suggests that an attribute has high importance in the context of the DCE's design. The selection of competing attributes, the range of levels for the

[1] the number metastatic breast cancer specific studies identified in preliminary searches were insufficient to justify their own review

For peer review only

attribute and its competitors, and framing effects (Howard and Salkeld, 2009) all determine the scale of a relative preference weight. Nevertheless, underlying preference is still a key determinant of relative preference weights and, if the considerations are accounted for, valuable inferences are possible. When making comparisons between DCEs differing study designs should be considered including decisional context, the motivations of the studies, statistical methods, and sample compositions. The complexity of these comparisons means they can't be definitively relied upon, nonetheless they are useful when consolidated with additional information from other WPs.

The main finding of the DCE literature review was the prevalence of certain attributes among the DCEs, furthermore, certain attributes tended to be associated with high relative importance between DCEs. The closely related attributes of progression free survival (PFS) and overall survival (OS) were both frequently included and tended to have high relative importance, the significance of survival and the relationship between these variations will be explored in more depth in section 4 of this paper. Pain was another category of attribute which was frequently explored and tended to be associated with high relative importance, this suggests a strong preference amongst patients to minimise suffering. It is also worth noting that, many studies appeared to be interested in patients' preferences for mode of administration, although it appeared respondents often prioritised other attributes. As a final note, the relative importance of many symptoms and side effects such as fatigue, nausea and diarrhoea differed greatly between DCEs, it was here that the limitations of making deductions from the results DCEs with different objectives were most apparent.

PACE Statement Thematic Analysis

We were granted access by the Scottish Medicines Consortium (SMC) to eight PACE statements relating to metastatic breast cancer treatments. The SMC is Scotland's advisory body for medicines, as part of their drug approval process for ultra-orphan and end-of-life medicines they invite patient and clinical representatives to meetings to discuss the benefits. These are known as Patient and Clinical Engagement (PACE) meetings. PACE meetings aim to consider all available and relevant evidence regarding new medicines including factors which traditional economic evaluation tends to overlook. We identified PACE statements as a potentially useful secondary data resource for our research since their focus is on the needs of the patient. Another advantage is that PACE statements are a relatively recent innovation meaning they tend to present up-to-date information. Between Oct 2014 and Oct 2018, eight PACE meetings were convened for medicines seeking reimbursement for the treatment of metastatic breast cancer. We conducted a formal thematic analysis (Braun and Clarke, 2012) of the PACE statements which focussed on the positive and negative impacts of treatment as well the insights into patient priorities.

We were able to identify six core themes which were composed of additional sub-themes (see figure below). Themes were not mutually exclusive, meaning there is some degree of overlap between themes. Two of the themes represent what we came to understand as the core goals of patients according to the data, these were 'Ability to live a normal life' and 'Survival'; treatments were praised repeatedly by committees for their ability to improve these two outcomes. When consulting the evidence from the PACE analysis it should be considered that they are designed to consider externalities and not just the direct effect on patients. Specifically, PACE guidelines request that respondents discuss the effect of disease and treatment on the family and carers. This explains the prominence of the 'effect on close ones' theme which is often featured in the form of considering perspectives outside of the patients. Although the findings were interesting for our research, we decided to focus on the perspective of the patient. So naturally, this theme emerged. A key disadvantage of PACE statements was their tendency to talk broadly and generally about symptoms and side effects. For our research, we were interested in patients' preferences for specific symptoms and side effects, but the lack of detail meant little could be deduced about which common side effects were more troublesome than others. It should also be noted that PACE statements are rarely critical of emerging drugs. The general feeling from the PACE statements was that participants were keen to

highlight the benefits of emerging drugs. There was a positive bias that we had to consider when toxicities and benefits associated with the treatment in question were mentioned

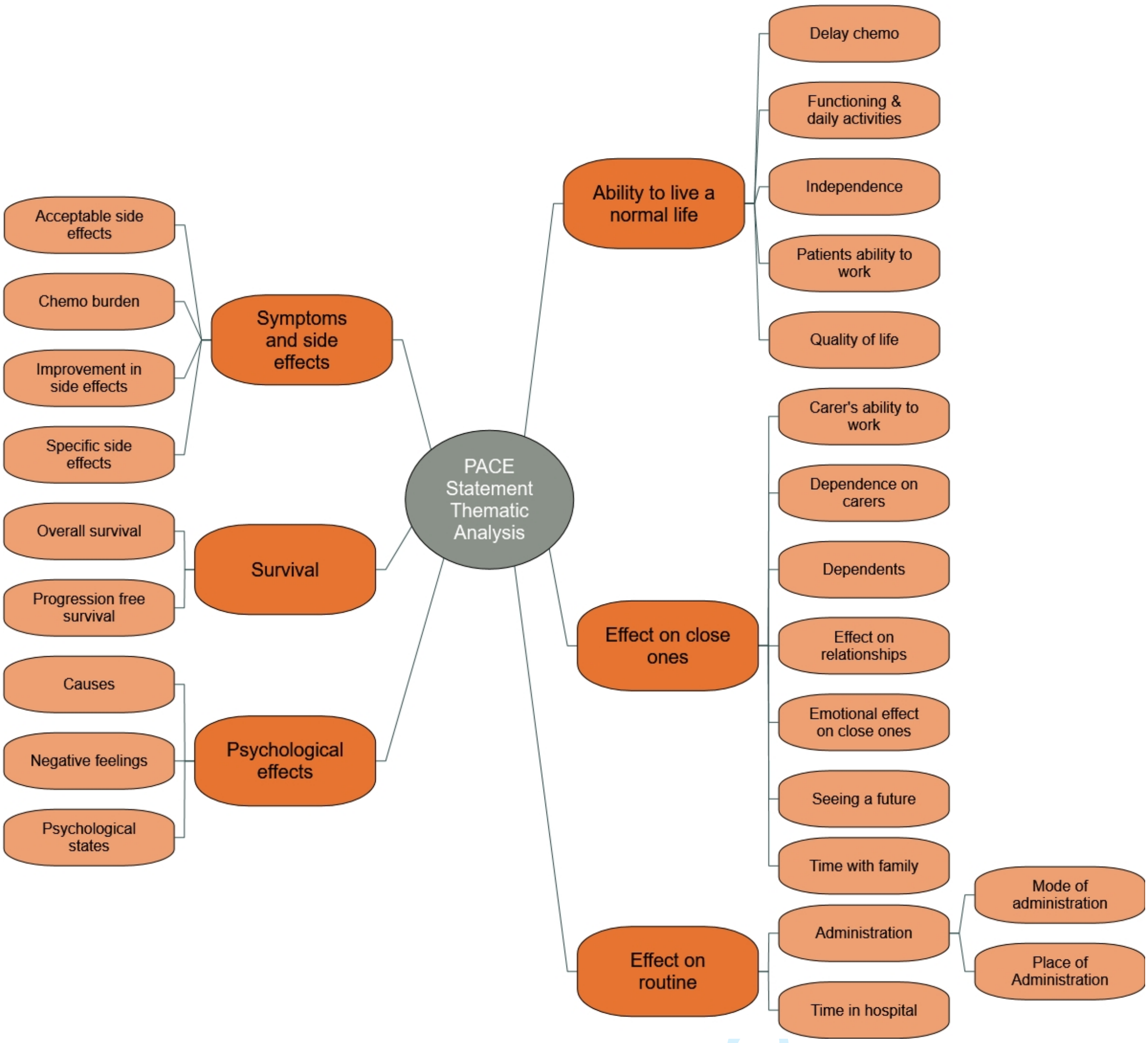

Figure 1 – Results from thematic analysis of breast cancer PACE data

Patient Interviews

The richest data from the early stages of the project emerged from the semi-structured interviews we conducted with 9 patients diagnosed with metastatic breast cancer. Women with secondary breast cases with experience of multiple treatments and who were currently living in the Lothian area were contacted by a research nurse and invited to participate in a face-to-face interview at an agreed location, either a cancer charity premises or the patient's home. We wanted to adopt a flexible strategy where we could adapt individual interviews and our broader strategies as our understanding of patient preferences and experiences developed. Grounded theory (Strauss and Corbin, 1994) is a qualitative methodology that encourages a flexible strategy, however, conventional recommendations state that interviewers should be mostly ignorant about the topic being explored so that bias does not interfere with the formulation of theories. Given that we already had considerable knowledge of the experiences of breast cancer patients, owing to ongoing research and professional experience, we instead opted to conduct interviews according to the informed grounded theory approach (Thornberg, 2012). This adaptation of the grounded theory methodology allowed us to incorporate our prior

knowledge in the traditional grounded theory approach whilst being aware of bias and remaining open to new ideas. An interview plan was formulated which provided structure whilst allowing for deviation and elaboration. The three core areas of focus were (1) patient history – patients were invited to discuss the treatments they had received and reflect on their experiences with them (2) treatment decision making – patient were asked how they remember decisions about treatment being and to reflect on the extent of their own involvement (3) experience with treatment and disease – patients were asked to reflect on their lived experience of their disease and their treatment and how it affected them.

To summarise the broader findings: There was a general attitude that more treatment was generally better and that listening to the advice of health professionals is the best thing one can do. There was a large degree of variation in terms of the specific side effects that patients' experiences and to what extent. This is likely a consequence of the wide range of secondary malignancies and the treatments received. Several patients mentioned suffering very little from symptoms and side effects since their secondary diagnosis. There was a prevailing negative attitude towards chemotherapy and its associated toxicities. The two primary goals of treatment appeared to be life extension and minimising disruption to everyday life. The interviews helped us to understand the broader goals of patients as well as their self-reported attitudes and behaviours regarding shared decision making. The richest findings however related to discussions concerning specific symptoms and side effects, evidence from these discussions will feature heavily in section 4 of this paper.

For peer review only

References

Braun, V. and Clarke, V., 2012. Thematic analysis.

Check, D. K., Park, E. M., Reeder-Hayes, K. E., Mayer, D. K., Deal, A. M., Yopp, J. M., … Hanson, L. C. (2017). Concerns underlying treatment preferences of advanced cancer patients with children. Psycho-Oncology, 26(10), 1491–1497.

Etkind, S.N., Bristowe, K., Bailey, K., Selman, L.E. and Murtagh, F.E., 2017. How does uncertainty shape patient experience in advanced illness? A secondary analysis of qualitative data. Palliative medicine, 31(2), pp.171-180.

Gibbins, J., Bhatia, R., Forbes, K., & Reid, C. M. (2014). What do patients with advanced incurable cancer want from the management of their pain? A qualitative study. Palliative Medicine, 28(1), 71–78.

Hauber, A.B., González, J.M., Groothuis-Oudshoorn, C.G., Prior, T., Marshall, D.A., Cunningham, C., IJzerman, M.J. and Bridges, J.F., 2016. Statistical methods for the analysis of discrete choice experiments: a report of the ISPOR Conjoint Analysis Good Research Practices Task Force. Value in health, 19(4), pp.300-315.

Howard, K. and Salkeld, G., 2009. Does attribute framing in discrete choice experiments influence willingness to pay? Results from a discrete choice experiment in screening for colorectal cancer. Value in health, 12(2), pp.354-363.

Koffman, J., Morgan, M., Edmonds, P., Speck, P., & Higginson, I. (2008). Cultural meanings of pain: A qualitative study of Black Caribbean and White British patients with advanced cancer. Palliative Medicine, 22(4), 350–359.

Lewis, S., Yee, J., Kilbreath, S. and Willis, K., 2015. A qualitative study of women's experiences of healthcare, treatment and support for metastatic breast cancer. The Breast, 24(3), pp.242-247.

Luoma, M.L. and Hakamies-Blomqvist, L., 2004. The meaning of quality of life in patients being treated for advanced breast cancer: a qualitative study. Psycho-Oncology: Journal of the Psychological, Social and Behavioral Dimensions of Cancer, 13(10), pp.729-739.

Mak, Y.Y.W. and Elwyn, G., 2005. Voices of the terminally ill: uncovering the meaning of desire for euthanasia. Palliative medicine, 19(4), pp.343-350.

Ryan, M. and Gerard, K., 2003. Using discrete choice experiments to value health care programmes: current practice and. Applied health economics and health policy, 2(1), pp.55-64.Hauber, A.B.,

Strauss, A. and Corbin, J., 1994. Grounded theory methodology. Handbook of qualitative research, 17, pp.273-85.

Thornberg, R., 2012. Informed grounded theory. Scandinavian Journal of Educational Research, 56(3), pp.243-259.

Wilson, K. G., Curran, D., & McPherson, C. J. (2005). A burden to others: A common source of distress for the terminally ill. Cognitive Behaviour Therapy, 34(2), 115–123

For peer review only

Supporting Information 2

Table A1 Presentation of Side Effects Attribute Levels to Respondents

**Tiredness**	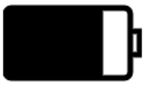<br><u>Even with best supportive treatment and care, there will be weeks that you experience the following…</u><br>No increase in tiredness. Your cancer makes you more tired than before, but this is relieved by rest.	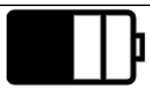<br><u>Even with best supportive treatment and care, there will be weeks that you experience the following…</u><br>You are much more tired than usual, your tiredness is not relieved by rest, and it limits your ability to perform some of your important daily activities.	
**Nausea and Vomiting**	**No nausea and vomiting**	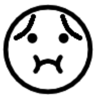<br><u>Even with best supportive treatment and care, there will be weeks that you experience the following…</u><br>You have lost your appetite due to nausea, but not enough to change the amount you eat. Your nausea may cause some vomiting.	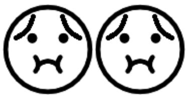<br><u>Even with best supportive treatment and care, there will be weeks that you experience the following…</u><br>The amount you eat and drink is decreased because of nausea but you are not at high risk of major weight loss or dehydration. The nausea is likely to cause vomiting.
**Diarrhoea**	**No diarrhoea**	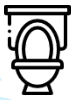<br><u>Even with best supportive treatment and care, there will be weeks that you experience the following…</u><br>You are having 2 more bowel movements a day than you were previously having.	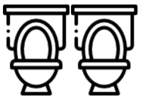<br><u>Even with best supportive treatment and care, there will be weeks that you experience the following…</u><br>You are having 5 more bowel movements a day than you were previously having and this limits your ability to perform some of your important daily activities.
**Additional Side effect**	**Peripheral neuropathy**<br>**No risk of hand foot syndrome or mucositis.**<br><u>Even with best supportive treatment and care, there will be weeks that you experience the following…</u><br>You have numbness and tingling in the feet or hands and occasionally burning, stabbing or shooting pain in affected areas. This limits your ability to perform some of your important daily activities.	**Hand foot syndrome**<br>**No risk of neuropathy or mucositis.**<br><u>Even with best supportive treatment and care, there will be weeks that you experience the following…</u><br>You have painful skin changes on the palms of your hands and the soles of your feet. This may include peeling, blisters, bleeding, dryness, cracking, calluses, and swelling. This limits your ability to perform some of your important daily activities.	**Mucositis**<br>**No risk of neuropathy or hand foot syndrome.**<br><u>Even with best supportive treatment and care, there will be weeks that you experience the following…</u><br>Your mouth becomes sore and inflamed. You have ulcers which are painful and mean you are unable to eat spicy, acidic, and crunchy foods such as crisps.
	**No risk of neuropathy, hand foot syndrome or mucositis**		

Table A2 Respondent characteristics

**Diagnosis**	**n**
Metastatic breast cancer	72
Primary breast cancer	33
**Gender**	
Female	105
Male	0
**Age**	
30-39	8
40-49	19
50-59	47
60-69	25
70-79	6

For peer review only

Table A3 Multinomial Results – Main Specification

		Estimate	p	95% CI Lower bound	95% CI Upper bound	Relative attribute importance	Minimum acceptable survival
Alternative Specific Constant	Treatment	0.9598	0.0006	0.4136	1.5060	-	-
Fatigue	Grade 2 fatigue	-0.2899	0.0089	-0.5073	-0.0726	0.0658	2.8017
Nausea	Grade 1 nausea	-0.3070	0.1021	-0.6750	0.0610	0.0951	2.9665 N.S.
	Grade 2 nausea	-0.4192	0.0232	-0.7811	-0.0573		4.0503
Diarrhoea	Grade 1 diarrhoea	0.0696	0.6425	-0.2242	0.3636	0.1536	-0.6734 N.S.
	Grade 2 diarrhoea	-0.6076	0.0011	-0.9715	-0.2438		5.8714
Additional side effects	Grade 2 peripheral neuropathy	-1.070	0.0000	-1.4654	-0.6748	0.2693	10.3399
	Grade 2 hand foot syndrome	-1.1873	0.0000	-1.5759	-0.7987		11.4723
	Grade 2 mucositis	-1.1264	0.0000	-1.4830	-0.7698		10.8842
Overall survival	Annual probability of survival	0.1035	0.0000	0.0764	0.1305	0.3521	-
Urgent Hospital Admission	Probability of urgent hospital admission in the first year of treatment	0.0097	0.0589	-0.0004	0.0198	0.0640 N.S.	-2.8223 N.S.(for 30% level)
**Model statistics**							
Number of individuals	105						
Observations	601						
Log likelihood	-431.59						
Bayesian info criterion	933.5637						

N.S. not significant

Table A4 Multinomial Results – Excluding Treat variable and paramatarising Overall Survival as Dummy Variables

Attribute	Level	Estimate	p	95% CI Lower bound	95% CI Upper bound
Fatigue	Grade 2 fatigue	-0.2887	0.0136	-0.5179	-0.0595
Nausea	Grade 1 nausea	-0.3084	0.1080	-0.6844	0.0677
	Grade 2 nausea	-0.4194	0.0232	-0.7814	-0.0574
Diarrhoea	Grade 1 diarrhoea	0.0668	0.6959	-0.2682	0.4019
	Grade 2 diarrhoea	-0.6036	0.0066	-1.0391	-0.1680
Additional side effects	Grade 2 peripheral neuropathy	-1.0758	0.0000	-1.5941	-0.5576
	Grade 2 hand foot syndrome	-1.1897	0.0000	-1.6034	-0.7761
	Grade 2 mucositis	-1.1269	0.0000	-1.4844	-0.7694
Overall survival (Annual probability of survival)	60%	2.5175	0.0000	1.9034	3.1316
	65%	3.0212	0.0000	2.2538	3.7887
	75%	4.0641	0.0000	3.2953	4.8329
Urgent Hospital Admission	Probability of urgent hospital admission in the first year of treatment	0.0100	0.2467	-0.0069	0.0268
**Model statistics**					
Number of individuals	105				
Observations	601				
Log likelihood	-431.59				
Bayesian info criterion	939.96				

Women's preferences for overall survival versus avoiding side effects in the treatment of metastatic breast cancer: a discrete choice experiment

Supporting Information 3

Figure A1 Comparison of relative importance estimates from multinomial logit models between the entire sample and 'survival non-traders' (sample excluding survival non-traders, N=71)

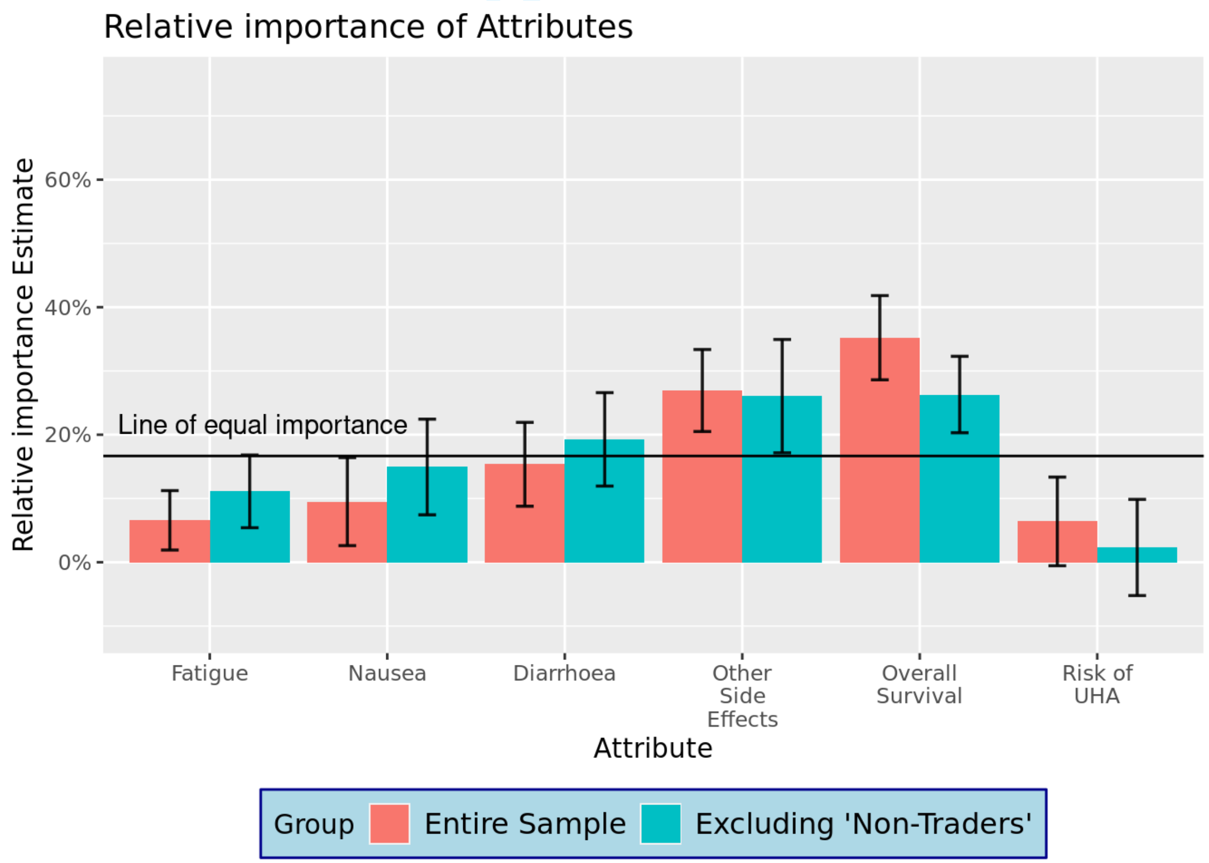

Error bars show 95% confidence interval using delta method standard errors

Figure A2 Comparison of minimum acceptable survival from multinomial logit models between the entire sample and 'survival non-traders' (sample excluding survival non-traders, N=71)

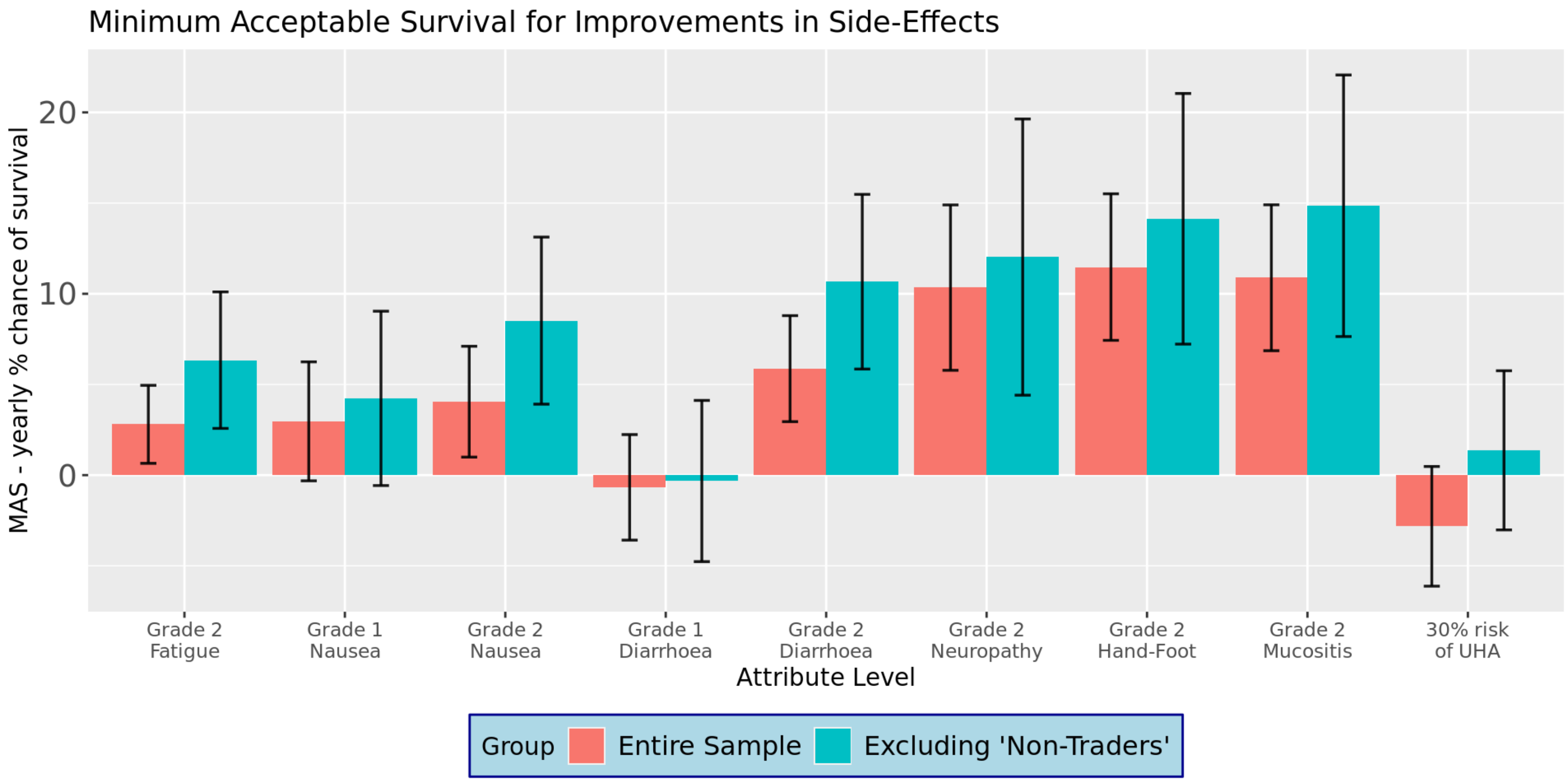

Error bars show 95% confidence interval using delta method standard errors

Figure A3 Comparison of Relative importance estimates from multinomial logit models between the metastatic breast cancer sample (N=72) and the primary breast cancer sample (N=33)

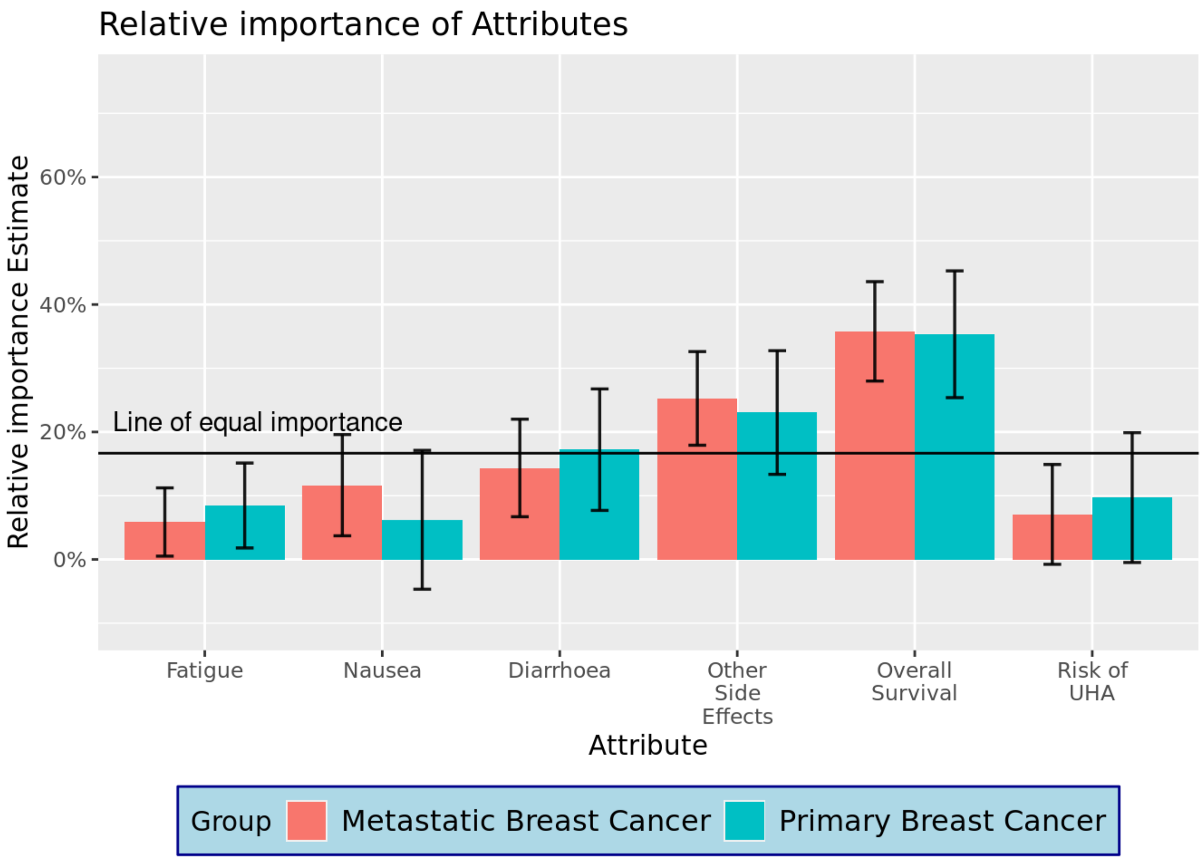

Error bars show 95% confidence interval using delta method standard errors

Figure A4 Comparison of Relative importance Estimates Between the Metastatic Breast Cancer Sample (N=72) and the Primary Breast Cancer Sample (N=33)

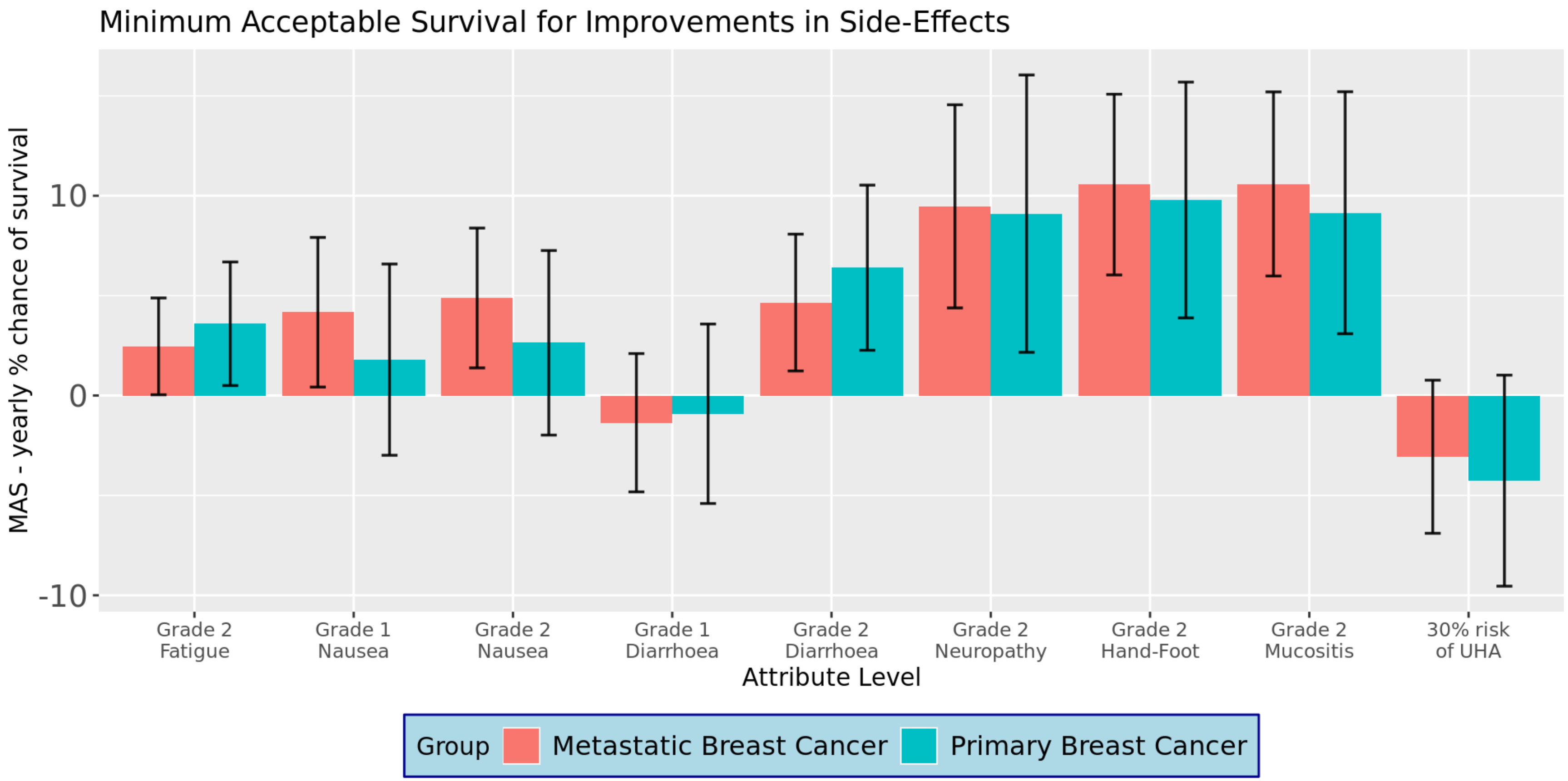

Error bars show 95% confidence interval using delta method standard errors

BMJ Open

Trade-offs between overall survival and side effects in the treatment of metastatic breast cancer: eliciting preferences of patients with primary and metastatic breast cancer using a discrete choice experiment

Journal:	*BMJ Open*
Manuscript ID	bmjopen-2023-076798.R2
Article Type:	Original research
Date Submitted by the Author:	09-Feb-2024
Complete List of Authors:	Bullen, Alistair; The University of Edinburgh Usher Institute of Population Health Sciences and Informatics, Edinburgh Clinical Trials Unit<br>Ryan, Mandy; University of Aberdeen, HERU<br>Ennis, Holly; The University of Edinburgh<br>Gray, Ewan; The University of Edinburgh<br>Loría-Rebolledo, Luis Enrique ; University of Aberdeen, Health Economics Research Unit<br>McIntyre, Morag; Western General Hospital<br>Hall, Peter; The University of Edinburgh; Western General Hospital
**Primary Subject Heading**:	Health economics
Secondary Subject Heading:	Communication, Oncology, Patient-centred medicine
Keywords:	Quality of Life, Decision Making, Surveys and Questionnaires, Breast tumours < ONCOLOGY, HEALTH ECONOMICS

SCHOLARONE™
Manuscripts

I, the Submitting Author has the right to grant and does grant on behalf of all authors of the Work (as defined in the below author licence), an exclusive licence and/or a non-exclusive licence for contributions from authors who are: i) UK Crown employees; ii) where BMJ has agreed a CC-BY licence shall apply, and/or iii) in accordance with the terms applicable for US Federal Government officers or employees acting as part of their official duties; on a worldwide, perpetual, irrevocable, royalty-free basis to BMJ Publishing Group Ltd ("BMJ") its licensees and where the relevant Journal is co-owned by BMJ to the co-owners of the Journal, to publish the Work in this journal and any other BMJ products and to exploit all rights, as set out in our licence.

The Submitting Author accepts and understands that any supply made under these terms is made by BMJ to the Submitting Author unless you are acting as an employee on behalf of your employer or a postgraduate student of an affiliated institution which is paying any applicable article publishing charge ("APC") for Open Access articles. Where the Submitting Author wishes to make the Work available on an Open Access basis (and intends to pay the relevant APC), the terms of reuse of such Open Access shall be governed by a Creative Commons licence – details of these licences and which Creative Commons licence will apply to this Work are set out in our licence referred to above.

Other than as permitted in any relevant BMJ Author's Self Archiving Policies, I confirm this Work has not been accepted for publication elsewhere, is not being considered for publication elsewhere and does not duplicate material already published. I confirm all authors consent to publication of this Work and authorise the granting of this licence.

For peer review only

Trade-offs between overall survival and side effects in the treatment of metastatic breast cancer: eliciting preferences of patients with primary and metastatic breast cancer using a discrete choice experiment

Authors Alistair J Bullen[1*]

Mandy Ryan[2]

Holly Ennis[1]

Ewan Gray[1]

Luis E Loría-Rebolledo[2]

Morag McIntyre[3]

Peter S Hall[1,3]

Affiliations

1. Edinburgh Health Economics, University of Edinburgh, Edinburgh Clinical Trials Unit, 9 Little France Rd, Edinburgh, EH16 4UX, United Kingdom
2. Health Economics Research Unit, University of Aberdeen, Polwarth Building, Foresterhill, Aberdeen, AB25 2ZD, United Kingdom
3. Edinburgh Cancer Centre, NHS Lothian, Western General Hospital, Crewe Road South, Edinburgh, EH4 2XU, United Kingdom

* Corresponding author: alistair.bullen@gcu.ac.uk

Conflicts of interest: All authors declare no conflicts of interest.

Keywords

Breast neoplasms; quality of life; decision-making, shared; patient preference; surveys and questionnaires

3954 words

For peer review only

Abstract

Objectives: There has been a recent proliferation in treatment options for patients with metastatic breast cancer. Such treatments often involve trade-offs between overall survival and side effects. Our study aims to estimate the trade-offs which could be used to inform decision-making at the individual and policy level. **Design:** We designed a discrete choice experiment (DCE) to look at preferences for avoiding severity levels of side effects when choosing treatment for metastatic breast cancer. Treatment attributes were: fatigue; nausea; diarrhoea; other side effects (peripheral neuropathy, hand foot syndrome, and mucositis)**;** urgent hospital admission and overall survival. Responses were analysed using an error component logit model. We estimated the relative importance of attributes and minimum acceptable survival for improvements in side effects. **Setting:** The DCE was completed online by UK residents with self-reported diagnoses of breast cancer. **Participants:** 105 respondents participated, of which 72 patients had metastatic breast cancer and 33 patients had primary breast cancer. **Results:** Overall survival had the largest relative importance, followed by other side effects, diarrhoea, nausea, and fatigue. Risk of urgent hospital admission was not significant. Whilst overall survival was the most important attribute, respondents were willing to forgo some absolute probability of overall survival for reductions in all Grade 2 side effects (12.02% for hand foot syndrome; 11.01% for mucositis; 10.42% for peripheral neuropathy, 6.33% for diarrhoea, and 3.62% for nausea). Grade 1 side effects were not significant, suggesting respondents have a general tolerance for them.

Conclusions: Patients are willing to forgo overall survival to avoid particular severity levels of side effects. Our results have implications for data collected in research studies and can help inform person-centred care and shared decision-making.

For peer review only

Strengths and limitations of this study

- Our study employs a discrete choice experiment methodology which is capable of estimating trade-offs for metastatic breast cancer treatment in accordance with economic utility theory.
- The selection of attributes was informed by a broad selection of work packages employing qualitative methods and reviewing a of variety of literature.
- We estimated the trade-offs between overall survival and symptoms and side effects of fatigue; nausea; diarrhoea; peripheral neuropathy; hand foot syndrome, and mucositis
- We cannot include all attributes that determine choice of treatment.
- Due to recruitment difficulties we include patients with both primary and metastatic breast cancer; these patients may have different preferences.

For peer review only

Introduction

There are 35,000 people in the UK living with metastatic breast cancer (mBC) (1). mBC occurs if the cancer spreads to another part of the body at which point the cancer is usually considered incurable. The focus of treatment then shifts from curing the disease to managing it, slowing further progression and palliating symptoms. There is a dichotomy at the core of discussions surrounding treatment in this context, namely the trade-off between overall survival (OS) and the side effects patients must tolerate (2). Different treatments offer variable prospects for survival versus side effects. Treatment decisions are made more complex by the proliferation of new medicines for the treatment of mBC, ranging from cytotoxic chemotherapy to hormone therapies. Recent new additional options include immunotherapy and targeted small molecules (3).

Such developments mean that patients with breast cancer must navigate difficult decisions between complex and unfamiliar treatments (4). Greater patient involvement in decision-making is needed to allocate the treatment which best addresses their needs. Recent guidelines have emphasised the requirement for shared decision-making across the NHS (5, 6). Although shared decision-making is widely practised its implementation needs improvement, specifically regarding doctor-patient communication (7). Evidence from patient preference studies reveals trends to be considered by healthcare providers during consultations. Patient preferences are also important for the authors of healthcare guidelines that inform policy around which drugs should be provided. As a final example, they are important for developers of new cancer drugs when they provide guidance on what patients will tolerate concerning side effects for improvements in survival.

Discrete Choice Experiments (DCEs), sometimes referred to as conjoint analysis, are increasingly used to estimate patient preferences, looking at the relative importance of attributes as well as the trade-offs individuals are willing to make (8). A recent systematic review of the application of DCEs to oncology treatment identified 79 studies, with patient preferences for breast cancer (n =10, 13%) as the most common area of application (9). The review found the most common outputs were relative

For peer review only

importance of attributes and marginal rates of substitution (MRS, trade-offs) in terms of (in order of frequency): willingness to pay (WTP); minimum acceptable benefit; minimum acceptable risk; and willingness to accept non-risk for benefit and willingness to travel. Whilst clinical efficacy attributes were commonly ranked as most important, with OS and Progression Free Survival ranked most important by 90% and 30% respectively by patient samples across all cancer types, respondents were often willing to trade clinical efficacy for improvements in side effects. A similar result was found in a systematic review of patient preference studies relating to breast cancer treatment (10). These two systematic reviews identified six DCEs that assessed preferences for mBC drug treatments (11-16). These studies also show that whilst treatment efficacy (OS or PFS) is important, and often the most important factor, patients also value avoiding the side effects of different treatments (11, 14-16). Two of these mBC studies estimated the value of avoiding side effects in monetary terms (willingness to pay, a monetary measure of benefit) (13, 14). We use the DCE methodology to investigate how much absolute probability of OS people are willing to give up to avoid a particular severity level of side effects in the treatment of mBC. We refer to this as Minimum Acceptable Survival (MAS). We also focus on the severity of side effects, whereas the existing DCEs have focussed mainly on the risk of side effects, and the preferences for long-term survival. Our study is also the first to elicit preferences for the treatment of mBC in the UK; preferences across countries may differ due to cultural factors and different healthcare systems. For example, Southeast Asian attitudes to cancer management and death are known to be different to Western ones (17).

For peer review only

Methods

The DCE is a choice-based survey that quantifies preferences for alternatives (e.g. treatment options for mBC) where alternatives are described by their attributes and associated levels (18). In our DCE alternatives are treatments, attributes are treatment characteristics (e.g. survival and side effects), and levels are values associated with treatment characteristics (e.g. % chance of survival, possible levels of severity for nausea).

Defining attributes and levels

Four work packages (WPs) informed the attributes and levels: (i) a targeted literature review of qualitative literature concerning the patient experience of metastatic cancer; (ii) a targeted literature review of DCEs centred on treatments for metastatic cancer; (iii) a thematic analysis (19) of Scottish Medicine's Consortium (SMC) Patient and Clinical Engagement (PACE) statements for mBC treatments; and (iv) face-to-face interviews with patients with mBC. All work involving face-to-face patient contact was completed by a research nurse and research assistant both of whom had been trained in qualitative methods. For more information on all WPs see Supporting Information 1. The research group, consisting of breast cancer and DCE experts, considered these attributes, reducing them to a manageable number for use in the DCE framework. Attribute selection and layperson definitions were developed using think-aloud interviews with patients (20).

The final attributes and levels are shown in Table 1, with patient definitions of attributes defined in Table A1 in Supporting Information 2. Levels are intended to represent possibilities for first-line treatment following a diagnosis at Stage IV (metastatic breast cancer). Side effects were: fatigue; nausea; diarrhoea; and additional side effects (peripheral neuropathy, hand foot syndrome, and mucositis as mutually exclusive levels). Levels of side effects attributes were described using plain-language translations of the Common Terminology Criteria for Adverse Events (CTCAE) (21) criteria (Table A1). These were developed with health professionals and tested in the developmental piloting work. Following piloting with patients, and to ease understanding, fatigue was referred to as tiredness. The nausea attribute combined the corresponding CTCAE grades nausea and vomiting (since they tend to accompany one another). Attribute levels ranged from a zero level of toxicity up to Grade 2. Choice options were discussed with health professionals to ensure plausibility. During these discussions it was suggested that some background fatigue is expected for most patients; therefore Grade 1 fatigue was the minimum level of the attribute. It was also advised that in the presence of Grade 3 adverse events, treatment would be discontinued; thus, the maximum level for all adverse

For peer review only

event attributes was Grade 2. The additional side effects attribute was included to capture a broader range of side effects while limiting the number of attributes and therefore the cognitive burden of completing the choice tasks (22). It differed from competing attributes due to each level corresponding to a unique side effect, Grade 2 descriptions were used so that we could compare preferences for the equivalent highest level of the diarrhoea and nausea attributes.

Patient and Public Involvement

Patients with mBC were invited to, and participated in, interviews and in-person questionnaire piloting sessions, both of which informed the final design of the survey.

For peer review only

Table 1 Attributes and Levels for the Discrete Choice Experiment

**Attributes**	**Levels**	**Definition**	**Regression Equation Label**	**Regression Equation Preference parameter**
Fatigue*	Grade 1 Fatigue (reference level)<br>Grade 2 Fatigue	Tiredness - In this scenario your cancer will always make you more tired than you once were. But treatments can make this worse	G2_FAT	β_1
Nausea	No nausea (reference level)<br>Grade 1 Nausea<br>Grade 2 Nausea	Treatments may cause nausea and nausea may cause you to vomit.	G1_NAU<br>G2_NAU	β_2<br>β_3
Diarrhoea	No diarrhoea (reference level)<br>Grade 1 Diarrhoea<br>Grade 2 Diarrhoea	Treatments may cause diarrhoea.	G1_DIA<br>G2_DIA	β_4<br>β_5
Additional side effects	No other side effects (reference level)<br>Grade 2 Peripheral Neuropathy<br>Grade 2 Hand foot syndrome<br>Grade 2 Mucositis	A treatment may be associated with an additional side effect. These side effects include peripheral neuropathy (nerve damage), hand foot syndrome (severe skin problems), and mucositis (mouth ulcers). You can experience a maximum of one of these side effects on a given treatment.	G2_NEU<br>G2_HAN<br>G2_MUC	β_6<br>β_7<br>β_8
Overall survival	60 alive at 1 year, 8 alive at 5 years<br>65 alive at 1 year, 12 alive at 5 years<br>75 alive at 1 year, 24 alive at 5 years	How long someone lives is always uncertain but in this scenario the care team is able to tell you how many patients are expected to be alive after 1 and 5 years. They are also able to tell you how many of those who survived the first year also experienced an urgent hospital admission. A patient may, for example, have an urgent hospital admission because of a severe infection (sepsis) or because of extreme symptoms. Hospital admission and survival statistics will both be presented in a single graphic. Please imagine that the figure for urgent hospital admissions includes hospital stays which range from days to weeks.	OS	β_9
Risk of urgent hospital admission	1/100 people<br>10/100 people<br>30/100 people		UHA	β_{10}

* Following piloting, and to ease understanding, fatigue was referred to as tiredness.

For peer review only

A risk of urgent hospital admission (UHA) was included, defined as the number of people from 100 treated who would be admitted to the hospital for a UHA. The decision to make UHA a probabilistic attribute was motivated by discussions with health professionals. It was suggested that, unlike Grade 1 and Grade 2 toxicities, a treatment which guaranteed a UHA would not be offered to patients. OS was defined as the annual probability of survival, which was time constant and represented the probability of surviving in the present and future years. To account for short and long-term preferences (23) annual probability of survival was presented as frequencies at 1 and 5 years e.g. 65% translated to 65 people alive a 1 year and 12 alive at 5 years (the rounded result of 100×0.65^5). Risk is generally not well understood by the general public (**24**), therefore 1 and 5-year survival were presented alongside one another to illustrate the effects of cumulative probability to respondents. The average 1-year survival rate after diagnosis for an mBC patient is approximately 65% (25); we chose this as our central value for our annual survival rate. We used an exponential calculation for 5-year survival, rather than real-world data, to simplify the choice task to include only one risk attribute. The levels for UHA were defined following discussions with health professionals.

It was observed during piloting that some of the expected negative preference for UHA would occur due to a risk of death. Respondents often struggled to disentangle and interpret the related attributes. To isolate the effect independently from the risk of death a graphic was devised, which showed levels of both attributes. The combination of frequencies and tree diagrams has been shown to improve understanding of risks (26, 27). The first row reports the number of patients admitted to the hospital for a UHA, and the second and third show 1- and 5-year survival respectively. Frequencies for positive outcomes (no hospitalisation and survival) and negative outcomes (hospitalisation and death) were both communicated in an attempt to address framing bias (28).

Choices presented to individuals

Ngene (Choice Metrics) was used to create a set of choices from which preferences could be estimated for all possible scenarios; the design was D-efficient, which minimized the variance-

For peer review only

covariance of the measures of average preference (29). This resulted in a set of 12 choice tasks. All choices included a no-treatment option, with side effects defined as the least severe level and risk of UHA 0%. To define the opt-out level of survival respondents were asked what they perceived their chances of survival at 1 and 5 years, resulting in a 45% average level. This was consistently lower than all levels of OS with treatment and judged reasonable given survival at one year among patients with stage 4 breast cancer diagnosed in England in 2013 was between 16-43% depending on age, with a mode of 43% (30). The choice context is shown below.

The choice scenario

The scenario You are being asked to consider the decision you would make if presented with different metastatic breast cancer treatments. For each question there are only 2 treatment options. If you choose a treatment, the other treatment will not be an option to you in the future. We ask you to imagine that no other treatment options will become available to you in the future. You also have the option to choose to have no treatment. With no treatment you would experience the symptoms of your cancer; your cancer will be left to progress and you will have shorter life expectancy as a result.

The treatments Both treatments are in the form daily pills. Both treatments can treat you for the rest of your life. You would be allowed to stop treatment whenever you wished. Both treatments have different benefits and side effects.

Side effects Side effects are guaranteed. Side effects are already being managed with the best available medicines and care. You will still experience a side effect for weeks at a time.

Following developmental work, the twelve choices were divided into two blocks of six choice tasks to mitigate mental fatigue effects (31). Respondents were randomly allocated to one of the design blocks and choice tasks were presented in a randomised order. Respondents were given a warm-up choice task (Fig 1) to complete.

Figure 1: Example of DCE choice task (Warm-up task)

[Fig 1]

For peer review only

<u>Data Analysis</u>

The following utility/benefit function was estimated using Error Component Mixed Logit regression:

$$U_{in} = \beta_0 Treat_i + \beta_1 G2\_FAT_{i1} + \beta_2 G1\_NAU_{i2} + \beta_3 G2\_NAU_{i3} + \beta_4 G1\_DIA_{i4} + \beta_5 G2\_DIA_{i5} + \beta_6 G2\_NEU_{i6} + \beta_7 G2\_HAN_{i7} + \beta_8 G2\_MUC_{i8} + \beta_9 OS_{i9} + \beta_{10} UHA_{i10} + \varepsilon_{in}$$

U_{in} represents the utility for individual n for alternative i. The attribute variables are defined in Table 1. β_1 to β_8 are modelled as dummy variables, showing the value of that attribute level relative to the reference (best) level. β_9 and β_{10} are modelled as continuous variables, showing the value of a % change in OS and UHA. The signs of the β parameters indicate whether the effect of the attribute level on preference is positive or negative. All side effects preference parameters are expected to have a negative sign relative to the reference level. Respondents are expected to prefer higher OS, resulting in a positive β_9. The preference for chance of UHA, β_{10}, is expected to have a negative sign, with lower values preferred. εin represents the unobserved error component. β_0 shows the general preference for treatment over no treatment (everything else equal) with a positive sign indicating a general preference to receive treatment (everything else equal). An error component is assumed by specifying β_0 as random normally distributed, thus allowing for flexible substitution between alternatives and dropping the irrelevant alternatives assumption (32), we run 100 draws using the Halton sequence.

For peer review only

We used the parameter values to estimate the relative importance of attributes (33); this is calculated as the difference in the range of attribute's variable values. We calculate percentages from these relative ranges, obtaining a set of attribute importance values that add to 100%. We also estimate MRS in the form of MAS for improvements in side effects using the rate for 1-year OS in the calculation, estimated as $\frac{\beta_x}{-\beta_9}$. For example, $\frac{\beta_1}{-\beta_9}$ shows MAS for a reduction in side effects from

Grade 2 fatigue to Grade 1 fatigue and $\frac{\beta_4}{-\beta_9}$ shows MAS for a reduction in side effects from Grade 1 diarrhoea to no diarrhoea.

Sample and Recruitment

Calculating an optimal sample size for newly designed DCEs is problematic as it depends on the true values of the unknown parameters for which the analysis intends to estimate (34). Previous DCEs in the area of metastatic cancer of a similar design have demonstrated that reliable analysis can be performed with samples of 100 or fewer participants (35-37). We therefore aimed to recruit 100 patients as a minimum threshold.

We planned to recruit a sufficient number of people with experience of metastatic breast cancer to exceed the minimum threshold. Given the anticipated challenges of recruiting a sufficient number of people who had an mBC diagnosis, the original protocol also included the collection of responses from people who had experienced primary breast cancer. Respondents who responded that they had only a primary breast cancer were asked to imagine that they had received a secondary breast cancer diagnosis in the introductory text. The preferences of patients with mBC were compared to patients with primary breast cancer.

The DCE was administered using an online link between January and March 2020. Recruitment methods included: (i) distribution of leaflets at cancer centres and conferences; (ii) an online panel provided by Dynata; (iii) social media engagement with help from breast cancer charities; and (iv) a research nurse approaching patients directly during clinic visits and inviting them to complete the survey on a tablet device. Interviewed respondents provided informed written consent before interviews proceeded. Access to the survey was unrestricted for people who had acquired the link. Patients self-identified as having had a primary or metastatic breast cancer diagnosis at some point, being a UK resident, and 18+ years of age. Inclusion in the sample was not restricted by gender.

For peer review only

Results

The sample size was 105 (Table A2 in Supporting Information 2). All identified as female. Seventy-two respondents were patients with mBC and 33 were patients with primary breast cancer.

10 respondents did not complete all 6 choice tasks, resulting in 29 missing choice tasks. Completed choice tasks were included in the analysis. Of 601 responses to choice tasks across all participants, 38 (6.32%) were for no treatment. These were selected by 16 women, with three women always choosing the opt-out option. 32.38% (N=34) of respondents always chose the option with the highest OS. Some of these respondents may have been using a simplifying heuristic, nonetheless, we focus our analysis on the complete sample as it is not possible to distinguish respondents who are demonstrating a genuine preference and those using a simplifying heuristic. (Figures A1 and A2 in Supporting Information 3 compare analyses when excluding the 34 potential non-traders; as expected the relative importance of OS is lower and participants have a higher MAS. However, samples are too small to demonstrate statistically significant differences.)

Table 2 shows the error-component logit regression results for all respondents (Table A3 in supporting information 2 shows the results of the equivalent multinomial logit) and Fig 2 shows the relative importance of attributes. We also ran an alternative specification as multinomial logit where the overall survival attribute was dummy coded and it demonstrated a near linear relationship between effect and survival gain between the 60 and 75 levels which suggests the specification of overall survival as a constant variable is appropriate (Supporting Information 3, Table A4).

Figure 2: Relative Importance of Attributes

[Figure 2]

Error bars show 95% confidence interval using delta method standard errors

For peer review only

291 MAS estimates (Table 2, column 8 and Fig 3) show respondents' willingness to forgo OS to avoid all Grade 2 toxicities.

294 Figure 3: Minimum acceptable survival to Avoid Side effects

295 [Figure 3]

296 Error bars show 95% confidence interval using delta method standard errors

298 Results comparing patients with mBC and patients with primary breast cancer are shown in Figures A3 and A4 Supporting Information 3. The most notable difference is the estimated importance of the nausea attribute, nonetheless, there are no statistically significant differences between any of the estimates.

For peer review only

302 **Table 2 Error Component Logit**

		Estimate	p	95% CI Lower bound	95% CI Upper bound	Relative attribute importance	Minimum acceptable survival
Alternative Specific Constant	Treatment	5.1339	0.0002	2.4566	7.8112	-	-
	Standard Deviation of Treatment	4.0982	0.0001	2.0411	6.1553		
Fatigue	Grade 2 fatigue	-0.2948	0.0101	-0.5194	-0.0702	0.0590	2.5412
Nausea	Grade 1 nausea	-0.4196	0.0519	-0.8426	0.0034	0.1091	3.6178
	Grade 2 nausea	-0.5446	0.0093	-0.9550	-0.1342		4.6960
Diarrhoea	Grade 1 diarrhoea	0.0241	0.8806	-0.2898	0.3379	0.1519	-0.2074 N.S.
	Grade 2 diarrhoea	-0.7343	0.0004	-1.1384	-0.3302		6.3314
Additional side effects	Grade 2 peripheral neuropathy	-1.2087	0.0000	-1.6458	-0.7715	0.2793	10.4211
	Grade 2 hand foot syndrome	-1.3946	0.0000	-1.8404	-0.9489		12.0247
	Grade 2 mucositis	-1.2764	0.0000	-1.6668	-0.8861		11.0055
Overall survival	Annual probability of survival	0.1160	0.0000	0.0847	0.1473	0.3485	-
Urgent Hospital Admission	Probability of urgent hospital admission in the first year of treatment	0.0090	0.1068	-0.0019	0.0199	0.0522 N.S.	-2.3236 N.S.(for 30% level)
**Model statistics**							
Number of individuals	105						
Observations	601						
Log likelihood	-379.9434						
Bayesian info criterion	836.6699						

303 *N.S. not significant*

Discussion

We provide new evidence on UK women's preferences for the treatment of mBC. Respondents had a general preference for treatment, indicated by the low opt-out rates which result in a positive constant term (Treat). As expected, they preferred treatments with higher OS, in fact almost a third of the sample (32.38%) always chose the treatment option with a higher OS. All Grade 2 toxicities were significant and negative, suggesting negative preferences for these attribute levels. However, Grade 1 nausea and diarrhoea were not significant, suggesting patients are indifferent when compared to having none of these side effects There was no significant effect of UHA on respondents' choices.

The relative importance of OS exceeded all other attributes, with an overall importance score of 34.85%. The remaining relative importance was distributed accordingly: additional side effects (27.93%), diarrhoea (15.19%), nausea (10.90%), fatigue (5.90%), and risk of urgent hospital admission (5.22%). Respondents would accept a reduction in the probability of survival of 2.54% to avoid Grade 2 fatigue (and have Grade 1 fatigue). The MAS associated with levels of the additional side effects were particularly high: respondents were willing to give up 10.42%, 12.02%, and 11.01% chance of OS for total avoidance of grade 2 peripheral neuropathy, grade 2 hand foot syndrome, and grade 2 mucositis respectively. Notably, Grade 1 nausea and diarrhoea were acceptable to patients and did not significantly impact patients' choices. Thus, they were not willing to give up survival for improvements in such Grade 1 side effects. However, Grade 2 side effects were disliked and respondents were willing to forgo up to 12.02% OS to avoid such severe side effects.

Our results add to a growing literature showing that patients with breast cancer value avoiding the side effects of treatments, and are willing to forgo some level of treatment efficacy to achieve this (9,10). Directly comparing preference estimates between studies is often inappropriate as estimates only apply to the attributes and levels within the choice framework of DCE from which they are derived. Nonetheless, it is important to highlight the findings of other studies and draw comparisons where appropriate. Our results appear to align somewhat with DiBonaventura *et al's.* (11) exploration

For peer review only

of the preferences of women with mBC in the USA who also found that OS was the most important attribute. Additionally, side effects (alopecia, fatigue, neutropenia, motor neuropathy, and nausea/vomiting) and dosing regimen were also important. The remaining studies did not include attributes for overall survival but did identify statistically significant preferences for side-effect avoidance. For example, Omori *et al.* (15) explored the preferences of Japanese postmenopausal patients with HR+ breast cancer for the treatment of mBC. They conclude that women preferred treatments that extend PFS despite potential grade 2 diarrhoea. However, when diarrhoea severity increased to grade 3, patients were more willing to sacrifice PFS to avoid more frequent diarrhoea. In contrast, exploring preferences of women diagnosed with mBC in Germany, Spaich *et al* (16) concluded that severe neutropenia was the most important attribute, followed by alopecia, neuropathy and PFS. Two studies have explored the preferences of women diagnosed with mBC in the USA, estimating value in monetary terms. Lalla et al (12) found that women were willing to pay the most to avoid severe diarrhoea (US$3,894 a year), followed by avoidance of hospitalization due to infection (US$3,279), severe nausea (US$3,211) and severe peripheral neuropathy (US$2,764). MacEwan et al (13) found that women were willing to pay US$1930 per month for treatment, with US$63 per month for each 1% reduction in the risk of moderate to severe side effects. In a similar study in Thailand, Ngorsuraches and Thongkeaw (14) found respondents were willing to pay US$151.6 per month for every 1 month increase in PFS compared to US$69.8 and US$278.3 per month for every 1% decreased risk of anaemia and pneumonitis respectively.

Our results imply that treatment efficacy and OS are not the only endpoints of value to women with mBC (and indeed oncology more broadly). Furthermore, there is evidence that the CTCAE grading criteria do not scale in parallel with patients' preferences; for example, Grade 2 nausea is preferred to Grade 2 hand foot syndrome (indicated by a lower negative preference parameter). Grade 1 toxicities were not significant, suggesting they are relatively tolerable to patients (compared to having no side effects). These findings suggest that clinician-reported and objectively graded toxicities may not correspond to patients' values and support the further incorporation of Patient Reported Outcomes (PROs) and preference studies in the study of new medicines for mBC. PROs are increasingly

For peer review only

accepted by the US Food and Drug Administration (FDA) and European Medicines Agency (EMA) (38) and the National Institute for Health and Care Excellence (NICE) has begun to accept patient preference studies alongside traditional evidence such as cost per QALY (39).

Our study has focused on the preferences of patients. Given that health professionals often make treatment decisions/recommendations for patients, a fruitful area for future research is to compare the preferences of patients and doctors. Current research suggests that it is common for there to be a mismatch in the preferences of patients and healthcare providers (40). Given health professionals possess greater information on treatments and patients possess private information on their values and priorities, Decision Aid Tools (DAT) can help understand and bridge this mismatch as part of shared decision-making. The focus of such DATs within breast cancer has been on the detection and prevention of early breast cancer (41). The work presented in this paper contributes to the groundwork for the use of a DCE as a DAT to promote shared decision-making and person-centred care. A limited number of studies have adapted DCEs into DATs: Dowsey *et al.* (42) used a DCE as part of a decision aid for patients undergoing total knee arthroplasty; Hazlewood *et al*. (43) evaluated a proof-of-concept DAT for patients with early rheumatoid arthritis, which included a DCE to assist respondents in choosing initial treatment and Loria-Rebolledo *et al*. (44) are exploring the use of DCEs to estimate preferences at the individual level for use in a shared decision-making setting.

There are limitations to this study. Firstly, the sample size was small, and we were required to supplement the mBC patient sample with primary breast cancer who were asked to imagine a secondary diagnosis. Although the analysis did not present large enough differences in preferences to suggest this meaningfully affected results, a larger sample would allow the possibility of preference heterogeneity to be extensively explored. Preferences, trade-offs and willingness to avoid particular side effects may be influenced by many factors. One potential area for future research is understanding the dynamics of treatment preferences and response shift. This may be particularly important for end-of-life care, which patients with mBC may face (45). Other factors that may influence preferences include specific cancer diagnosis, location of metastases, multiple diagnoses,

For peer review only

and treatment experience. Future research should collect data on the characteristics of respondents which could be used to explore preference heterogeneity. Secondly, national data indicates that the highest incidence of new breast cancer cases (any stage) for women between 2015 and 2017 was aged 60-69 (46), suggesting our sample is younger with the largest group aged 50-59. A 2008 survey in the United States found a stronger preference for quality of life than quantity of life among patients with cancer (47), if this effect exists in our population the preference weights may be positively skewed. Thirdly, the argument could be made that the description of how side effects are experienced in the choice scenario may be difficult for patients to understand. The decision to focus on symptom severity and to avoid clear definitions of symptom frequency relating to side effects was made to alleviate the cognitive burden of the task by simplifying the information presented. We opted to represent uncertainty by suggesting that treatments were indefinite and side effects would therefore be indefinitely experienced “for weeks at a time”. Some would argue that in doing so we forgo a degree of clarity of interpretation for respondents and consequently the results of the study. Fourthly, we simplified the choice task to include only one risk attribute, we used an exponential function to estimate the five-year survival rate. Future research could include two attributes, one and five-year survival, with the latter based on real data. Preferences for short and long-term survival could then be estimated. Fifthly, in defining the no treatment option, the level for OS was defined as the mean value from women’s perceived OS without treatment. Results may have differed if we informed respondents of their chance of survival without treatment. Furthermore, the baseline levels for side-effect attributes were assumed to be the minimum possible realistic levels, however, respondents may have implicitly considered unique individual baselines based on lived experience. The interpretation of the no treatment option may have differed between respondents and may have caused some attribute levels to appear acceptable for respondents who considered them to be the same as baseline, potentially dampening their overall effect within the sample. Results may be more precise if we estimated preferences within a more sophisticated design which adjusted for respondents’ baseline levels. Finally, whilst the insignificance of the risk of UHA may be a genuine preference, the result may also reflect a difficulty in understanding this attribute. Despite low relative importance, similar

For peer review only

attributes are significant in other metastatic cancer DCEs, however, the attribute levels are more severe (37, 48). Future work should explore explaining this attribute.

In conclusion, our results provide evidence that patients are willing to give up some survival benefits to avoid severe levels of side effects. Future therapeutic studies should ensure such data is collected to ensure that the patient can make an informed decision when making treatment decisions. Future research should explore using such information within a shared decision-making framework.

For peer review only

Data Sharing

The dataset used for this analysis is available from the University of Edinburgh Datashare, https://datashare.ed.ac.uk/handle/10283/4436.

Funding

This research was supported by a charitable grant from the Edinburgh and Lothians Health Foundation (Scottish Registered Charity No: SC007342). Mandy Ryan and Luis E Loría-Rebolledo are funded by the University of Aberdeen and the Chief Scientist Office of the Scottish Government Health and Social Care Directorates. The funders had no role in considering the study design or in the collection, analysis, or interpretation of data, the writing of the paper, or the decision to submit the article for publication.

For peer review only

Ethics

The protocol was approved by the National Health Service (NHS) North of Scotland Research Ethics Committee (REC ref: 19/NS/0066). All participants provided informed consent for every stage of the research.

Author Contributions

PH, EG, HE and MR contributed to the conceptualisation of the project and funding application. All authors contributed to the design of the study. AB developed the DCE, with experimental design support from MR. AB and MM collected the data. AB conducted the statistical analysis with support from LL. All authors contributed to the interpretation of the data as well as the drafting and revision of the manuscript. All authors gave final approval and agreed to be accountable for all aspects of the work.

Acknowledgements

We thank all the patients and volunteers who agreed to participate in this study and the study contributors.

For peer review only

References

1) Feinmann J. Number of UK patients with metastatic breast cancer isn't known. BMJ 2017;359:j5205

2) Brom L, Pasman HR, Widdershoven GA, van der Vorst MJ, Reijneveld JC, Postma TJ, Onwuteaka-Philipsen BD. Patients' preferences for participation in treatment decision-making at the end of life: qualitative interviews with advanced cancer patients. PloS one. 2014 Jun 25;9(6):e100435.

3) Waks AG, Winer EP. Breast cancer treatment: a review. Jama. 2019 Jan 22;321(3):288-300.

4) Mosher CE, Johnson C, Dickler M, Norton L, Massie MJ, DuHamel K. Living with metastatic breast cancer: a qualitative analysis of physical, psychological, and social sequelae. The breast journal. 2013 May;19(3):285-92.

5) Scottish Government. What Works to Support and Promote Shared Decision Making: A synthesis of recent evidence. 2019. Available from: https://www.gov.scot/publications/works-support-promote-shared-decision-making-synthesis-recent-evidence/pages/3/. Last accessed 7th March 2022.

6) NICE. Shared Decision Making Collaborative – An Action Plan. 2016. Available from: https://www.nice.org.uk/Media/Default/About/what-we-do/shared-decision-making-collaborative-action-plan.pdf. Last accessed 7th March 2022.

7) Covvey JR, Kamal KM, Gorse EE, Mehta Z, Dhumal T, Heidari E, Rao D, Zacker C. Barriers and facilitators to shared decision-making in oncology: a systematic review of the literature. Supportive Care in Cancer. 2019 May;27(5):1613-37.

8) Soekhai V, de Bekker-Grob EW, Ellis AR, Vass CM. Discrete choice experiments in health economics: past, present and future. Pharmacoeconomics. 2019 Feb;37(2):201-26.

For peer review only

9) Collacott H, Soekhai V, Thomas C, Brooks A, Brookes E, Lo R, Mulnick S, Heidenreich S. A systematic review of discrete choice experiments in oncology treatments. The Patient-Patient-Centered Outcomes Research. 2021 Nov;14(6):775-90.

10) Guerra RL, Castaneda L, de Albuquerque RD, Ferreira CB, Correa FD, Fernandes RR, de Almeida LM. Patient preferences for breast cancer treatment interventions: a systematic review of discrete choice experiments. The Patient-Patient-Centered Outcomes Research. 2019 Dec;12(6):559-69.

11) DiBonaventura M, Copher R, Basurto E, Faria C, Lorenzo R. Patient preferences and treatment adherence among women diagnosed with metastatic breast cancer. American Health & Drug Benefits. 2014 Oct;7(7):386.

12) Lalla D, Carlton R, Santos E, Bramley T, D'Souza A. Willingness to pay to avoid metastatic breast cancer treatment side effects: results from a conjoint analysis. Springerplus. 2014 Dec;3(1):1-7.

13) MacEwan JP, Doctor J, Mulligan K, May SG, Batt K, Zacker C, Lakdawalla D, Goldman D. The value of progression-free survival in metastatic breast cancer: results from a survey of patients and providers. MDM policy & practice. 2019 Jun;4(1):2381468319855386.

14) Ngorsuraches S, Thongkeaw K. Patients' preferences and willingness-to-pay for postmenopausal hormone receptor-positive, HER2-negative advanced breast cancer treatments after failure of standard treatments. Springerplus. 2015 Dec;4(1):1-1.

15) Omori Y, Enatsu S, Cai Z, Ishiguro H. Patients' preferences for postmenopausal hormone receptor-positive, human epidermal growth factor receptor 2-negative advanced breast cancer treatments in Japan. Breast Cancer. 2019 Sep;26(5):652-62.

For peer review only

493 16) Spaich S, Kinder J, Hetjens S, Fuxius S, Gerhardt A, Sütterlin M. Patient preferences regarding chemotherapy in metastatic breast cancer—a conjoint analysis for common taxanes. Frontiers in oncology. 2018 Nov 21;8:535.

496 17) Ong KJ, Back MF, Lu JJ, Shakespeare TS, Wynne CJ. Cultural attitudes to cancer management in traditional South-East Asian patients. Australasian radiology. 2002 Dec;46(4):370-4.

499 18) Lancaster KJ. A new approach to consumer theory. Journal of political economy. 1966 Apr 1;74(2):132-57.

501 19) Braun V, Clarke V. Thematic analysis. American Psychological Association; 2012.

502 20) Cheraghi-Sohi S, Bower P, Mead N, McDonald R, Whalley D, Roland M. Making sense of patient priorities: applying discrete choice methods in primary care using 'think aloud' technique. Family practice. 2007 Jun 1;24(3):276-82.

505 21) National Cancer Institute (U.S.). Adverse events/CTCAE. 2021. Available: https://ctep.cancer.gov/protocoldevelopment/electronic_applications/ctc.htm. Last accessed 7th March 2022.

508 22) Bridges JF, Hauber AB, Marshall D, Lloyd A, Prosser LA, Regier DA, Johnson FR, Mauskopf J. Conjoint analysis applications in health—a checklist: a report of the ISPOR Good Research Practices for Conjoint Analysis Task Force. Value in health. 2011 Jun 1;14(4):403-13.

512 23) Chao LW, Szrek H, Pereira NS, Pauly MV. Time preference and its relationship with age, health, and survival probability. Judgment and decision making. 2009 Feb 1;4(1):1.

514 24) Peters E, Hibbard J, Slovic P, Dieckmann N. Numeracy skill and the communication, comprehension, and use of risk-benefit information. Health affairs. 2007 May;26(3):741-8.

For peer review only

25) Cancer Research UK. Breast survival by stage at diagnosis. Breast cancer survival statistics. 2019. Available from: https://www.cancerresearchuk.org/health-professional/cancer-statistics/statistics-by-cancer-type/breast-cancer/survival#heading-Three. Last accessed 11th November 2022.

26) Kurz-Milcke E, Gigerenzer G, Martignon L. Transparency in risk communication: graphical and analog tools. Annals of the New York Academy of Sciences. 2008 Apr;1128(1):18-28.

27) Spiegelhalter D, Pearson M, Short I. Visualizing uncertainty about the future. science. 2011 Sep 9;333(6048):1393-400.

28) Howard K, Salkeld G. Does attribute framing in discrete choice experiments influence willingness to pay? Results from a discrete choice experiment in screening for colorectal cancer. Value in health. 2009 Mar;12(2):354-63.

29) ChoiceMetrics.NGENE (1.2). 2018. [NGENE]. [Accessed 20 January 2020].

30) Office for National Statistics. Cancer survival in England: adult, stage at diagnosis and childhood - patients followed up to 2018. 2019. Available from: https://www.ons.gov.uk/peoplepopulationandcommunity/healthandsocialcare/conditionsanddiseases/bulletins/cancersurvivalinengland/stageatdiagnosisandchildhoodpatientsfollowedupto2018. Last accessed 21st January 2021.

31) Bech M, Kjaer T, Lauridsen J. Does the number of choice sets matter? Results from a web survey applying a discrete choice experiment. Health economics. 2011 Mar;20(3):273-8

32) Lancsar E, Fiebig DG, Hole AR. Discrete choice experiments: a guide to model specification, estimation and software. Pharmacoeconomics. 2017 Jul;35:697-716.

33) Hauber AB, González JM, Groothuis-Oudshoorn CG, Prior T, Marshall DA, Cunningham C, IJzerman MJ, Bridges JF. Statistical methods for the analysis of discrete choice experiments:

For peer review only

a report of the ISPOR conjoint analysis good research practices task force. Value in health. 2016 Jun 1;19(4):300-15.

34) Lancsar E, Louviere J. Conducting discrete choice experiments to inform healthcare decision making: a user’s guide. Pharmacoeconomics. 2008 Aug;26:661-77.

35) Bridges, J.F., Mohamed, A.F., Finnern, H.W., Woehl, A. and Hauber, A.B., 2012. Patients’ preferences for treatment outcomes for advanced non-small cell lung cancer: a conjoint analysis. Lung Cancer, 77(1), pp.224-231.

36) Hofheinz, R., Clouth, J., Borchardt-Wagner, J., Wagner, U., Weidling, E., Jen, M.H. and Brück, P., 2016. Patient preferences for palliative treatment of locally advanced or metastatic gastric cancer and adenocarcinoma of the gastroesophageal junction: a choice-based conjoint analysis study from Germany. *BMC cancer*, *16*, pp.1-9.

37) Ivanova, J., Hess, L.M., Garcia-Horton, V., Graham, S., Liu, X., Zhu, Y. and Nicol, S., 2019. Patient and oncologist preferences for the treatment of adults with advanced soft tissue sarcoma: a discrete choice experiment. *The Patient-Patient-Centered Outcomes Research*, *12*, pp.393-404.

38) Bottomley A, Jones D, Claassens L. Patient-reported outcomes: assessment and current perspectives of the guidelines of the Food and Drug Administration and the reflection paper of the European Medicines Agency. European Journal of Cancer. 2009 Feb 1;45(3):347-53.

39) Bouvy JC, Cowie L, Lovett R, Morrison D, Livingstone H, Crabb N. Use of patient preference studies in HTA decision making: a NICE perspective. The Patient-Patient-Centered Outcomes Research. 2020 Apr;13(2):145-9.

40) Harrison M, Milbers K, Hudson M, Bansback N. Do patients and health care providers have discordant preferences about which aspects of treatments matter most? Evidence from a systematic review of discrete choice experiments. BMJ open. 2017 May 1;7(5):e014719.

For peer review only

563 41) Gao JP, Jin YH, Yu SF, Wu WF, Han SF. Evaluate the effectiveness of breast cancer decision
564 aids: A systematic review and meta-analysis of randomize clinical trails. Nursing Open. 2021
565 Sep;8(5):2091-104.

566 42) Dowsey MM, Scott A, Nelson EA, Li J, Sundararajan V, Nikpour M, Choong PF. Using
567 discrete choice experiments as a decision aid in total knee arthroplasty: study protocol for a
568 randomised controlled trial. Trials. 2016 Dec;17(1):1-0.

569 43) Hazlewood GS, Marshall DA, Barber CE, Li LC, Barnabe C, Bykerk V, Tugwell P, Hull PM,
570 Bansback N. Using a discrete-choice experiment in a decision aid to nudge patients towards
571 value-concordant treatment choices in rheumatoid arthritis: a proof-of-concept study. Patient
572 preference and adherence. 2020;14:829.

573 44) Loría-Rebolledo LE, Ryan M, Bond C, Porteous T, Murchie P, Adam R. Using a discrete
574 choice experiment to develop a decision aid tool to inform the management of persistent pain
575 in pharmacy: a protocol for a randomised feasibility study. BMJ open. 2022 Sep
576 1;12(9):e066379.

577 45) Schwartz CE, Merriman MP, Reed GW, Hammes BJ. Measuring patient treatment
578 preferences in end-of-life care research: applications for advance care planning interventions
579 and response shift research. Journal of Palliative Medicine. 2004 Apr 1;7(2):233-45.
580

581 46) Cancer Research UK. Breast cancer incidence by age. Breast cancer incidence (invasive)
582 statistics. 2021. Available from: https://www.cancerresearchuk.org/health-
583 professional/cancer-statistics/statistics-by-cancer-type/breast-cancer/incidence-
584 invasive#heading-One. Last accessed 11th November 2022.

585 47) Meropol, N.J., Egleston, B.L., Buzaglo, J.S., Benson III, A.B., Cegala, D.J., Diefenbach,
586 M.A., Fleisher, L., Miller, S.M., Sulmasy, D.P., Weinfurt, K.P. and CONNECT Study

For peer review only

Research Group, 2008. Cancer patient preferences for quality and length of life. Cancer, 113(12), pp.3459-3466.

48) Bröckelmann PJ, McMullen S, Wilson JB, Mueller K, Goring S, Stamatoullas A, Zagadailov E, Gautam A, Huebner D, Dalal M, Illidge T. Patient and physician preferences for first-line treatment of classical Hodgkin lymphoma in Germany, France and the United Kingdom. British Journal of Haematology. 2019 Jan;184(2):202-14.

For peer review only

Supporting Information

S1 File. Qualitative Methods

S2 File. Additional Tables

S3 File. Additional Figures

For peer review only

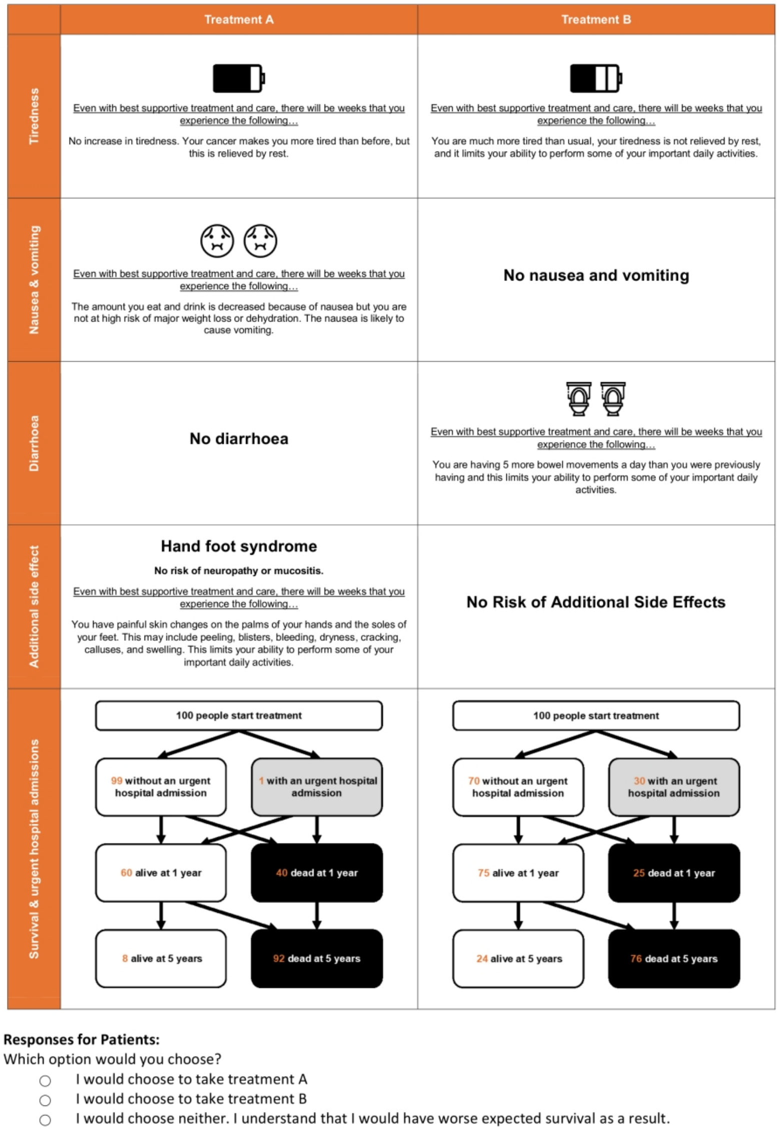

Figure 1: Example of DCE choice task (Warm-up task)

296x419mm (200 x 200 DPI)

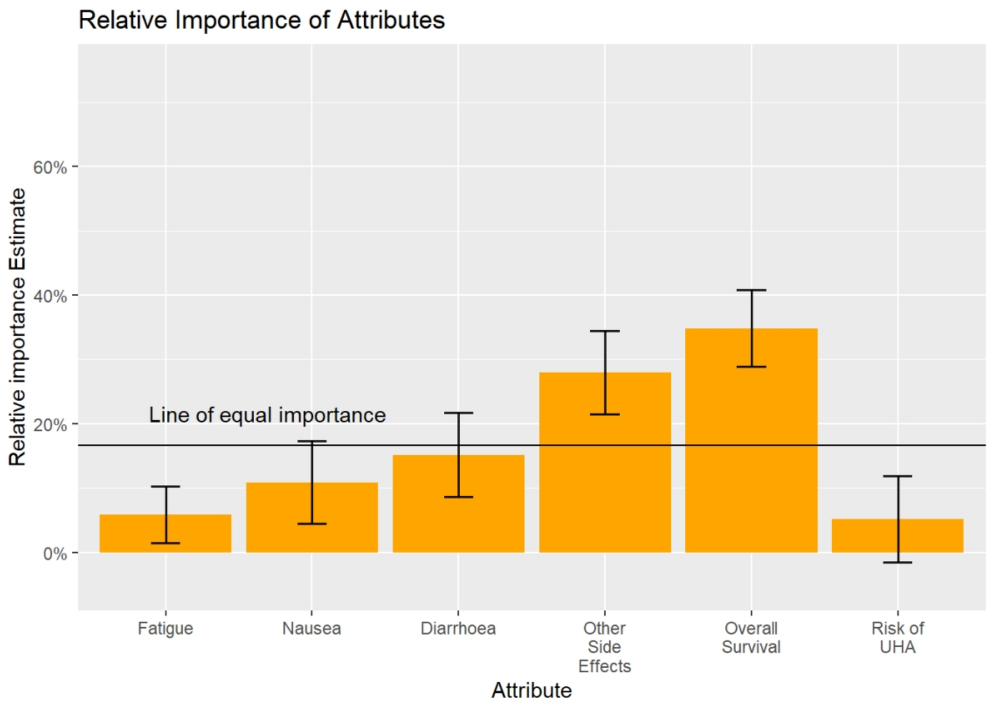

Figure 2: Relative Importance of Attributes

449x320mm (76 x 76 DPI)

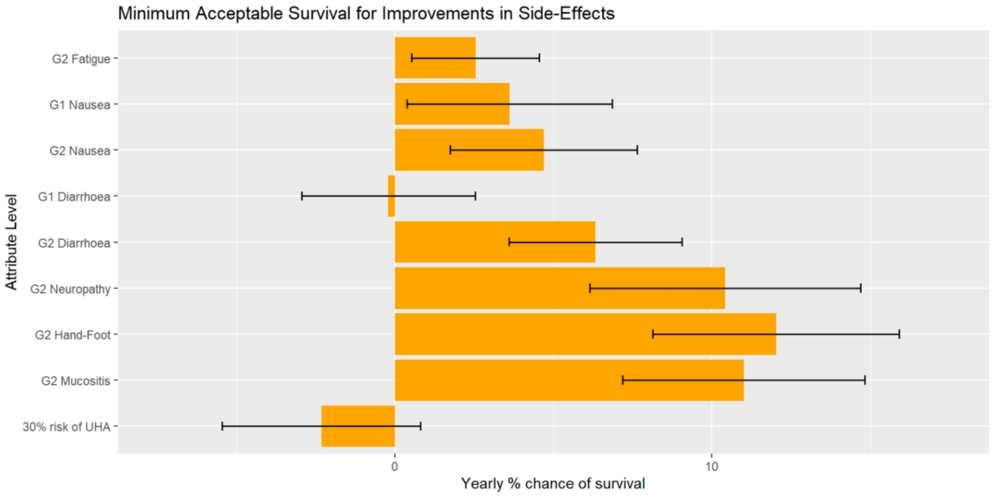

Figure 3: Minimum acceptable survival to Avoid Side effects

641x320mm (76 x 76 DPI)

Trade-offs between overall survival and side effects in the treatment of metastatic breast cancer: eliciting preferences of patients with primary and metastatic breast cancer using a discrete choice experiment'

Supporting Information 1

Qualitative Methods

Qualitative Literature Review

Embase and Medline were searched using the Ovid search engine. We aimed to identify literature which explored the patient perspective of cancer and the associated treatments. Search terms were designed to identify studies that (1) involved interviews/focus groups (2) explored patient attitudes/perspectives (3) focused on advanced or locally advanced cancer. We included all metastatic cancers given the scarcity of metastatic breast cancer-specific literature.

The search identified 434 results. Abstracts were screened and papers were excluded if they didn't reflect the underlying motivation of the search strategy. Studies were also excluded if: they focussed on an intervention which was not clinically supported or was not medicine (e.g., alternative medicine and exercise respectively); the study focus was seldom relevant to breast cancer (e.g., breathing complications brought on lung tumours). After abstract screening 83 studies remained after which 5 additional studies were excluded after reading beyond the abstract. The remaining papers were evaluated and findings which offered insight into determinants of a patient's quality of life or preference for treatment were identified. Findings were compiled and condensed into a report summarising what the available research to date suggested determining patient preferences and well-being.

Pain was among the most prominent topics of discussion. Respondents who had experienced cancer pain identified it as the most disturbing and limiting symptom of their illness (Luoma and Hakamies-Blomqvist, 2004). Patients with pain often reported extreme negative emotions (Lewis et al, 2015), loss of independence (Gibbins et al, 2014), and a desire for assisted death (Koffman et al, 2008). Other frequently explored topics included physical functioning and mobility which, as concepts, are closely linked to pain (Wilson et al, 2005). The symptoms of disease and the side effects of treatment which led to degraded physical functioning levels were identified as substantial barriers to a patient's ability to live a normal life (Gibbins et al, 2014). Extreme degradation of mobility leads to increased dependence on loved ones and carers which can create a strong sense of burden (Mak, and Elwyn, 2005). Cognitive functioning also appears to have been a topic of interest for qualitative researchers. Although cognitive functioning appears to have been a significant area of interest, many metastatic breast cancer patients rarely had symptoms, when they did, they presented as secondary disturbances or anxieties (Luoma and Hakamies-Blomqvist, 2004). Patients were willing to take medications which were associated with drowsiness to alleviate symptoms of pain (Check et al, 2017). This is evidence that patients already accept trade-offs between symptoms when considering treatments. Evidence of similar trade-offs was also found between: hot flushes and mode of administration (Fallowfield et al, 2005), expected survival and physical functioning (Check et al, 2017), and expected survival against the collective side effects of chemotherapy (Etkind et al, 2017). Evidence of trade-offs between symptoms and side effects tells us something about the importance of those toxicities, but more importantly, helps to validate the decisional context we use to frame our DCE survey questions. Other themes which featured heavily in the literature were the topics of survival, fatigue, and mode of administration, all of which are discussed in more detail in section 4 of this paper.

DCE Literature Review

The benefits of reviewing DCEs with similar motivations to our study are twofold. Firstly, they can offer insight into the importance of some of the treatment factors which we would be considering.

For peer review only

Secondly, DCEs often employ rigorous qualitative processes and their choice of attributes is likely to be of interest because their selection implicitly suggests significance. In the context of a cancer treatment DCE an attribute would be a feature of treatment which has the potential to vary between competing hypothetical treatments in a choice task. Embase and Medline were searched for DCE studies relating to patient preference for metastatic cancer treatments[1]. Search terms designed to identify DCEs mirrored those first used by Ryan and Gerard (2003). We also reincorporated the search terms used to identify metastatic cancer studies used in the qualitative literature review. Once again preliminary searches revealed that there was an insufficient body of publications to focus on metastatic breast cancer studies alone. 128 unique studies were identified in total. After screening the abstracts 60 papers met the eligibility criteria. There were 16 instances where two studies reported the results from the same DCE, in these instances the most recent publication was selected. 44 studies were identified as meeting all the criteria. Once the papers were identified work began to analyse the attributes used by the studies. The WP produced 2 key outputs of interest (1) an outline of the types of attributes used in similar past DCEs and (2) their relative importance.

Attributes were grouped into categories with similar motives. The table below outlines the attribute categories which featured in more than one DCE. There were instances where one DCE contained more than one attribute which could fit into the same category, in which instance only one was counted.

Table 1 Frequency of attribute categories included in the DCE literature review

**Attribute Category Frequency**	**n**
Administration	12
Progression Free Survival	12
Cost	8
Overall Survival	8
Pain	7
Fatigue	5
Gastrointestinal Perforation	3
Kidneys	3
Skin	3
Teeth/jaw	3
Adverse Events	2
Bone Metastases	2
Diarrhoea	2
Hospitalisation	2
Immunosuppression	2
Nausea	2
Neuropathy	2
Response rates	2
Self-care	2

Relative preference weights are measures of the importance of attributes relative to competing attributes and are conditional on the range of utility estimates for the remaining attributes (Hauber et al, 2016). A large relative preference weight suggests that an attribute has high importance in the

[1] the number metastatic breast cancer specific studies identified in preliminary searches were insufficient to justify their own review

For peer review only

context of the DCE's design. The selection of competing attributes, the range of levels for the attribute and its competitors, and framing effects (Howard and Salkeld, 2009) all determine the scale of a relative preference weight. Nevertheless, underlying preference is still a key determinant of relative preference weights and, if the considerations are accounted for, valuable inferences are possible. When making comparisons between DCEs differing study designs should be considered including decisional context, the motivations of the studies, statistical methods, and sample compositions. The complexity of these comparisons means they can't be definitively relied upon, nonetheless they are useful when consolidated with additional information from other WPs.

The main finding of the DCE literature review was the prevalence of certain attributes among the DCEs, furthermore, certain attributes tended to be associated with high relative importance between DCEs. The closely related attributes of progression free survival (PFS) and overall survival (OS) were both frequently included and tended to have high relative importance, the significance of survival and the relationship between these variations will be explored in more depth in section 4 of this paper. Pain was another category of attribute which was frequently explored and tended to be associated with high relative importance, this suggests a strong preference amongst patients to minimise suffering. It is also worth noting that, many studies appeared to be interested in patients' preferences for mode of administration, although it appeared respondents often prioritised other attributes. As a final note, the relative importance of many symptoms and side effects such as fatigue, nausea and diarrhoea differed greatly between DCEs, it was here that the limitations of making deductions from the results DCEs with different objectives were most apparent.

PACE Statement Thematic Analysis

We were granted access by the Scottish Medicines Consortium (SMC) to eight PACE statements relating to metastatic breast cancer treatments. The SMC is Scotland's advisory body for medicines, as part of their drug approval process for ultra-orphan and end-of-life medicines they invite patient and clinical representatives to meetings to discuss the benefits. These are known as Patient and Clinical Engagement (PACE) meetings. PACE meetings aim to consider all available and relevant evidence regarding new medicines including factors which traditional economic evaluation tends to overlook. We identified PACE statements as a potentially useful secondary data resource for our research since their focus is on the needs of the patient. Another advantage is that PACE statements are a relatively recent innovation meaning they tend to present up-to-date information. Between Oct 2014 and Oct 2018, eight PACE meetings were convened for medicines seeking reimbursement for the treatment of metastatic breast cancer. We conducted a formal thematic analysis (Braun and Clarke, 2012) of the PACE statements which focussed on the positive and negative impacts of treatment as well the insights into patient priorities.

We were able to identify six core themes which were composed of additional sub-themes (see figure below). Themes were not mutually exclusive, meaning there is some degree of overlap between themes. Two of the themes represent what we came to understand as the core goals of patients according to the data, these were 'Ability to live a normal life' and 'Survival'; treatments were praised repeatedly by committees for their ability to improve these two outcomes. When consulting the evidence from the PACE analysis it should be considered that they are designed to consider externalities and not just the direct effect on patients. Specifically, PACE guidelines request that respondents discuss the effect of disease and treatment on the family and carers. This explains the prominence of the 'effect on close ones' theme which is often featured in the form of considering perspectives outside of the patients. Although the findings were interesting for our research, we decided to focus on the perspective of the patient. So naturally, this theme emerged. A key disadvantage of PACE statements was their tendency to talk broadly and generally about symptoms and side effects. For our research, we were interested in patients' preferences for specific symptoms and side effects, but the lack of detail meant little could be deduced about which common side effects were more troublesome than others. It should also be noted that PACE statements are rarely critical of

For peer review only

emerging drugs. The general feeling from the PACE statements was that participants were keen to highlight the benefits of emerging drugs. There was a positive bias that we had to consider when toxicities and benefits associated with the treatment in question were mentioned

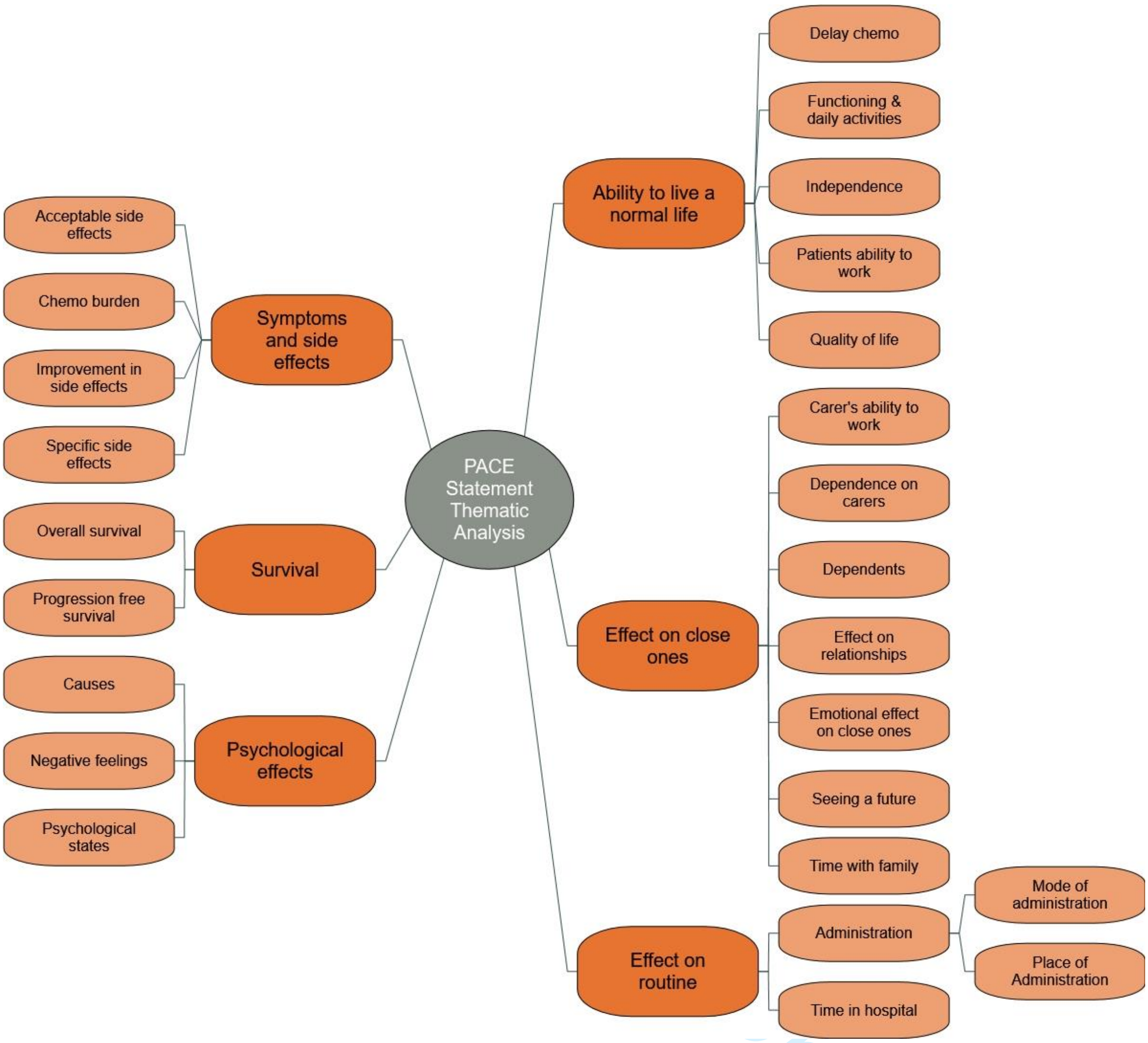

Figure 1 – Results from thematic analysis of breast cancer PACE data

Patient Interviews

The richest data from the early stages of the project emerged from the semi-structured interviews we conducted with 9 patients diagnosed with metastatic breast cancer. Women with secondary breast cases with experience of multiple treatments and who were currently living in the Lothian area were contacted by a research nurse and invited to participate in a face-to-face interview at an agreed location, either a cancer charity premises or the patient's home. We wanted to adopt a flexible strategy where we could adapt individual interviews and our broader strategies as our understanding of patient preferences and experiences developed. Grounded theory (Strauss and Corbin, 1994) is a qualitative methodology that encourages a flexible strategy, however, conventional recommendations state that interviewers should be mostly ignorant about the topic being explored so that bias does not interfere with the formulation of theories. Given that we already had considerable knowledge of the experiences of breast cancer patients, owing to ongoing research and professional experience, we instead opted to conduct interviews according to the informed grounded theory approach (Thornberg,

For peer review only - http://bmjopen.bmj.com/site/about/guidelines.xhtml

2012). This adaptation of the grounded theory methodology allowed us to incorporate our prior knowledge in the traditional grounded theory approach whilst being aware of bias and remaining open to new ideas. An interview plan was formulated which provided structure whilst allowing for deviation and elaboration. The three core areas of focus were (1) patient history – patients were invited to discuss the treatments they had received and reflect on their experiences with them (2) treatment decision making – patient were asked how they remember decisions about treatment being and to reflect on the extent of their own involvement (3) experience with treatment and disease – patients were asked to reflect on their lived experience of their disease and their treatment and how it affected them.

To summarise the broader findings: There was a general attitude that more treatment was generally better and that listening to the advice of health professionals is the best thing one can do. There was a large degree of variation in terms of the specific side effects that patients' experiences and to what extent. This is likely a consequence of the wide range of secondary malignancies and the treatments received. Several patients mentioned suffering very little from symptoms and side effects since their secondary diagnosis. There was a prevailing negative attitude towards chemotherapy and its associated toxicities. The two primary goals of treatment appeared to be life extension and minimising disruption to everyday life. The interviews helped us to understand the broader goals of patients as well as their self-reported attitudes and behaviours regarding shared decision making. The richest findings however related to discussions concerning specific symptoms and side effects, evidence from these discussions will feature heavily in section 4 of this paper.

For peer review only

References

Braun, V. and Clarke, V., 2012. Thematic analysis.

Check, D. K., Park, E. M., Reeder-Hayes, K. E., Mayer, D. K., Deal, A. M., Yopp, J. M., … Hanson, L. C. (2017). Concerns underlying treatment preferences of advanced cancer patients with children. Psycho-Oncology, 26(10), 1491–1497.

Etkind, S.N., Bristowe, K., Bailey, K., Selman, L.E. and Murtagh, F.E., 2017. How does uncertainty shape patient experience in advanced illness? A secondary analysis of qualitative data. Palliative medicine, 31(2), pp.171-180.

Gibbins, J., Bhatia, R., Forbes, K., & Reid, C. M. (2014). What do patients with advanced incurable cancer want from the management of their pain? A qualitative study. Palliative Medicine, 28(1), 71–78.

Hauber, A.B., González, J.M., Groothuis-Oudshoorn, C.G., Prior, T., Marshall, D.A., Cunningham, C., IJzerman, M.J. and Bridges, J.F., 2016. Statistical methods for the analysis of discrete choice experiments: a report of the ISPOR Conjoint Analysis Good Research Practices Task Force. Value in health, 19(4), pp.300-315.

Howard, K. and Salkeld, G., 2009. Does attribute framing in discrete choice experiments influence willingness to pay? Results from a discrete choice experiment in screening for colorectal cancer. Value in health, 12(2), pp.354-363.

Koffman, J., Morgan, M., Edmonds, P., Speck, P., & Higginson, I. (2008). Cultural meanings of pain: A qualitative study of Black Caribbean and White British patients with advanced cancer. Palliative Medicine, 22(4), 350–359.

Lewis, S., Yee, J., Kilbreath, S. and Willis, K., 2015. A qualitative study of women's experiences of healthcare, treatment and support for metastatic breast cancer. The Breast, 24(3), pp.242-247.

Luoma, M.L. and Hakamies-Blomqvist, L., 2004. The meaning of quality of life in patients being treated for advanced breast cancer: a qualitative study. Psycho-Oncology: Journal of the Psychological, Social and Behavioral Dimensions of Cancer, 13(10), pp.729-739.

Mak, Y.Y.W. and Elwyn, G., 2005. Voices of the terminally ill: uncovering the meaning of desire for euthanasia. Palliative medicine, 19(4), pp.343-350.

Ryan, M. and Gerard, K., 2003. Using discrete choice experiments to value health care programmes: current practice and. Applied health economics and health policy, 2(1), pp.55-64.Hauber, A.B.,

Strauss, A. and Corbin, J., 1994. Grounded theory methodology. Handbook of qualitative research, 17, pp.273-85.

Thornberg, R., 2012. Informed grounded theory. Scandinavian Journal of Educational Research, 56(3), pp.243-259.

Wilson, K. G., Curran, D., & McPherson, C. J. (2005). A burden to others: A common source of distress for the terminally ill. Cognitive Behaviour Therapy, 34(2), 115–123

For peer review only

Supporting Information 2

Table A1 Presentation of Side Effects Attribute Levels to Respondents

**Tiredness**	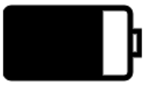 Even with best supportive treatment and care, there will be weeks that you experience the following… No increase in tiredness. Your cancer makes you more tired than before, but this is relieved by rest.	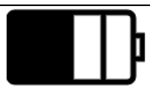 Even with best supportive treatment and care, there will be weeks that you experience the following… You are much more tired than usual, your tiredness is not relieved by rest, and it limits your ability to perform some of your important daily activities.	
**Nausea and Vomiting**	**No nausea and vomiting**	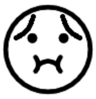 Even with best supportive treatment and care, there will be weeks that you experience the following… You have lost your appetite due to nausea, but not enough to change the amount you eat. Your nausea may cause some vomiting.	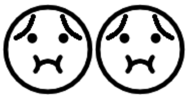 Even with best supportive treatment and care, there will be weeks that you experience the following… The amount you eat and drink is decreased because of nausea but you are not at high risk of major weight loss or dehydration. The nausea is likely to cause vomiting.
**Diarrhoea**	**No diarrhoea**	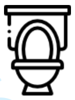 Even with best supportive treatment and care, there will be weeks that you experience the following… You are having 2 more bowel movements a day than you were previously having.	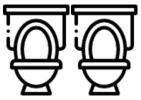 Even with best supportive treatment and care, there will be weeks that you experience the following… You are having 5 more bowel movements a day than you were previously having and this limits your ability to perform some of your important daily activities.
**Additional Side effect**	**Peripheral neuropathy No risk of hand foot syndrome or mucositis.** Even with best supportive treatment and care, there will be weeks that you experience the following… You have numbness and tingling in the feet or hands and occasionally burning, stabbing or shooting pain in affected areas. This limits your ability to perform some of your important daily activities.	**Hand foot syndrome No risk of neuropathy or mucositis.** Even with best supportive treatment and care, there will be weeks that you experience the following… You have painful skin changes on the palms of your hands and the soles of your feet. This may include peeling, blisters, bleeding, dryness, cracking, calluses, and swelling. This limits your ability to perform some of your important daily activities.	**Mucositis No risk of neuropathy or hand foot syndrome.** Even with best supportive treatment and care, there will be weeks that you experience the following… Your mouth becomes sore and inflamed. You have ulcers which are painful and mean you are unable to eat spicy, acidic, and crunchy foods such as crisps.
	**No risk of neuropathy, hand foot syndrome or mucositis**		

For peer review only

Table A2 Respondent characteristics

**Diagnosis**	**n**
Metastatic breast cancer	72
Primary breast cancer	33
**Gender**	
Female	105
Male	0
**Age**	
30-39	8
40-49	19
50-59	47
60-69	25
70-79	6

For peer review only

Table A3 Multinomial Results – Main Specification

		Estimate	p	95% CI Lower bound	95% CI Upper bound	Relative attribute importance	Minimum acceptable survival
Alternative Specific Constant	Treatment	0.9598	0.0006	0.4136	1.5060	-	-
Fatigue	Grade 2 fatigue	-0.2899	0.0089	-0.5073	-0.0726	0.0658	2.8017
Nausea	Grade 1 nausea	-0.3070	0.1021	-0.6750	0.0610	0.0951	2.9665 N.S.
	Grade 2 nausea	-0.4192	0.0232	-0.7811	-0.0573		4.0503
Diarrhoea	Grade 1 diarrhoea	0.0696	0.6425	-0.2242	0.3636	0.1536	-0.6734 N.S.
	Grade 2 diarrhoea	-0.6076	0.0011	-0.9715	-0.2438		5.8714
Additional side effects	Grade 2 peripheral neuropathy	-1.070	0.0000	-1.4654	-0.6748	0.2693	10.3399
	Grade 2 hand foot syndrome	-1.1873	0.0000	-1.5759	-0.7987		11.4723
	Grade 2 mucositis	-1.1264	0.0000	-1.4830	-0.7698		10.8842
Overall survival	Annual probability of survival	0.1035	0.0000	0.0764	0.1305	0.3521	-
Urgent Hospital Admission	Probability of urgent hospital admission in the first year of treatment	0.0097	0.0589	-0.0004	0.0198	0.0640 N.S.	-2.8223 N.S.(for 30% level)
**Model statistics**							
Number of individuals	105						
Observations	601						
Log likelihood	-431.59						
Bayesian info criterion	933.5637						

N.S. not significant

Table A4 Multinomial Results – Excluding Treat variable and paramatarising Overall Survival as Dummy Variables

Attribute	Level	Estimate	p	95% CI Lower bound	95% CI Upper bound
Fatigue	Grade 2 fatigue	-0.2887	0.0136	-0.5179	-0.0595
Nausea	Grade 1 nausea	-0.3084	0.1080	-0.6844	0.0677
	Grade 2 nausea	-0.4194	0.0232	-0.7814	-0.0574
Diarrhoea	Grade 1 diarrhoea	0.0668	0.6959	-0.2682	0.4019
	Grade 2 diarrhoea	-0.6036	0.0066	-1.0391	-0.1680
Additional side effects	Grade 2 peripheral neuropathy	-1.0758	0.0000	-1.5941	-0.5576
	Grade 2 hand foot syndrome	-1.1897	0.0000	-1.6034	-0.7761
	Grade 2 mucositis	-1.1269	0.0000	-1.4844	-0.7694
Overall survival (Annual probability of survival)	60%	2.5175	0.0000	1.9034	3.1316
	65%	3.0212	0.0000	2.2538	3.7887
	75%	4.0641	0.0000	3.2953	4.8329
Urgent Hospital Admission	Probability of urgent hospital admission in the first year of treatment	0.0100	0.2467	-0.0069	0.0268
**Model statistics**					
Number of individuals	105				
Observations	601				
Log likelihood	-431.59				
Bayesian info criterion	939.96				

Trade-offs between overall survival and side effects in the treatment of metastatic breast cancer: eliciting preferences of patients with primary and metastatic breast cancer using a discrete choice experiment’

Supporting Information 3

Figure A1 Comparison of relative importance estimates from multinomial logit models between the entire sample and ‘survival non-traders’ (sample excluding survival non-traders, N=71)

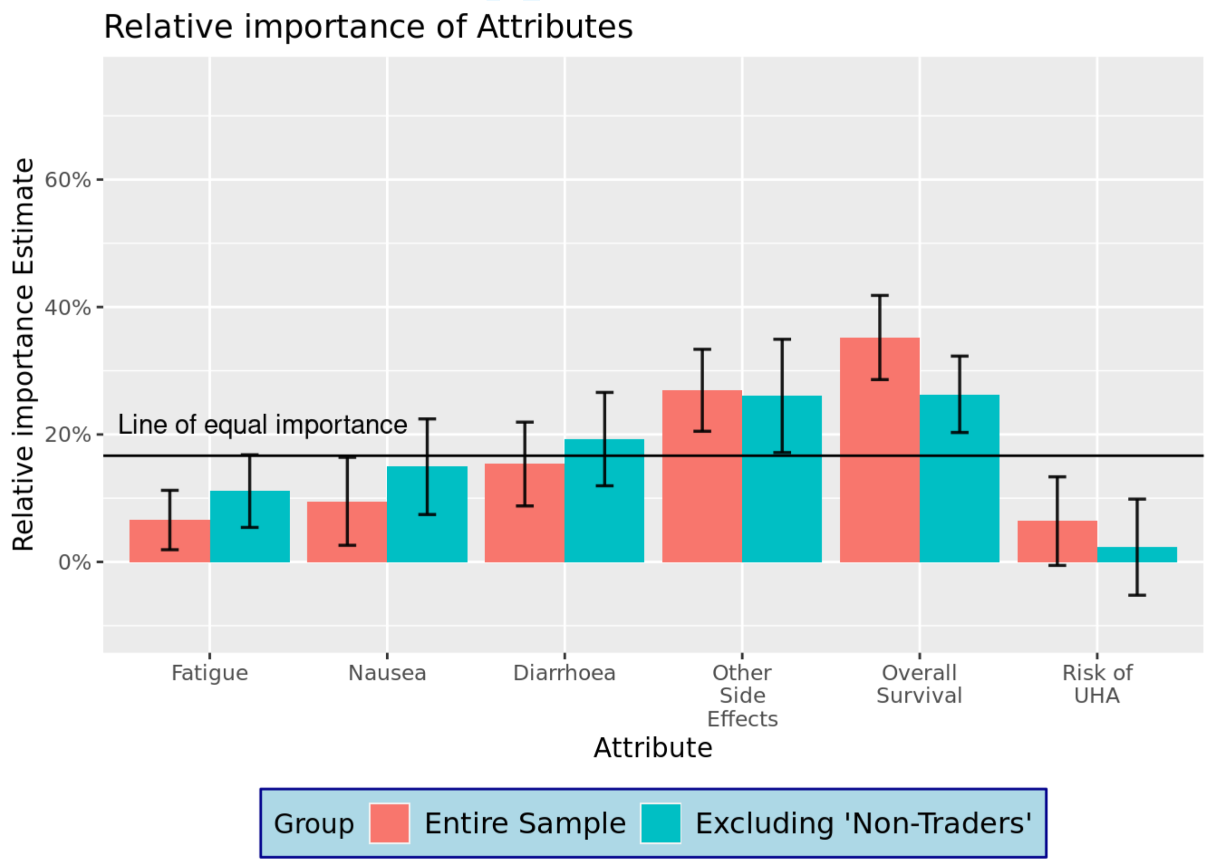

Error bars show 95% confidence interval using delta method standard errors

Figure A2 Comparison of minimum acceptable survival from multinomial logit models between the entire sample and 'survival non-traders' (sample excluding survival non-traders, N=71)

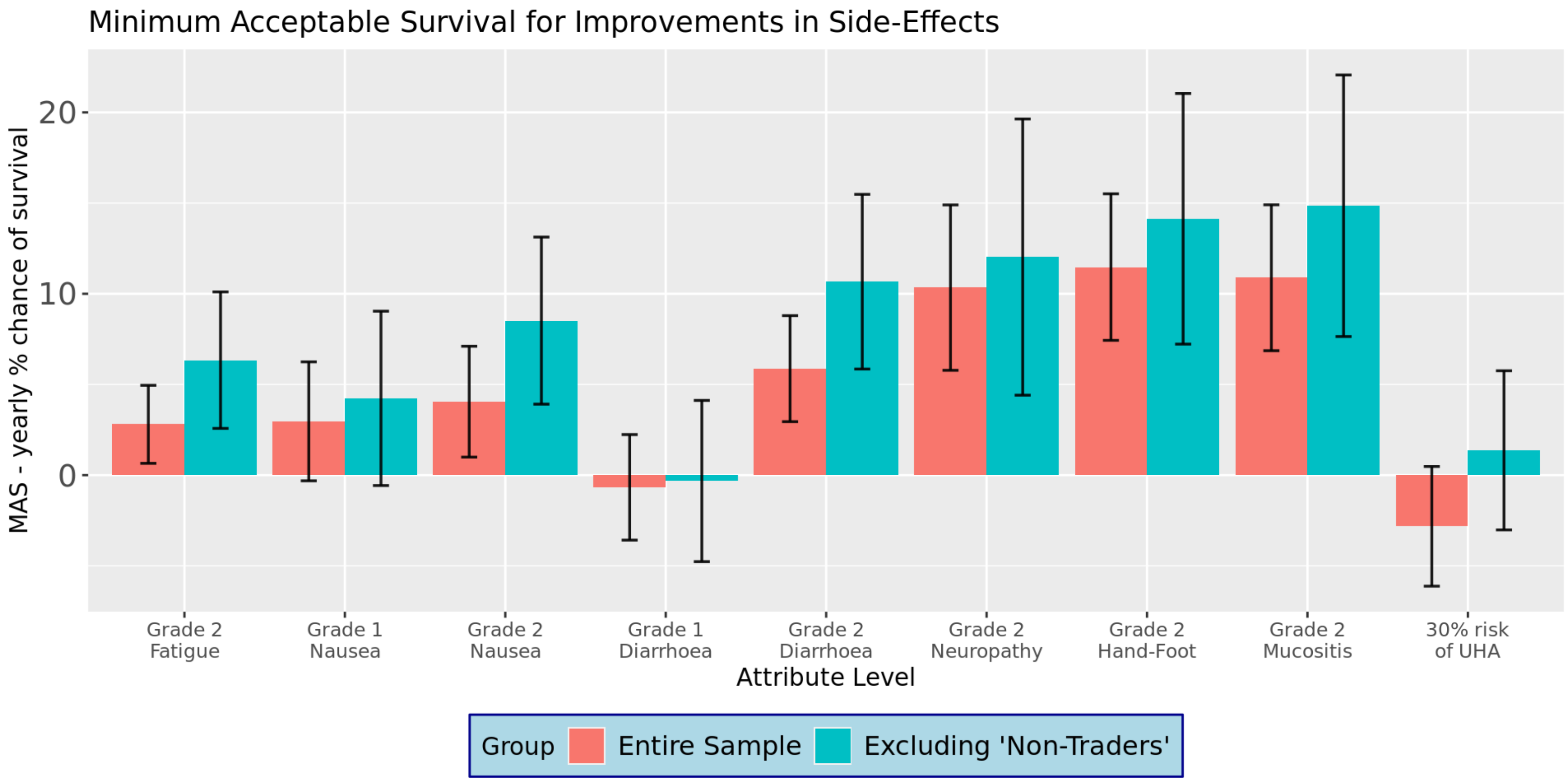

Error bars show 95% confidence interval using delta method standard errors

Figure A3 Comparison of Relative importance estimates from multinomial logit models between the metastatic breast cancer sample (N=72) and the primary breast cancer sample (N=33)

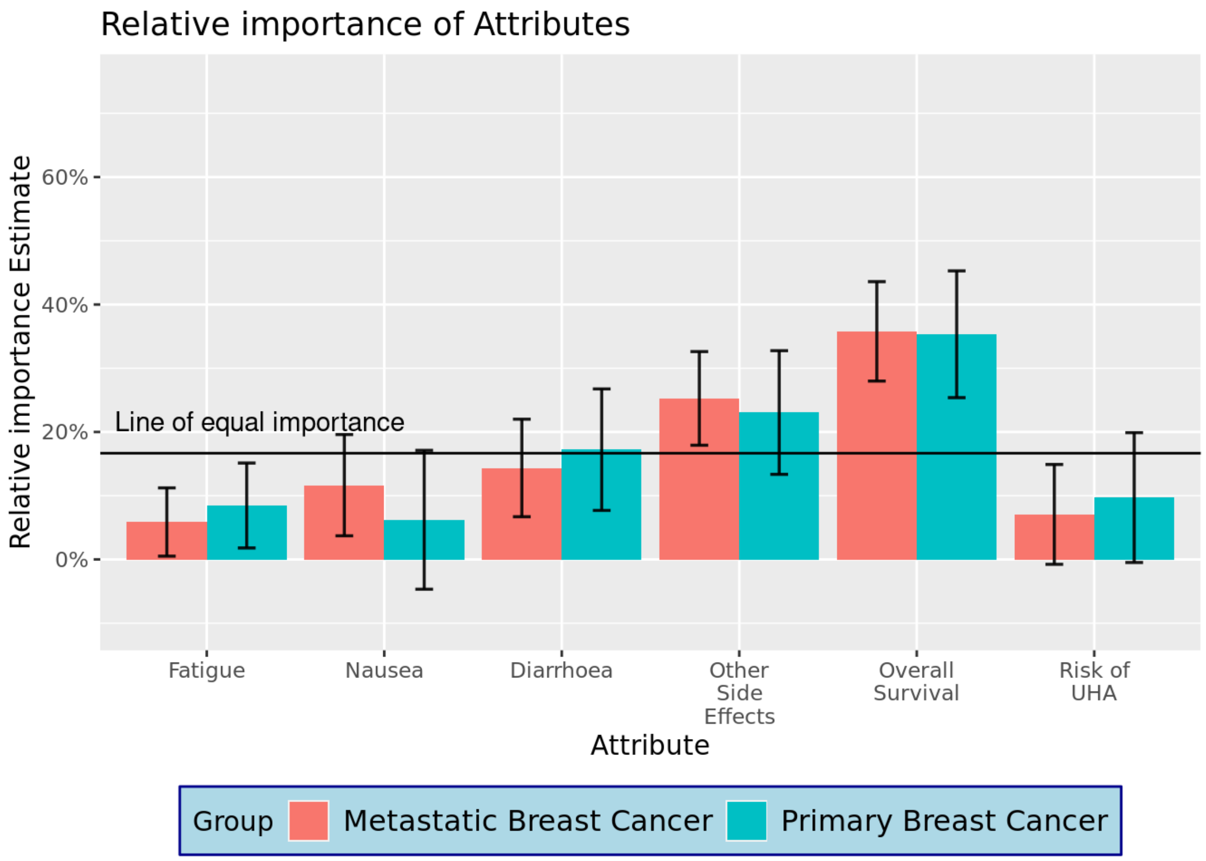

Error bars show 95% confidence interval using delta method standard errors

Figure A4 Comparison of Relative importance Estimates Between the Metastatic Breast Cancer Sample (N=72) and the Primary Breast Cancer Sample (N=33)

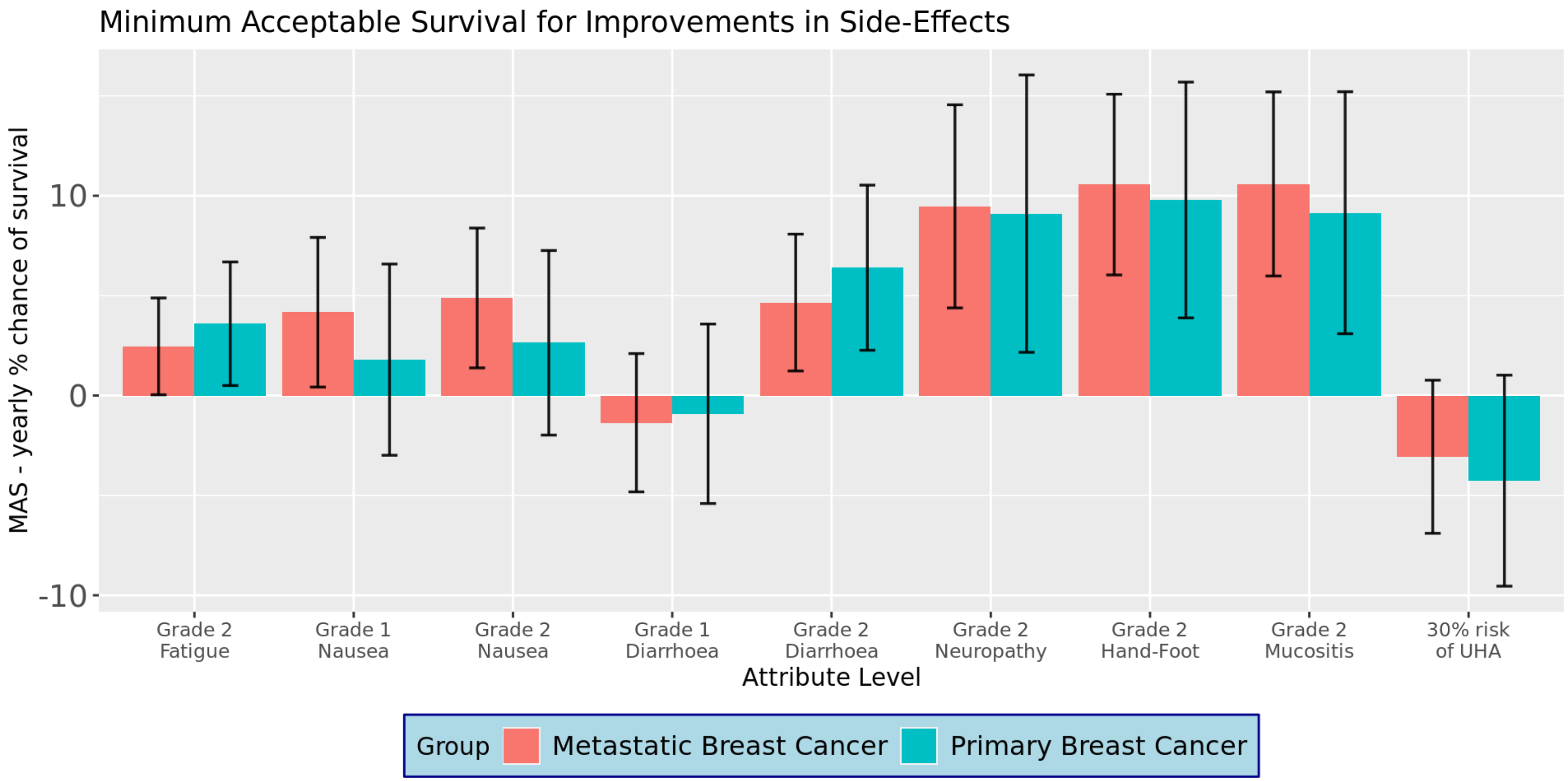

Error bars show 95% confidence interval using delta method standard errors
